
JOURNAL INFORMATION
==============================
NLM Title Abbreviation: J Int AIDS Soc
Journal ID: J Int AIDS Soc
NLM Journal ID: 101478566
Journal ID: JIAS

Journal of the International AIDS Society

EISSN: 1758-2652
Publisher: Wiley

ARTICLE INFORMATION
==============================
PMCID: PMC3499903
DOI: 10.7448/IAS.15.5.18442
Article ID: 18442
Article version: 1
Subjects: AIDS2012 Abstract Supplement

Track D Social Science, Human Rights and Political Science

Electronic publication date: 2012 Oct 22
Publication date: 2012
Volume: 15
Issue: Suppl 3
Electronic Location ID: 18442
Copyright: © 2012 Track D Social Science, Human Rights and Political Science
Copyright year: 2012
License: This is an open-access article distributed under the terms of the Creative Commons Attribution License, which permits unrestricted use, distribution, and reproduction in any medium, provided the original work is properly cited.
License URL: https://creativecommons.org/licenses/by/2.0/

==============================
JOURNAL INFORMATION
==============================
NLM Title Abbreviation: J Int AIDS Soc
Journal ID: J Int AIDS Soc
NLM Journal ID: 101478566
Journal ID: JIAS

Journal of the International AIDS Society

EISSN: 1758-2652
Publisher: Wiley

ARTICLE INFORMATION
==============================
Article ID: 18442-001
Subjects: D1 - Combination prevention programmes

MOAD0405: Efficacy of combined sexual and injection risk reduction interventions for female sex workers on the Mexico-US border: differential effects in the presence of a community-wide structural intervention

Strathdee S. 1 §
Lozada R. 2
Martinez G. 3
Rangel G. 4
Staines H. 5
Abramovitz D. 6
Vera A. 7
Magis-Rodriguez C. 8
Patterson T. 9
Proyecto Mujer Mas Segura
1 University of California San Diego School of Medicine, Medicine, La Jolla, United States
2 ISESALUD, Tijuana, Mexico
3 SADEC-FEMAP, Ciudad Juarez, Mexico
4 COLEF, Tijuana, Mexico
5 Universidad Autonoma de Ciudad Juarez, Ciudad Juarez, Mexico
6 University of California, San Diego, Medicine, La Jolla, United States
7 Universidad Autonoma de Baja California, Tijuana, Mexico
8 CISIDAT, Mexico City, Mexico
9 University of California, San Diego, Psychiatry, La Jolla, United States
§ Presenting author email: sstrathdee@ucsd.edu
Electronic publication date: 2012 Oct 22
Publication date: 2012
Volume: 15
Issue: Suppl 3
Electronic Location ID: 18442-001
Copyright: © 2012 S. Strathdee et al.
Copyright year: 2012
License: This is an open-access article distributed under the terms of the Creative Commons Attribution License, which permits unrestricted use, distribution, and reproduction in any medium, provided the original work is properly cited.
License URL: http://creativecommons.org/licenses/by/2.0/

JOURNAL INFORMATION
==============================
NLM Title Abbreviation: J Int AIDS Soc
Journal ID: J Int AIDS Soc
NLM Journal ID: 101478566
Journal ID: JIAS

Journal of the International AIDS Society

EISSN: 1758-2652
Publisher: Wiley

ARTICLE INFORMATION
==============================
Article ID: 18442-002
Subjects: D1 - Combination prevention programmes

MOAE0202: Is treatment as prevention the new game-changer? Costs and effectiveness

Bärnighausen T. 1 2 §
Bloom D. 1
Humair S. 1 3
1 Harvard School of Public Health, Boston, United States
2 Africa Centre for Health and Population Studies, University of KwaZulu-Natal, Mtubetuba, South Africa
3 School of Science and Engineering, Lahore University of Management Sciences, Lahore, Pakistan
§ Presenting author email: tbaernig@hsph.harvard.edu
Electronic publication date: 2012 Oct 22
Publication date: 2012
Volume: 15
Issue: Suppl 3
Electronic Location ID: 18442-002
Copyright: © 2012 T. Bärnighausen et al.
Copyright year: 2012
License: This is an open-access article distributed under the terms of the Creative Commons Attribution License, which permits unrestricted use, distribution, and reproduction in any medium, provided the original work is properly cited.
License URL: http://creativecommons.org/licenses/by/2.0/

JOURNAL INFORMATION
==============================
NLM Title Abbreviation: J Int AIDS Soc
Journal ID: J Int AIDS Soc
NLM Journal ID: 101478566
Journal ID: JIAS

Journal of the International AIDS Society

EISSN: 1758-2652
Publisher: Wiley

ARTICLE INFORMATION
==============================
Article ID: 18442-003
Subjects: D1 - Combination prevention programmes

WEAD0302: Total Control of Epidemic (TCE) program of Humana People to People: a community driven response to the fight against AIDS

Lichtenberg M. 1 §
Hansen I. 2
Mukhopadhyay S.M. 3
1 Humana People to People / Planet Aid, Inc., International Partnerships, Elkridge, United States
2 Humana People to People, Shamva, Zimbabwe, HQ
3 Humana People to People India (HPPI), New Delhi, India
§ Presenting author email: marie.lichtenberg@gmail.com
Electronic publication date: 2012 Oct 22
Publication date: 2012
Volume: 15
Issue: Suppl 3
Electronic Location ID: 18442-003
Copyright: © 2012 M. Lichtenberg et al.
Copyright year: 2012
License: This is an open-access article distributed under the terms of the Creative Commons Attribution License, which permits unrestricted use, distribution, and reproduction in any medium, provided the original work is properly cited.
License URL: http://creativecommons.org/licenses/by/2.0/

JOURNAL INFORMATION
==============================
NLM Title Abbreviation: J Int AIDS Soc
Journal ID: J Int AIDS Soc
NLM Journal ID: 101478566
Journal ID: JIAS

Journal of the International AIDS Society

EISSN: 1758-2652
Publisher: Wiley

ARTICLE INFORMATION
==============================
Article ID: 18442-004
Subjects: D1 - Combination prevention programmes

WEAD0303: Phenomenal woman: the development of a program with the dual goals of HIV and substance abuse relapse prevention

Irizarry C. 1
Jones C. 1
West C. 2 §
Adjei B. 3
Nordlund D. 1
1 Greenhope Services for Women, INC, New York, United States
2 Sankofa Services, Atlanta, United States
3 Independent Evaluation Consultant, Rockville, United States
§ Presenting author email: cacwest@bellsouth.net
Electronic publication date: 2012 Oct 22
Publication date: 2012
Volume: 15
Issue: Suppl 3
Electronic Location ID: 18442-004
Copyright: © 2012 C. West et al.
Copyright year: 2012
License: This is an open-access article distributed under the terms of the Creative Commons Attribution License, which permits unrestricted use, distribution, and reproduction in any medium, provided the original work is properly cited.
License URL: http://creativecommons.org/licenses/by/2.0/

JOURNAL INFORMATION
==============================
NLM Title Abbreviation: J Int AIDS Soc
Journal ID: J Int AIDS Soc
NLM Journal ID: 101478566
Journal ID: JIAS

Journal of the International AIDS Society

EISSN: 1758-2652
Publisher: Wiley

ARTICLE INFORMATION
==============================
Article ID: 18442-005
Subjects: D2 - Behaviourial and social research on risk reduction interventions

TUAD0302: HIV prevention needs of transgender sex workers in Serbia

Ilic D. 1 §
1 Association against AIDS - JAZAS, Belgrade, Serbia
§ Presenting author email: drilic@sezampro.rs
Electronic publication date: 2012 Oct 22
Publication date: 2012
Volume: 15
Issue: Suppl 3
Electronic Location ID: 18442-005
Copyright: © 2012 D. Ilic et al.
Copyright year: 2012
License: This is an open-access article distributed under the terms of the Creative Commons Attribution License, which permits unrestricted use, distribution, and reproduction in any medium, provided the original work is properly cited.
License URL: http://creativecommons.org/licenses/by/2.0/

JOURNAL INFORMATION
==============================
NLM Title Abbreviation: J Int AIDS Soc
Journal ID: J Int AIDS Soc
NLM Journal ID: 101478566
Journal ID: JIAS

Journal of the International AIDS Society

EISSN: 1758-2652
Publisher: Wiley

ARTICLE INFORMATION
==============================
Article ID: 18442-006
Subjects: D2 - Behaviourial and social research on risk reduction interventions

WEAD0301: Behaviour change and associated factors among female sex workers in Kenya

Nyagero J. 1 §
Wangila S. 2
Kutai V. 2
Olango S. 2
1 Africa Medical and Research Foundation, Health Programmes Development Directorate, Nairobi, Kenya
2 AMREF, Nairobi, Kenya
§ Presenting author email: josephat.nyagero@amref.org
Electronic publication date: 2012 Oct 22
Publication date: 2012
Volume: 15
Issue: Suppl 3
Electronic Location ID: 18442-006
Copyright: © 2012 J. Nyagero et al.
Copyright year: 2012
License: This is an open-access article distributed under the terms of the Creative Commons Attribution License, which permits unrestricted use, distribution, and reproduction in any medium, provided the original work is properly cited.
License URL: http://creativecommons.org/licenses/by/2.0/

JOURNAL INFORMATION
==============================
NLM Title Abbreviation: J Int AIDS Soc
Journal ID: J Int AIDS Soc
NLM Journal ID: 101478566
Journal ID: JIAS

Journal of the International AIDS Society

EISSN: 1758-2652
Publisher: Wiley

ARTICLE INFORMATION
==============================
Article ID: 18442-007
Subjects: D2 - Behaviourial and social research on risk reduction interventions

WEAD0304: Determinants of condom use in South Africa

Matseke G. 1 §
Simbayi L. 2
Wabiri N. 1
Ncitakalo N. 2
1 Human Sciences Research Council, Pretoria, South Africa
2 Human Sciences Research Council, Cape Town, South Africa
§ Presenting author email: gmatseke@hsrc.ac.za
Electronic publication date: 2012 Oct 22
Publication date: 2012
Volume: 15
Issue: Suppl 3
Electronic Location ID: 18442-007
Copyright: © 2012 G. Matseke et al.
Copyright year: 2012
License: This is an open-access article distributed under the terms of the Creative Commons Attribution License, which permits unrestricted use, distribution, and reproduction in any medium, provided the original work is properly cited.
License URL: http://creativecommons.org/licenses/by/2.0/

JOURNAL INFORMATION
==============================
NLM Title Abbreviation: J Int AIDS Soc
Journal ID: J Int AIDS Soc
NLM Journal ID: 101478566
Journal ID: JIAS

Journal of the International AIDS Society

EISSN: 1758-2652
Publisher: Wiley

ARTICLE INFORMATION
==============================
Article ID: 18442-008
Subjects: D2 - Behaviourial and social research on risk reduction interventions

THAD0301: Framing HIV testing messages for urban and rural audiences: evidence from field experiment in northwest Ethiopia

Bekalu M.A. 1 2 §
Eggermont S. 1
1 Katholieke Universiteit Leuven, Belgium, School for Mass Communication Research, Leuven, Belgium
2 Bahir Dar University, Bahir Dar, Ethiopia
§ Presenting author email: mesfiab@yahoo.com
Electronic publication date: 2012 Oct 22
Publication date: 2012
Volume: 15
Issue: Suppl 3
Electronic Location ID: 18442-008
Copyright: © 2012 M.A. Bekalu et al.
Copyright year: 2012
License: This is an open-access article distributed under the terms of the Creative Commons Attribution License, which permits unrestricted use, distribution, and reproduction in any medium, provided the original work is properly cited.
License URL: http://creativecommons.org/licenses/by/2.0/

JOURNAL INFORMATION
==============================
NLM Title Abbreviation: J Int AIDS Soc
Journal ID: J Int AIDS Soc
NLM Journal ID: 101478566
Journal ID: JIAS

Journal of the International AIDS Society

EISSN: 1758-2652
Publisher: Wiley

ARTICLE INFORMATION
==============================
Article ID: 18442-009
Subjects: D2 - Behaviourial and social research on risk reduction interventions

THAD0302: ‘AIDS is gone. That's what they think’ College and university youth in Botswana share their thoughts on HIV, risk, behaviours, needs and interventions

Godwaldt M. 1 §
1 World University Service of Canada, Gaborone, Botswana
§ Presenting author email: melissa@wusc.co.bw
Electronic publication date: 2012 Oct 22
Publication date: 2012
Volume: 15
Issue: Suppl 3
Electronic Location ID: 18442-009
Copyright: © 2012 M. Godwaldt et al.
Copyright year: 2012
License: This is an open-access article distributed under the terms of the Creative Commons Attribution License, which permits unrestricted use, distribution, and reproduction in any medium, provided the original work is properly cited.
License URL: http://creativecommons.org/licenses/by/2.0/

JOURNAL INFORMATION
==============================
NLM Title Abbreviation: J Int AIDS Soc
Journal ID: J Int AIDS Soc
NLM Journal ID: 101478566
Journal ID: JIAS

Journal of the International AIDS Society

EISSN: 1758-2652
Publisher: Wiley

ARTICLE INFORMATION
==============================
Article ID: 18442-010
Subjects: D2 - Behaviourial and social research on risk reduction interventions

FRLBD01: Effect of a national social cash transfer program on HIV risk behavior in Kenya

Handa S. 1 §
Pettifor A. 2
Thirumurthy H. 3
Halpern C. 4
1 University of North Carolina-Chapel Hill, Public Policy and Carolina Population Center, Chapel Hill, United States
2 University of North Carolina-Chapel Hill, Epidemiology, Chapel Hill, United States
3 University of North Carolina-Chapel Hill, Health Policy and Management, Chapel Hill, United States
4 University of North Carolina-Chapel HillMaternal and Child Health, Chapel Hill, United States
§ Presenting author email: shanda@email.unc.edu
Electronic publication date: 2012 Oct 22
Publication date: 2012
Volume: 15
Issue: Suppl 3
Electronic Location ID: 18442-010
Copyright: © 2012 S. Handa et al.
Copyright year: 2012
License: This is an open-access article distributed under the terms of the Creative Commons Attribution License, which permits unrestricted use, distribution, and reproduction in any medium, provided the original work is properly cited.
License URL: http://creativecommons.org/licenses/by/2.0/

JOURNAL INFORMATION
==============================
NLM Title Abbreviation: J Int AIDS Soc
Journal ID: J Int AIDS Soc
NLM Journal ID: 101478566
Journal ID: JIAS

Journal of the International AIDS Society

EISSN: 1758-2652
Publisher: Wiley

ARTICLE INFORMATION
==============================
Article ID: 18442-011
Subjects: D2 - Behaviourial and social research on risk reduction interventions

MOPDD0101: A pilot South African worksite-based parenting program: preliminary effects on parent-child communication about sex and HIV

Bogart L. 1 2 §
Skinner D. 3
Thurston I. 1 2
Toefy Y. 3
Klein D. 1
Wachman M. 1
Schuster M. 1 2
1 Children's Hospital Boston, Division of General Pediatrics, Boston, United States
2 Harvard Medical School, Boston, United States
3 Stellenbosch University, Tygerberg, South Africa
§ Presenting author email: laura.bogart@childrens.harvard.edu
Electronic publication date: 2012 Oct 22
Publication date: 2012
Volume: 15
Issue: Suppl 3
Electronic Location ID: 18442-011
Copyright: © 2012 L. Bogart et al.
Copyright year: 2012
License: This is an open-access article distributed under the terms of the Creative Commons Attribution License, which permits unrestricted use, distribution, and reproduction in any medium, provided the original work is properly cited.
License URL: http://creativecommons.org/licenses/by/2.0/

JOURNAL INFORMATION
==============================
NLM Title Abbreviation: J Int AIDS Soc
Journal ID: J Int AIDS Soc
NLM Journal ID: 101478566
Journal ID: JIAS

Journal of the International AIDS Society

EISSN: 1758-2652
Publisher: Wiley

ARTICLE INFORMATION
==============================
Article ID: 18442-012
Subjects: D2 - Behaviourial and social research on risk reduction interventions

WEPDD0101: The relationship between timing of childhood sexual abuse and subsequent HIV risky behaviours in severely mentally ill adults

Bendezu J. 1 §
Berger-Greenstein J. 1
Richardson M. 2
Reid K. 1
Wolfe J. 1
Mainville C. 1
Bacic J. 3
Brady S. 1
1 Boston University, Mental Health and Behavioral Medicine Program, Boston, United States
2 Boston University, Psychology, Boston,, United States
3 Boston University, Public Health, Boston,, United States
§ Presenting author email: jason.bendezu@bmc.org
Electronic publication date: 2012 Oct 22
Publication date: 2012
Volume: 15
Issue: Suppl 3
Electronic Location ID: 18442-012
Copyright: © 2012 J. Bendezu et al.
Copyright year: 2012
License: This is an open-access article distributed under the terms of the Creative Commons Attribution License, which permits unrestricted use, distribution, and reproduction in any medium, provided the original work is properly cited.
License URL: http://creativecommons.org/licenses/by/2.0/

JOURNAL INFORMATION
==============================
NLM Title Abbreviation: J Int AIDS Soc
Journal ID: J Int AIDS Soc
NLM Journal ID: 101478566
Journal ID: JIAS

Journal of the International AIDS Society

EISSN: 1758-2652
Publisher: Wiley

ARTICLE INFORMATION
==============================
Article ID: 18442-013
Subjects: D2 - Behaviourial and social research on risk reduction interventions

WEPDD0303: Street-based adolescents: actual emphasis on HIV prevention

Sakovych O. 1 §
Balakireva O. 2
Bondar T. 2
1 UNICEF Ukraine, Kiev, Ukraine
2 Ukrainian Institute for Social Research after Olexandr Yaremenko, Kiev, Ukraine
§ Presenting author email: osakovych@unicef.org
Electronic publication date: 2012 Oct 22
Publication date: 2012
Volume: 15
Issue: Suppl 3
Electronic Location ID: 18442-013
Copyright: © 2012 O. Sakovych et al.
Copyright year: 2012
License: This is an open-access article distributed under the terms of the Creative Commons Attribution License, which permits unrestricted use, distribution, and reproduction in any medium, provided the original work is properly cited.
License URL: http://creativecommons.org/licenses/by/2.0/

JOURNAL INFORMATION
==============================
NLM Title Abbreviation: J Int AIDS Soc
Journal ID: J Int AIDS Soc
NLM Journal ID: 101478566
Journal ID: JIAS

Journal of the International AIDS Society

EISSN: 1758-2652
Publisher: Wiley

ARTICLE INFORMATION
==============================
Article ID: 18442-014
Subjects: D3 - School-based sexuality education, life skills, gender equality education

THAD0304: Gender and HIV/AIDS education in the multi-cultural context of schools at Kakuma Refugee Camp in Kenya

Ochieng R.M. 1 §
1 Kenyatta University, Educational Foundations, Nairobi, Kenya
§ Presenting author email: rubaimandela@yahoo.com
Electronic publication date: 2012 Oct 22
Publication date: 2012
Volume: 15
Issue: Suppl 3
Electronic Location ID: 18442-014
Copyright: © 2012 R.M. Ochieng et al.
Copyright year: 2012
License: This is an open-access article distributed under the terms of the Creative Commons Attribution License, which permits unrestricted use, distribution, and reproduction in any medium, provided the original work is properly cited.
License URL: http://creativecommons.org/licenses/by/2.0/

JOURNAL INFORMATION
==============================
NLM Title Abbreviation: J Int AIDS Soc
Journal ID: J Int AIDS Soc
NLM Journal ID: 101478566
Journal ID: JIAS

Journal of the International AIDS Society

EISSN: 1758-2652
Publisher: Wiley

ARTICLE INFORMATION
==============================
Article ID: 18442-015
Subjects: D3 - School-based sexuality education, life skills, gender equality education

THAD0305: Uncomfortable silences: narratives of four educators teaching about HIV/AIDS in a high school near Montréal

Cobbler M.-A. 1 §
1 Concordia University Education, Educational Studies Program, Montreal, Canada
§ Presenting author email: ma.cobbler@gmail.com
Electronic publication date: 2012 Oct 22
Publication date: 2012
Volume: 15
Issue: Suppl 3
Electronic Location ID: 18442-015
Copyright: © 2012 M.-A. Cobbler et al.
Copyright year: 2012
License: This is an open-access article distributed under the terms of the Creative Commons Attribution License, which permits unrestricted use, distribution, and reproduction in any medium, provided the original work is properly cited.
License URL: http://creativecommons.org/licenses/by/2.0/

JOURNAL INFORMATION
==============================
NLM Title Abbreviation: J Int AIDS Soc
Journal ID: J Int AIDS Soc
NLM Journal ID: 101478566
Journal ID: JIAS

Journal of the International AIDS Society

EISSN: 1758-2652
Publisher: Wiley

ARTICLE INFORMATION
==============================
Article ID: 18442-016
Subjects: D3 - School-based sexuality education, life skills, gender equality education

THAD0306: Rethinking the ‘teacher’ in school-based, teacher-led sexuality education programmes in rural and urban Tanzania

Matungwa D.J. 1 §
Chenha C. 2
Kachuchuru J. 2
Visser T. 3
van Reeuwijk M. 3
Maro G. 4
Massawe A. 5
Kalongola I. 6
Francis J. 2
Changalucha J. 2
Mshana G. 2
1 National Institute for Medical Research, 28, United Republic of Tanzania
2 National Institute for Medical Research - Mwanza Centre, Mwanza, United Republic of Tanzania
3 Rutgers World Population Fund, Utrecht, Netherlands
4 African Medical and Research Foundation, Tanzania, Dar es Salaam, United Republic of Tanzania
5 Health Actions Promotion Association, Tanzania, Singida, United Republic of Tanzania
6 Restless Development, Iringa, United Republic of Tanzania
§ Presenting author email: matungwa@gmail.com
Electronic publication date: 2012 Oct 22
Publication date: 2012
Volume: 15
Issue: Suppl 3
Electronic Location ID: 18442-016
Copyright: © 2012 D.J. Matungwa et al.
Copyright year: 2012
License: This is an open-access article distributed under the terms of the Creative Commons Attribution License, which permits unrestricted use, distribution, and reproduction in any medium, provided the original work is properly cited.
License URL: http://creativecommons.org/licenses/by/2.0/

JOURNAL INFORMATION
==============================
NLM Title Abbreviation: J Int AIDS Soc
Journal ID: J Int AIDS Soc
NLM Journal ID: 101478566
Journal ID: JIAS

Journal of the International AIDS Society

EISSN: 1758-2652
Publisher: Wiley

ARTICLE INFORMATION
==============================
Article ID: 18442-017
Subjects: D4 - Community, social and political mobilization and building of social capital

THAD0303: Promoting sexual and reproductive health (SRH) in adolescent girls through traditional initiation in the coast region of Tanzania

Itaka A. 1 §
Makokha M. 1
1 FHI 360, UJANA Project, Dar es Salaam, United Republic of Tanzania
§ Presenting author email: aitaka@fhi360.org
Electronic publication date: 2012 Oct 22
Publication date: 2012
Volume: 15
Issue: Suppl 3
Electronic Location ID: 18442-017
Copyright: © 2012 A. Itaka et al.
Copyright year: 2012
License: This is an open-access article distributed under the terms of the Creative Commons Attribution License, which permits unrestricted use, distribution, and reproduction in any medium, provided the original work is properly cited.
License URL: http://creativecommons.org/licenses/by/2.0/

JOURNAL INFORMATION
==============================
NLM Title Abbreviation: J Int AIDS Soc
Journal ID: J Int AIDS Soc
NLM Journal ID: 101478566
Journal ID: JIAS

Journal of the International AIDS Society

EISSN: 1758-2652
Publisher: Wiley

ARTICLE INFORMATION
==============================
Article ID: 18442-018
Subjects: D4 - Community, social and political mobilization and building of social capital

TUPDE0105: Social capital and AIDS competent communities: evidence from eastern Zimbabwe

Campbell C. 1
Nhamo M. 1
Nyamukapa C. 2 3
Madanhire C. 2
Scott K. 4
Skovdal M. 5
Sherr L. 6 §
Gregson S. 2 3
1 London School of Economics and Political Science, London, United Kingdom
2 Biomedical Research and Training Institute, Harare, Zimbabwe
3 Imperial College London, School of Public Healh, London, United Kingdom
4 Johns Hopkins Bloomberg School of Public Health, Baltimore, United States
5 University of Bergen, Department of Health Promotion and Development, Bergen, Norway
6 University College London, Department of Infection and Population Health, London, United Kingdom
§ Presenting author email: l.sherr@ucl.ac.uk
Electronic publication date: 2012 Oct 22
Publication date: 2012
Volume: 15
Issue: Suppl 3
Electronic Location ID: 18442-018
Copyright: © 2012 L. Sherr et al.
Copyright year: 2012
License: This is an open-access article distributed under the terms of the Creative Commons Attribution License, which permits unrestricted use, distribution, and reproduction in any medium, provided the original work is properly cited.
License URL: http://creativecommons.org/licenses/by/2.0/

JOURNAL INFORMATION
==============================
NLM Title Abbreviation: J Int AIDS Soc
Journal ID: J Int AIDS Soc
NLM Journal ID: 101478566
Journal ID: JIAS

Journal of the International AIDS Society

EISSN: 1758-2652
Publisher: Wiley

ARTICLE INFORMATION
==============================
Article ID: 18442-019
Subjects: D4 - Community, social and political mobilization and building of social capital

WEPDD0301: Reducing children's vulnerability in a regions with HIV prevalence with an integrated livelihoods, protection and psychosocial support (PSS) package

Kiiza C. 1
Babu Ndyabahika A. 2 §
1 WEI/Bantana Uganda, Kampala, Uganda
2 Initiative for AIDS Oprhans & Vulnerable Children, Kampala, Uganda
§ Presenting author email: christinebantwana@gmail.com
Electronic publication date: 2012 Oct 22
Publication date: 2012
Volume: 15
Issue: Suppl 3
Electronic Location ID: 18442-019
Copyright: © 2012 A. Babu Ndyabahika et al.
Copyright year: 2012
License: This is an open-access article distributed under the terms of the Creative Commons Attribution License, which permits unrestricted use, distribution, and reproduction in any medium, provided the original work is properly cited.
License URL: http://creativecommons.org/licenses/by/2.0/

JOURNAL INFORMATION
==============================
NLM Title Abbreviation: J Int AIDS Soc
Journal ID: J Int AIDS Soc
NLM Journal ID: 101478566
Journal ID: JIAS

Journal of the International AIDS Society

EISSN: 1758-2652
Publisher: Wiley

ARTICLE INFORMATION
==============================
Article ID: 18442-020
Subjects: D4 - Community, social and political mobilization and building of social capital

THPDC0206: Increasing transgender community capacity to impact HIV prevention and health care services: Coalitions in Action for Transgender Community Health (CATCH)

Castro D. 1 2 3 §
Keatley J. 1 2 3
Gutierrez-Mock L. 1 2
Sevelius J. 1 2
Rebchook G. 1 2
1 Center of Excellence for Transgender Health: University of California, San Francisco, United States
2 Center for AIDS Prevention Studies: University of California, San Francisco, United States
3 Pacific AIDS Education and Training Center: University of California, San Francisco, United States
§ Presenting author email: danielle.castro@ucsf.edu
Electronic publication date: 2012 Oct 22
Publication date: 2012
Volume: 15
Issue: Suppl 3
Electronic Location ID: 18442-020
Copyright: © 2012 D. Castro et al.
Copyright year: 2012
License: This is an open-access article distributed under the terms of the Creative Commons Attribution License, which permits unrestricted use, distribution, and reproduction in any medium, provided the original work is properly cited.
License URL: http://creativecommons.org/licenses/by/2.0/

JOURNAL INFORMATION
==============================
NLM Title Abbreviation: J Int AIDS Soc
Journal ID: J Int AIDS Soc
NLM Journal ID: 101478566
Journal ID: JIAS

Journal of the International AIDS Society

EISSN: 1758-2652
Publisher: Wiley

ARTICLE INFORMATION
==============================
Article ID: 18442-021
Subjects: D7 - Child care, infant feeding, pre-chewing of food (premastication)

MOPDD0303: Determinants of infant feeding intent and appropriateness of choices for formula feeding in the Djoungolo Prevention of Mother-To-Child Transmission of HIV programme, Yaounde, Cameroon

Njom Nlend A.E. 1 §
Bagfegue Ekani B. 2
Tchouamo A. 2
Mbi A. 3
the Mother & Child Djoungolo Network
1 National Social Insurance Fund Hospital, Pediatrics, Yaounde, Cameroon
2 National Social Insurance Fund Hospital, Yaounde, Cameroon
3 Association Camerounaise d'Aide aux Personnes et Familles Affectées par le SIDA, Yaounde,, Cameroon
§ Presenting author email: anne.njom@gmail.com
Electronic publication date: 2012 Oct 22
Publication date: 2012
Volume: 15
Issue: Suppl 3
Electronic Location ID: 18442-021
Copyright: © 2012 A.E. Njom Nlend et al.
Copyright year: 2012
License: This is an open-access article distributed under the terms of the Creative Commons Attribution License, which permits unrestricted use, distribution, and reproduction in any medium, provided the original work is properly cited.
License URL: http://creativecommons.org/licenses/by/2.0/

JOURNAL INFORMATION
==============================
NLM Title Abbreviation: J Int AIDS Soc
Journal ID: J Int AIDS Soc
NLM Journal ID: 101478566
Journal ID: JIAS

Journal of the International AIDS Society

EISSN: 1758-2652
Publisher: Wiley

ARTICLE INFORMATION
==============================
Article ID: 18442-022
Subjects: D8 - Prevention with HIV-positive people

WEAD0305: Effect of a values-based prevention curriculum on HIV-positive couples from four regions in Ethiopia

Brewster-Lee D. 1
Suba M. 2 §
1 Catholic Relief Services, Baltimore, United States
2 Catholic Relief Services Ethiopia, Addis Ababa, Ethiopia
§ Presenting author email: misgina.suba@crs.org
Electronic publication date: 2012 Oct 22
Publication date: 2012
Volume: 15
Issue: Suppl 3
Electronic Location ID: 18442-022
Copyright: © 2012 M. Suba et al.
Copyright year: 2012
License: This is an open-access article distributed under the terms of the Creative Commons Attribution License, which permits unrestricted use, distribution, and reproduction in any medium, provided the original work is properly cited.
License URL: http://creativecommons.org/licenses/by/2.0/

JOURNAL INFORMATION
==============================
NLM Title Abbreviation: J Int AIDS Soc
Journal ID: J Int AIDS Soc
NLM Journal ID: 101478566
Journal ID: JIAS

Journal of the International AIDS Society

EISSN: 1758-2652
Publisher: Wiley

ARTICLE INFORMATION
==============================
Article ID: 18442-023
Subjects: D9 - Counseling and testing (HIV counseling and testing (HCT) and voluntary counseling and testing (VCT)), social, psychological and behavioral aspects of HIV testing and counseling

WEPDE0202: Mapping spatial barriers and facilitators to HIV testing by work environments among sex workers in Vancouver, Canada

Deering K. 1 §
Feng C. 2
Amram O. 3
Montaner J. 1
Chettiar J. 2
Strathdee S. 4
Shannon K. 1
1 University of British Columbia, Medicine, Division of AIDS, Vancouver, Canada
2 BC Centre for Excellence in HIV/AIDS, Vancouver, Canada
3 Simon Fraser University, Geography, Vancouver, Canada
4 University of California – San Diego (UCSD), San Diego, United States
§ Presenting author email: kdeering@cfenet.ubc.ca
Electronic publication date: 2012 Oct 22
Publication date: 2012
Volume: 15
Issue: Suppl 3
Electronic Location ID: 18442-023
Copyright: © 2012 K. Deering et al.
Copyright year: 2012
License: This is an open-access article distributed under the terms of the Creative Commons Attribution License, which permits unrestricted use, distribution, and reproduction in any medium, provided the original work is properly cited.
License URL: http://creativecommons.org/licenses/by/2.0/

JOURNAL INFORMATION
==============================
NLM Title Abbreviation: J Int AIDS Soc
Journal ID: J Int AIDS Soc
NLM Journal ID: 101478566
Journal ID: JIAS

Journal of the International AIDS Society

EISSN: 1758-2652
Publisher: Wiley

ARTICLE INFORMATION
==============================
Article ID: 18442-024
Subjects: D10 - Other behavioural, social and structural interventions, including in the context of biomedical interventions

WEAD0505: Feasibility, acceptability and initial efficacy of the ‘Unity workshop’: an internalized stigma reduction intervention for African American women living with HIV

Rao D. 1 §
Desmond M. 2
Andrasik M. 1
Rasberry T. 3
Lambert N. 4
Cohn S. 4
Simoni J. 1
1 University of Washington, Seattle, United States
2 PATH, Seattle, United States
3 Babes Network YWCA, Seattle, United States
4 Northwestern University, Chicago, United States
§ Presenting author email: deeparao@uw.edu
Electronic publication date: 2012 Oct 22
Publication date: 2012
Volume: 15
Issue: Suppl 3
Electronic Location ID: 18442-024
Copyright: © 2012 D. Rao et al.
Copyright year: 2012
License: This is an open-access article distributed under the terms of the Creative Commons Attribution License, which permits unrestricted use, distribution, and reproduction in any medium, provided the original work is properly cited.
License URL: http://creativecommons.org/licenses/by/2.0/

JOURNAL INFORMATION
==============================
NLM Title Abbreviation: J Int AIDS Soc
Journal ID: J Int AIDS Soc
NLM Journal ID: 101478566
Journal ID: JIAS

Journal of the International AIDS Society

EISSN: 1758-2652
Publisher: Wiley

ARTICLE INFORMATION
==============================
Article ID: 18442-025
Subjects: D10 - Other behavioural, social and structural interventions, including in the context of biomedical interventions

MOPDD0105: Effectiveness of psycho-education in a family-to-family program on family relationships and emotional quotient of adolescents in HIV families in Thailand

Chaitha W. 1
Jiraphongsa C. 2
Khumthong S. 2
Lili L. 3
Sung-Jae L. 3
Isaranun W. 4
Kaeworasan S. 5
Pibulniyom R. 6
Chaitha I. 1
1 Chiangsaen Hospital, Ministry of Public Health, Chiangsaen, Thailand
2 Ministry of Public Health, Department of Disease Control, Bangkok, Thailand
3 University of California, Los Angles, Department of Psychiatry and Biobehavioral Sciences, Los Angles, United States
4 Maechun Hospital, Ministry of Public Health, Maechun, Thailand
5 Khonburi Hospital, Ministry of Public Health, Khonburi, Thailand
6 Parkchong Hospital, Minitry of Public Health, Parkchong, Thailand
W. Chaitha,
Electronic publication date: 2012 Oct 22
Publication date: 2012
Volume: 15
Issue: Suppl 3
Electronic Location ID: 18442-025
Copyright: © 2012 W. Chaitha et al.
Copyright year: 2012
License: This is an open-access article distributed under the terms of the Creative Commons Attribution License, which permits unrestricted use, distribution, and reproduction in any medium, provided the original work is properly cited.
License URL: http://creativecommons.org/licenses/by/2.0/

JOURNAL INFORMATION
==============================
NLM Title Abbreviation: J Int AIDS Soc
Journal ID: J Int AIDS Soc
NLM Journal ID: 101478566
Journal ID: JIAS

Journal of the International AIDS Society

EISSN: 1758-2652
Publisher: Wiley

ARTICLE INFORMATION
==============================
Article ID: 18442-026
Subjects: D10 - Other behavioural, social and structural interventions, including in the context of biomedical interventions

WEPDD0102: Home-based mental health services are associated with improved mental health outcomes among individuals with HIV

Reif S. 1 §
Whetten K. 2
Wilson E. 2
Legrand S. 2
1 Duke University, Center for Health Policy and Inequalities Research, Charlotte, United States
2 Duke University, Center for Health Policy and Inequalities Research, Durham, United States
§ Presenting author email: susan.reif@duke.edu
Electronic publication date: 2012 Oct 22
Publication date: 2012
Volume: 15
Issue: Suppl 3
Electronic Location ID: 18442-026
Copyright: © 2012 S. Reif et al.
Copyright year: 2012
License: This is an open-access article distributed under the terms of the Creative Commons Attribution License, which permits unrestricted use, distribution, and reproduction in any medium, provided the original work is properly cited.
License URL: http://creativecommons.org/licenses/by/2.0/

JOURNAL INFORMATION
==============================
NLM Title Abbreviation: J Int AIDS Soc
Journal ID: J Int AIDS Soc
NLM Journal ID: 101478566
Journal ID: JIAS

Journal of the International AIDS Society

EISSN: 1758-2652
Publisher: Wiley

ARTICLE INFORMATION
==============================
Article ID: 18442-027
Subjects: D12 - Socio-economic vulnerability and stratification (e.g., inequality, poverty, wealth, social status)

WEAD0102: Critical consciousness, perceived racial discrimination and perceived gender discrimination in relation to demographics and HIV status in African American women

Kelso G. 1 §
Cruise R. 1
Dale S. 1
Weber K. 2
Cohen M. 2
Brody L. 1
1 Boston University, Psychology, Boston, United States
2 Cook County Health & Hospital Systems, Chicago, United States
§ Presenting author email: gkelso@bu.edu
Electronic publication date: 2012 Oct 22
Publication date: 2012
Volume: 15
Issue: Suppl 3
Electronic Location ID: 18442-027
Copyright: © 2012 G. Kelso et al.
Copyright year: 2012
License: This is an open-access article distributed under the terms of the Creative Commons Attribution License, which permits unrestricted use, distribution, and reproduction in any medium, provided the original work is properly cited.
License URL: http://creativecommons.org/licenses/by/2.0/

JOURNAL INFORMATION
==============================
NLM Title Abbreviation: J Int AIDS Soc
Journal ID: J Int AIDS Soc
NLM Journal ID: 101478566
Journal ID: JIAS

Journal of the International AIDS Society

EISSN: 1758-2652
Publisher: Wiley

ARTICLE INFORMATION
==============================
Article ID: 18442-028
Subjects: D12 - Socio-economic vulnerability and stratification (e.g., inequality, poverty, wealth, social status)

FRLBD02: Freedom to adhere: the complex relationship between democracy, wealth disparity, social capital and HIV medication adherence in adults living with HIV

Phillips J.C. 1 §
Webel A. 2
Dawson Rose C. 3
Holzemer W.L. 4
Chen W.-T. 5
Johnson M.O. 6
Kirksey K. 7
Voss J. 8
Sefcik E. 9
Eller L.S. 4
Corless I.B. 10
Wantland D. 4
Portillo C. 3
Tyer-Viola L. 10
Sullivan K.M. 11
Nicholas P.K. 10
Iipinge S. 12
Nokes K. 13
Kemppainen J. 14
Rivero-Mendez M. 15
Chaiphibalsarisdi P. 16
Reid P. 14
Brion J. 17
1 University of Ottawa, Faculty of Health Sciences, School of Nursing, Ottawa, Canada
2 Case Western Reserve University, Frances Payne Bolton School of Nursing, Cleveland, United States
3 University of California, San Francisco, School of Nursing, San Francisco, United States
4 Rutgers University, College of Nursing, Newark, United States
5 Yale University, New Haven, United States
6 University of California, San Francisco, San Francisco, United States
7 Seton Family of Hospitals, Center for Nursing Research, Austin, United States
8 University of Washington, Seattle, United States
9 Texas A&M University-Corpus Christi, Corpus Christi, United States
0 MGH Institute of Health Professions, Boston, United States
1 University of Hawaii at Manoa, Honolulu, United States
2 University of Namibia, Windhoek, Namibia
3 Hunter College, CUNY, Hunter Bellevue School of Nursing, New York, United States
4 University of North Carolina - Wilmington, Wilmington, United States
5 University of Puerto Rico, San Juan, Puerto Rico
6 Suan Sunandha Rajabhat University, Bangkok, Thailand
7 Duke University, School of Nursing, Durham, United States
§ Presenting author email: jcraigarnp@gmail.com
Electronic publication date: 2012 Oct 22
Publication date: 2012
Volume: 15
Issue: Suppl 3
Electronic Location ID: 18442-028
Copyright: © 2012 J.C. Phillips et al.
Copyright year: 2012
License: This is an open-access article distributed under the terms of the Creative Commons Attribution License, which permits unrestricted use, distribution, and reproduction in any medium, provided the original work is properly cited.
License URL: http://creativecommons.org/licenses/by/2.0/

JOURNAL INFORMATION
==============================
NLM Title Abbreviation: J Int AIDS Soc
Journal ID: J Int AIDS Soc
NLM Journal ID: 101478566
Journal ID: JIAS

Journal of the International AIDS Society

EISSN: 1758-2652
Publisher: Wiley

ARTICLE INFORMATION
==============================
Article ID: 18442-029
Subjects: D12 - Socio-economic vulnerability and stratification (e.g., inequality, poverty, wealth, social status)

WEPDD0106: When time doesn't heal: complicated grief among orphans in rural Zambia

Gschwend A. 1 2
Wespi K. 1
Amman P. 1
Langhaug L. 3 §
1 Swiss Academy for Development, Biel-Bienne, Switzerland
2 University of Bern, Institute of Psychology, Section of Social Pscychology, Bern, Switzerland
3 REPSSI, Harare, Zimbabwe
§ Presenting author email: lisa.langhaug@gmail.com
Electronic publication date: 2012 Oct 22
Publication date: 2012
Volume: 15
Issue: Suppl 3
Electronic Location ID: 18442-029
Copyright: © 2012 L. Langhaug et al.
Copyright year: 2012
License: This is an open-access article distributed under the terms of the Creative Commons Attribution License, which permits unrestricted use, distribution, and reproduction in any medium, provided the original work is properly cited.
License URL: http://creativecommons.org/licenses/by/2.0/

JOURNAL INFORMATION
==============================
NLM Title Abbreviation: J Int AIDS Soc
Journal ID: J Int AIDS Soc
NLM Journal ID: 101478566
Journal ID: JIAS

Journal of the International AIDS Society

EISSN: 1758-2652
Publisher: Wiley

ARTICLE INFORMATION
==============================
Article ID: 18442-030
Subjects: D12 - Socio-economic vulnerability and stratification (e.g., inequality, poverty, wealth, social status)

WEPDD0304: Community-level income inequality and HIV prevalence in injecting drug users in Thai Nguyen, Viet Nam

Lim T. 1 §
Go V. 1
Ha T.V. 2
Minh N.L. 3
Viet Anh C. 2
Davis W. 4
Quan V.M. 2
1 Johns Hopkins Bloomberg School of Public Health, International Health, Baltimore, United States
2 Johns Hopkins Bloomberg School of Public Health, Department of Epidemiology, Hanoi, Viet Nam
3 Center for Preventive Medicine, Thai Nguyen, Viet Nam
4 Johns Hopkins Bloomberg School of Public Health, Department of Epidemiology, Baltimore, United States
§ Presenting author email: travis.lim@mail.mcgill.ca
Electronic publication date: 2012 Oct 22
Publication date: 2012
Volume: 15
Issue: Suppl 3
Electronic Location ID: 18442-030
Copyright: © 2012 T. Lim et al.
Copyright year: 2012
License: This is an open-access article distributed under the terms of the Creative Commons Attribution License, which permits unrestricted use, distribution, and reproduction in any medium, provided the original work is properly cited.
License URL: http://creativecommons.org/licenses/by/2.0/

JOURNAL INFORMATION
==============================
NLM Title Abbreviation: J Int AIDS Soc
Journal ID: J Int AIDS Soc
NLM Journal ID: 101478566
Journal ID: JIAS

Journal of the International AIDS Society

EISSN: 1758-2652
Publisher: Wiley

ARTICLE INFORMATION
==============================
Article ID: 18442-031
Subjects: D12 - Socio-economic vulnerability and stratification (e.g., inequality, poverty, wealth, social status)

WEPDD0305: Does the ‘inverse equity hypothesis’ explain how both poverty and wealth can be associated with HIV prevalence in sub-Saharan Africa?

Hargreaves J. 1 2 §
Davey C. 1
White R. 1
1 London School of Hygiene and Tropical Medicine, Department of Infectious Disease Epidemiology, London, United Kingdom
2 Chatham House Centre on Global Health Security, London, United Kingdom
§ Presenting author email: james.hargreaves@lshtm.ac.uk
Electronic publication date: 2012 Oct 22
Publication date: 2012
Volume: 15
Issue: Suppl 3
Electronic Location ID: 18442-031
Copyright: © 2012 J. Hargreaves et al.
Copyright year: 2012
License: This is an open-access article distributed under the terms of the Creative Commons Attribution License, which permits unrestricted use, distribution, and reproduction in any medium, provided the original work is properly cited.
License URL: http://creativecommons.org/licenses/by/2.0/

JOURNAL INFORMATION
==============================
NLM Title Abbreviation: J Int AIDS Soc
Journal ID: J Int AIDS Soc
NLM Journal ID: 101478566
Journal ID: JIAS

Journal of the International AIDS Society

EISSN: 1758-2652
Publisher: Wiley

ARTICLE INFORMATION
==============================
Article ID: 18442-032
Subjects: D12 - Socio-economic vulnerability and stratification (e.g., inequality, poverty, wealth, social status)

WEPDD0306: ‘There is hunger in my community': food security as a cyclically driving force in sex work in Swaziland

Fielding-Miller R. 1 §
Mnisi Z. 2
Dlamini N. 3
Baral S. 4
Kennedy C. 4
1 Emory University Rollins School of Public Health, Behavioral Sciences and Health Education, Atlanta, United States
2 Swaziland Ministry of Health and Social Welfare, SNAP, Mbabane, Swaziland
3 Swaziland Ministry of Health and Social Welfare, Mbabane, Swaziland
4 Johns Hopkins Bloomberg School of Public Health, Baltimore, United States
§ Presenting author email: rfieldi@emory.edu
Electronic publication date: 2012 Oct 22
Publication date: 2012
Volume: 15
Issue: Suppl 3
Electronic Location ID: 18442-032
Copyright: © 2012 R. Fielding-Miller et al.
Copyright year: 2012
License: This is an open-access article distributed under the terms of the Creative Commons Attribution License, which permits unrestricted use, distribution, and reproduction in any medium, provided the original work is properly cited.
License URL: http://creativecommons.org/licenses/by/2.0/

JOURNAL INFORMATION
==============================
NLM Title Abbreviation: J Int AIDS Soc
Journal ID: J Int AIDS Soc
NLM Journal ID: 101478566
Journal ID: JIAS

Journal of the International AIDS Society

EISSN: 1758-2652
Publisher: Wiley

ARTICLE INFORMATION
==============================
Article ID: 18442-033
Subjects: D13 - Migration, social movements and population dislocation: mobile and immigrant populations (including people living with HIV)

THPDD0104: Increasing HIV testing among African refugees in Africa: intervening in the daily survival cycle to encourage priority shifting

O'Laughlin K.N. 1 2 §
Faustin Z.M. 3
Rouhani S.A. 1 2 4
Ware N.C. 2 5
1 Brigham & Women's Hospital, Emergency Medicine, Boston, United States
2 Harvard Medical School, Boston, United States
3 Bugema University, Kampala, Uganda
4 Massachusetts General Hospital, Boston, United States
5 Brigham & Women's Hospital, Boston, United States
§ Presenting author email: kolaughlin@partners.org
Electronic publication date: 2012 Oct 22
Publication date: 2012
Volume: 15
Issue: Suppl 3
Electronic Location ID: 18442-033
Copyright: © 2012 K.N. O'Laughlin et al.
Copyright year: 2012
License: This is an open-access article distributed under the terms of the Creative Commons Attribution License, which permits unrestricted use, distribution, and reproduction in any medium, provided the original work is properly cited.
License URL: http://creativecommons.org/licenses/by/2.0/

JOURNAL INFORMATION
==============================
NLM Title Abbreviation: J Int AIDS Soc
Journal ID: J Int AIDS Soc
NLM Journal ID: 101478566
Journal ID: JIAS

Journal of the International AIDS Society

EISSN: 1758-2652
Publisher: Wiley

ARTICLE INFORMATION
==============================
Article ID: 18442-034
Subjects: D14 - Family structures, kinship, and social safety nets for widows, orphans and other vulnerable groups

MOAD0305: The psychosocial impact of HIV on the siblings of infected children

Marukutira T. 1 2
Letamo G. 3
Mabikwa V. 1 2
Karugaba G. 1 2
Makhanda J. 1 2
Marape M. 1 2 4
Seleke R. 1 2
Anabwani G.M. 1 2 4 §
1 Botswana-Baylor Children's Clinical Center of Excellence, Gaborone, Botswana
2 Baylor International Pediatric AIDS Initiative, Baylor College of Medicine, Pediatric Retrovirology, Houston, United States
3 University of Botswana, Gaborone, Botswana
4 Texas Children's Hospital, Pediatrics, Houston, United States
§ Presenting author email: ganabwani@baylorbotswana.org.bw
Electronic publication date: 2012 Oct 22
Publication date: 2012
Volume: 15
Issue: Suppl 3
Electronic Location ID: 18442-034
Copyright: © 2012 G.M. Anabwani et al.
Copyright year: 2012
License: This is an open-access article distributed under the terms of the Creative Commons Attribution License, which permits unrestricted use, distribution, and reproduction in any medium, provided the original work is properly cited.
License URL: http://creativecommons.org/licenses/by/2.0/

JOURNAL INFORMATION
==============================
NLM Title Abbreviation: J Int AIDS Soc
Journal ID: J Int AIDS Soc
NLM Journal ID: 101478566
Journal ID: JIAS

Journal of the International AIDS Society

EISSN: 1758-2652
Publisher: Wiley

ARTICLE INFORMATION
==============================
Article ID: 18442-035
Subjects: D14 - Family structures, kinship, and social safety nets for widows, orphans and other vulnerable groups

WEPDD0104: Care and support by households and extended families in the era of HIV treatment: responses to HIV and AIDS in rural South Africa

Knight L. 1 §
Hosegood V. 2 3
Timaeus I. 4
1 Human Sciences Research Council, HIV/AIDS, STIs and TB Unit, Durban, South Africa
2 University of Southampton, Social Sciences: Social Statistics & Demography, Southampton, United Kingdom
3 Africa Centre for Health and Population Studies, Mtubatuba, South Africa
4 London School of Hygiene and Tropical Medicine, Department of Population Studies, London, United Kingdom
§ Presenting author email: lknight@hsrc.ac.za
Electronic publication date: 2012 Oct 22
Publication date: 2012
Volume: 15
Issue: Suppl 3
Electronic Location ID: 18442-035
Copyright: © 2012 L. Knight et al.
Copyright year: 2012
License: This is an open-access article distributed under the terms of the Creative Commons Attribution License, which permits unrestricted use, distribution, and reproduction in any medium, provided the original work is properly cited.
License URL: http://creativecommons.org/licenses/by/2.0/

JOURNAL INFORMATION
==============================
NLM Title Abbreviation: J Int AIDS Soc
Journal ID: J Int AIDS Soc
NLM Journal ID: 101478566
Journal ID: JIAS

Journal of the International AIDS Society

EISSN: 1758-2652
Publisher: Wiley

ARTICLE INFORMATION
==============================
Article ID: 18442-036
Subjects: D14 - Family structures, kinship, and social safety nets for widows, orphans and other vulnerable groups

WEPDD0105: Family network proportion and HIV risk among black men who have sex with men

Schneider J.A. 1 2
Michaels S. 3
Bouris A. 4
1 University of Chicago, Medicine, Chicago, United States
2 University of Chicago, Health Studies, Chicago, United States
3 National Opinion Research Center, Chicago, United States
4 University of Chicago, Social Services Administration, Chicago, United States
J.A. Schneider,
Electronic publication date: 2012 Oct 22
Publication date: 2012
Volume: 15
Issue: Suppl 3
Electronic Location ID: 18442-036
Copyright: © 2012 J.A. Schneider et al.
Copyright year: 2012
License: This is an open-access article distributed under the terms of the Creative Commons Attribution License, which permits unrestricted use, distribution, and reproduction in any medium, provided the original work is properly cited.
License URL: http://creativecommons.org/licenses/by/2.0/

JOURNAL INFORMATION
==============================
NLM Title Abbreviation: J Int AIDS Soc
Journal ID: J Int AIDS Soc
NLM Journal ID: 101478566
Journal ID: JIAS

Journal of the International AIDS Society

EISSN: 1758-2652
Publisher: Wiley

ARTICLE INFORMATION
==============================
Article ID: 18442-037
Subjects: D15 - Violence and conflict: political, gender, social, structural, interpersonal and family-based

WEAD0103: Gender disparities and inequitable gender norms: implications for HIV prevention programming in Zambia

Tun W. 1 §
Keesbury J. 2
Simmonds F.N. 3
Sheehy M. 4
Moyo T. 3
Rathner C. 5
Kalibala S. 1
1 Population Council, HIV and AIDS Program, Washington, United States
2 PATH, Washington, United States
3 Population Council, Zambia, Lusaka, Zambia
4 Population Council, HIV and AIDS Program, New York, United States
5 FHI 360 Zambia, Lusaka, Zambia
§ Presenting author email: wtun@popcouncil.org
Electronic publication date: 2012 Oct 22
Publication date: 2012
Volume: 15
Issue: Suppl 3
Electronic Location ID: 18442-037
Copyright: © 2012 W. Tun et al.
Copyright year: 2012
License: This is an open-access article distributed under the terms of the Creative Commons Attribution License, which permits unrestricted use, distribution, and reproduction in any medium, provided the original work is properly cited.
License URL: http://creativecommons.org/licenses/by/2.0/

JOURNAL INFORMATION
==============================
NLM Title Abbreviation: J Int AIDS Soc
Journal ID: J Int AIDS Soc
NLM Journal ID: 101478566
Journal ID: JIAS

Journal of the International AIDS Society

EISSN: 1758-2652
Publisher: Wiley

ARTICLE INFORMATION
==============================
Article ID: 18442-038
Subjects: D15 - Violence and conflict: political, gender, social, structural, interpersonal and family-based

WEAD0104: Abuse and mortality in women with and at risk for HIV

Weber K. 1 §
Cole S. 2
Agniel D. 1
Schwartz R. 3
Anastos K. 4
Burke-Miller J. 1
Young M. 5
Golub E. 6
Cohen M. 1 7
1 Cook County Health & Hospital Systems, The CORE Center, Chicago, United States
2 University of North Carolina-Chapel Hill, Epidemiology, Chapel Hill, United States
3 SUNY Downstate Medical Center, Preventative Medicine and Community Health, Brooklyn, United States
4 Montefiore Medical Center & Albert Einstein College of Medicine, Department of Medicine, Bronx, United States
5 Georgetown University Medical Center, Department of Medicine, Washington, United States
6 Johns Hopkins School of Public Health, Baltimore, United States
7 Rush University Medical Center, Department of Medicine, Chicago, United States
§ Presenting author email: weberkathleen@ameritech.net
Electronic publication date: 2012 Oct 22
Publication date: 2012
Volume: 15
Issue: Suppl 3
Electronic Location ID: 18442-038
Copyright: © 2012 K. Weber et al.
Copyright year: 2012
License: This is an open-access article distributed under the terms of the Creative Commons Attribution License, which permits unrestricted use, distribution, and reproduction in any medium, provided the original work is properly cited.
License URL: http://creativecommons.org/licenses/by/2.0/

JOURNAL INFORMATION
==============================
NLM Title Abbreviation: J Int AIDS Soc
Journal ID: J Int AIDS Soc
NLM Journal ID: 101478566
Journal ID: JIAS

Journal of the International AIDS Society

EISSN: 1758-2652
Publisher: Wiley

ARTICLE INFORMATION
==============================
Article ID: 18442-039
Subjects: D15 - Violence and conflict: political, gender, social, structural, interpersonal and family-based

WEAD0106: HIV risk behaviour among victims and perpetrators of male-on-male sexual violence in South Africa: results from a population-based survey

Dunkle K. 1 §
Jewkes R. 2
Murdock D. 1
Sikweyiya Y. 2
Morrell R. 3
1 Emory University, Behavioral Sciences and Health Education, Atlanta, United States
2 Medical Research Council of South Africa, Gender and Health Research Unit, Pretoria, South Africa
3 University of Cape Town, Programme for the Enhancement of Research Capacity, Cape Town, South Africa
§ Presenting author email: kdunkle@emory.edu
Electronic publication date: 2012 Oct 22
Publication date: 2012
Volume: 15
Issue: Suppl 3
Electronic Location ID: 18442-039
Copyright: © 2012 K. Dunkle et al.
Copyright year: 2012
License: This is an open-access article distributed under the terms of the Creative Commons Attribution License, which permits unrestricted use, distribution, and reproduction in any medium, provided the original work is properly cited.
License URL: http://creativecommons.org/licenses/by/2.0/

JOURNAL INFORMATION
==============================
NLM Title Abbreviation: J Int AIDS Soc
Journal ID: J Int AIDS Soc
NLM Journal ID: 101478566
Journal ID: JIAS

Journal of the International AIDS Society

EISSN: 1758-2652
Publisher: Wiley

ARTICLE INFORMATION
==============================
Article ID: 18442-040
Subjects: D15 - Violence and conflict: political, gender, social, structural, interpersonal and family-based

MOPDD0205: Alarming rates of occupational violence and associated HIV risks among young female sex workers in post-conflict northern Uganda

Muldoon K. 1 2 §
Akello M. 3
Muzaaya G. 3
Simo A. 1
Shannon K. 1 2
1 BC Centre for Excellence in HIV/AIDS, Vancouver, Canada
2 University of British Columbia, Vancouver, Canada
3 The AIDS Support Organization, Gulu, Uganda
§ Presenting author email: katherine.muldoon@gmail.com
Electronic publication date: 2012 Oct 22
Publication date: 2012
Volume: 15
Issue: Suppl 3
Electronic Location ID: 18442-040
Copyright: © 2012 K. Muldoon et al.
Copyright year: 2012
License: This is an open-access article distributed under the terms of the Creative Commons Attribution License, which permits unrestricted use, distribution, and reproduction in any medium, provided the original work is properly cited.
License URL: http://creativecommons.org/licenses/by/2.0/

JOURNAL INFORMATION
==============================
NLM Title Abbreviation: J Int AIDS Soc
Journal ID: J Int AIDS Soc
NLM Journal ID: 101478566
Journal ID: JIAS

Journal of the International AIDS Society

EISSN: 1758-2652
Publisher: Wiley

ARTICLE INFORMATION
==============================
Article ID: 18442-041
Subjects: D16 - HIV-related stigma, layered stigmas and marginalized identities

WEAD0501: Success! Interventions that work to reduce HIV stigma and discrimination in communities: results of an evaluation study in Thailand

Jain A. 1 §
Nuankaew R. 2
Oranop na Ayuthaya P. 3
Richter K. 4
1 Johns Hopkins University, Baltimore, United States
2 Population and Community Development Association, Bangkok, Thailand
3 PACT Thailand, Bangkok, Thailand
4 Mahidol University, Institute for Population and Social Research, Nakhon Pathom, Thailand
§ Presenting author email: aparna727@gmail.com
Electronic publication date: 2012 Oct 22
Publication date: 2012
Volume: 15
Issue: Suppl 3
Electronic Location ID: 18442-041
Copyright: © 2012 A. Jain et al.
Copyright year: 2012
License: This is an open-access article distributed under the terms of the Creative Commons Attribution License, which permits unrestricted use, distribution, and reproduction in any medium, provided the original work is properly cited.
License URL: http://creativecommons.org/licenses/by/2.0/

JOURNAL INFORMATION
==============================
NLM Title Abbreviation: J Int AIDS Soc
Journal ID: J Int AIDS Soc
NLM Journal ID: 101478566
Journal ID: JIAS

Journal of the International AIDS Society

EISSN: 1758-2652
Publisher: Wiley

ARTICLE INFORMATION
==============================
Article ID: 18442-042
Subjects: D16 - HIV-related stigma, layered stigmas and marginalized identities

WEAD0503: Stigma and serostatus disclosure within partnerships in four African countries: a mixed methods approach

Hardon A. 1
Bongololo-Mbera G. 2
Cherutich P. 3
Gomez G.B. 4 §
Kahega E. 1
Ky-Zerbo O. 5
Vernooij E. 1
Wanyenze R. 6
Obermeyer C. 7
1 Amsterdam Institute for Social Science Research, Amsterdam, Netherlands
2 Research for Equity and Community Health Trust, Lilongwe, Malawi
3 National AIDS/STD Control Programme, Ministry of Health, Nairobi, Kenya
4 Amsterdam Institute for Global Health and Development, Amsterdam, Netherlands
5 Programme d'Appui au Monde Associatif & Communautaire de Lutte Contre le VIH/SIDA, Ouagadougou, Burkina Faso
6 Makerere University School of Public Health, Kampala, Uganda
7 American University of Beirut, Faculty of Health Sciences, Beirut, Lebanon
§ Presenting author email: g.gomez@aighd.org
Electronic publication date: 2012 Oct 22
Publication date: 2012
Volume: 15
Issue: Suppl 3
Electronic Location ID: 18442-042
Copyright: © 2012 G.B. Gomez et al.
Copyright year: 2012
License: This is an open-access article distributed under the terms of the Creative Commons Attribution License, which permits unrestricted use, distribution, and reproduction in any medium, provided the original work is properly cited.
License URL: http://creativecommons.org/licenses/by/2.0/

JOURNAL INFORMATION
==============================
NLM Title Abbreviation: J Int AIDS Soc
Journal ID: J Int AIDS Soc
NLM Journal ID: 101478566
Journal ID: JIAS

Journal of the International AIDS Society

EISSN: 1758-2652
Publisher: Wiley

ARTICLE INFORMATION
==============================
Article ID: 18442-043
Subjects: D16 - HIV-related stigma, layered stigmas and marginalized identities

WEAD0504: Internal stigma among HIV-positive adults in Ethiopia

Bezabih T. 1 §
1 World Food Programme, Programme/Nutrition and Education/HIV and AIDS, Addis Ababa, Ethiopia
§ Presenting author email: tsegazeab.bezabih@gmail.com
Electronic publication date: 2012 Oct 22
Publication date: 2012
Volume: 15
Issue: Suppl 3
Electronic Location ID: 18442-043
Copyright: © 2012 T. Bezabih et al.
Copyright year: 2012
License: This is an open-access article distributed under the terms of the Creative Commons Attribution License, which permits unrestricted use, distribution, and reproduction in any medium, provided the original work is properly cited.
License URL: http://creativecommons.org/licenses/by/2.0/

JOURNAL INFORMATION
==============================
NLM Title Abbreviation: J Int AIDS Soc
Journal ID: J Int AIDS Soc
NLM Journal ID: 101478566
Journal ID: JIAS

Journal of the International AIDS Society

EISSN: 1758-2652
Publisher: Wiley

ARTICLE INFORMATION
==============================
Article ID: 18442-044
Subjects: D16 - HIV-related stigma, layered stigmas and marginalized identities

WEPDD0103: Risk and protective factors for depression symptoms among children affected by HIV/AIDS in rural China: a structural equation modeling analysis

Wang B. 1
Li X. 1
Stanton B. 1
Chi P. 1 §
1 Wayne State University, Detroit, United States
§ Presenting author email: pch@med.wayne.edu
Electronic publication date: 2012 Oct 22
Publication date: 2012
Volume: 15
Issue: Suppl 3
Electronic Location ID: 18442-044
Copyright: © 2012 P. Chi et al.
Copyright year: 2012
License: This is an open-access article distributed under the terms of the Creative Commons Attribution License, which permits unrestricted use, distribution, and reproduction in any medium, provided the original work is properly cited.
License URL: http://creativecommons.org/licenses/by/2.0/

JOURNAL INFORMATION
==============================
NLM Title Abbreviation: J Int AIDS Soc
Journal ID: J Int AIDS Soc
NLM Journal ID: 101478566
Journal ID: JIAS

Journal of the International AIDS Society

EISSN: 1758-2652
Publisher: Wiley

ARTICLE INFORMATION
==============================
Article ID: 18442-045
Subjects: D17 - Racism and other forms of social exclusion

WEAD0502: Racism, sexism, HIV-related stigma and quality of life among HIV-positive black African Caribbean women in Ontario, Canada

Logie C. 1 2 §
James L. 3
Tharao W. 3
Loutfy M. 2
1 University of Calgary, Faculty of Social Work, Calgary, Canada
2 University of Toronto, Women's College Research Institute, Toronto, Canada
3 Women's Health in Women's Hands Community Health Centre, Toronto, Canada
§ Presenting author email: logiech@yahoo.com
Electronic publication date: 2012 Oct 22
Publication date: 2012
Volume: 15
Issue: Suppl 3
Electronic Location ID: 18442-045
Copyright: © 2012 C. Logie et al.
Copyright year: 2012
License: This is an open-access article distributed under the terms of the Creative Commons Attribution License, which permits unrestricted use, distribution, and reproduction in any medium, provided the original work is properly cited.
License URL: http://creativecommons.org/licenses/by/2.0/

JOURNAL INFORMATION
==============================
NLM Title Abbreviation: J Int AIDS Soc
Journal ID: J Int AIDS Soc
NLM Journal ID: 101478566
Journal ID: JIAS

Journal of the International AIDS Society

EISSN: 1758-2652
Publisher: Wiley

ARTICLE INFORMATION
==============================
Article ID: 18442-046
Subjects: D21 - Sex and gender

TUAD0301: Condom attitudes of female entertainment workers in Metro Manila, the Philippines: setting, peer influence and social support

Urada L.A. 1 §
Strathdee S.A. 1
Schilling R.F. 2
de Guia B. 3
Morisky D.E. 4
1 University of California at San Diego, School of Medicine/Department of Medicine, Division of Global Public Health, La Jolla, United States
2 University of California at Los Angeles, Luskin School of Public Affairs/Department of Social Welfare, Los Angeles, United States
3 PAMAC-Q (Peer Educator's Movement for Empowerment) and the Philippine Rural Reconstruction Movement, Inc., Quezon City, Philippines
4 University of California at Los Angeles, School of Public Health/Department of Community Health Sciences, Los Angeles, United States
§ Presenting author email: lurada@ucsd.edu
Electronic publication date: 2012 Oct 22
Publication date: 2012
Volume: 15
Issue: Suppl 3
Electronic Location ID: 18442-046
Copyright: © 2012 L.A. Urada et al.
Copyright year: 2012
License: This is an open-access article distributed under the terms of the Creative Commons Attribution License, which permits unrestricted use, distribution, and reproduction in any medium, provided the original work is properly cited.
License URL: http://creativecommons.org/licenses/by/2.0/

JOURNAL INFORMATION
==============================
NLM Title Abbreviation: J Int AIDS Soc
Journal ID: J Int AIDS Soc
NLM Journal ID: 101478566
Journal ID: JIAS

Journal of the International AIDS Society

EISSN: 1758-2652
Publisher: Wiley

ARTICLE INFORMATION
==============================
Article ID: 18442-047
Subjects: D22 - Age

THPDD0201: Getting older while living with HIV in the United States

Nokes K. 1 §
Sefcik E. 2
Johnson M.O. 3
Webel A. 4
Rivero-Méndez M. 5
Voss J. 6
Chen W.-T. 7
Iipinge S. 8
Portillo C.J. 9
Corless I.B. 10
Sullivan K.M. 11
Tyer-Viola L. 10
Kemppainen J. 12
Sanzero Eller L. 13
Dawson Rose C. 9
Phillips J.C. 14
Kirksey K. 15
Nicholas P. 16
Brion J. 17
Wantland D. 18
Holzemer W.L. 18
1 Hunter College, CUNY, Hunter-Bellevue School of Nursing, New York, United States
2 Texas A&M University-Corpus Christi, Nursing, Corpus Christi, United States
3 University of California at San Francisco, San Francisco, United States
4 Case Western Reserve University, Nursing, Cleveland, United States
5 University of Puerto Rico, Nursing, San Juan, Puerto Rico
6 University of Washington, Nursing, Seattle, United States
7 Yale University, Nursing, New Haven, United States
8 University of Namibia, Nursing, Windhoek, Namibia
9 University of California at San Francisco, Nursing, San Francisco, United States
0 MGH Institute of Health Professions, Nursing, Boston, United States
1 University of Hawaii, Nursing, Honolulu, United States
2 University of North Carolina Wilmington, Nursing, Wilmington, United States
3 Rutgers College of Nursing, Nursing, Cedar Grove, United States
4 University of British Columbia, Nursing, Vancouver, Canada
5 Seton Family of Hospitals, Clinical Education Center at Brakenridge, Austin, United States
6 Brigham and Women's Hospital and MGH, Global Health, Boston, United States
7 Duke University, School of Nursing, Durham, United States
8 Rutgers College of Nursing, Nursing, Newark, United States
§ Presenting author email: knokes@hunter.cuny.edu
Electronic publication date: 2012 Oct 22
Publication date: 2012
Volume: 15
Issue: Suppl 3
Electronic Location ID: 18442-047
Copyright: © 2012 K. Nokes et al.
Copyright year: 2012
License: This is an open-access article distributed under the terms of the Creative Commons Attribution License, which permits unrestricted use, distribution, and reproduction in any medium, provided the original work is properly cited.
License URL: http://creativecommons.org/licenses/by/2.0/

JOURNAL INFORMATION
==============================
NLM Title Abbreviation: J Int AIDS Soc
Journal ID: J Int AIDS Soc
NLM Journal ID: 101478566
Journal ID: JIAS

Journal of the International AIDS Society

EISSN: 1758-2652
Publisher: Wiley

ARTICLE INFORMATION
==============================
Article ID: 18442-048
Subjects: D22 - Age

THPDD0205: HIV risk and recent sexual behaviour of older adults in rural South Africa

Williams J. 1 §
Gómez-Olivé F.X. 2
Angotti N. 1
Kabudula C. 2
Menken J. 1
Clark S. 3
Klipstein-Grobusch K. 4
Tollman S. 2
1 University of Colorado, Institute of Behavioral Science, Boulder, United States
2 University of the Witwatersrand (WITS), MRC/Wits Rural Public Health and Health Transitions Research Unit, Johannesburg, South Africa
3 University of Washington, Seattle, United States
4 Utrecht University, Global Health Unit, Utrecht, Netherlands
§ Presenting author email: jillcolo@colorado.edu
Electronic publication date: 2012 Oct 22
Publication date: 2012
Volume: 15
Issue: Suppl 3
Electronic Location ID: 18442-048
Copyright: © 2012 J. Williams et al.
Copyright year: 2012
License: This is an open-access article distributed under the terms of the Creative Commons Attribution License, which permits unrestricted use, distribution, and reproduction in any medium, provided the original work is properly cited.
License URL: http://creativecommons.org/licenses/by/2.0/

JOURNAL INFORMATION
==============================
NLM Title Abbreviation: J Int AIDS Soc
Journal ID: J Int AIDS Soc
NLM Journal ID: 101478566
Journal ID: JIAS

Journal of the International AIDS Society

EISSN: 1758-2652
Publisher: Wiley

ARTICLE INFORMATION
==============================
Article ID: 18442-049
Subjects: D22 - Age

THPDD0207: Determining the healthcare needs of older people living with HIV in Uganda: a qualitative study

Kuteesa M. 1 2 §
1 Uganda Cares-AIDS Healthcare Foundation, Clinical Care, Kampala, Uganda
2 University of Sydney, School of Public Health, Sydney, Australia
§ Presenting author email: monakello@gmail.com
Electronic publication date: 2012 Oct 22
Publication date: 2012
Volume: 15
Issue: Suppl 3
Electronic Location ID: 18442-049
Copyright: © 2012 M. Kuteesa et al.
Copyright year: 2012
License: This is an open-access article distributed under the terms of the Creative Commons Attribution License, which permits unrestricted use, distribution, and reproduction in any medium, provided the original work is properly cited.
License URL: http://creativecommons.org/licenses/by/2.0/

JOURNAL INFORMATION
==============================
NLM Title Abbreviation: J Int AIDS Soc
Journal ID: J Int AIDS Soc
NLM Journal ID: 101478566
Journal ID: JIAS

Journal of the International AIDS Society

EISSN: 1758-2652
Publisher: Wiley

ARTICLE INFORMATION
==============================
Article ID: 18442-050
Subjects: D23 - Incarceration

WEAD0605: Prevalence of transmissible infections and socio-demographic and behavioral risk factors amongst prisoners in Mexico City: a cross-sectional study of 17,296 inmates

Bautista-Arredondo S. 1 §
1 National Institute of Public Health, Cuernavaca, Mexico
§ Presenting author email: sbautista@insp.mx
Electronic publication date: 2012 Oct 22
Publication date: 2012
Volume: 15
Issue: Suppl 3
Electronic Location ID: 18442-050
Copyright: © 2012 S. Bautista-Arredondo et al.
Copyright year: 2012
License: This is an open-access article distributed under the terms of the Creative Commons Attribution License, which permits unrestricted use, distribution, and reproduction in any medium, provided the original work is properly cited.
License URL: http://creativecommons.org/licenses/by/2.0/

JOURNAL INFORMATION
==============================
NLM Title Abbreviation: J Int AIDS Soc
Journal ID: J Int AIDS Soc
NLM Journal ID: 101478566
Journal ID: JIAS

Journal of the International AIDS Society

EISSN: 1758-2652
Publisher: Wiley

ARTICLE INFORMATION
==============================
Article ID: 18442-051
Subjects: D24 - Those growing up with HIV

MOAD0103: The challenges of care and support for a generation of nosocomially infected young adults from Romania living with HIV

Lazar F. 1 §
Buzducea D. 1
1 University of Bucharest, Social Work, Bucharest, Romania
§ Presenting author email: florin.lazar@sas.unibuc.ro
Electronic publication date: 2012 Oct 22
Publication date: 2012
Volume: 15
Issue: Suppl 3
Electronic Location ID: 18442-051
Copyright: © 2012 F. Lazar et al.
Copyright year: 2012
License: This is an open-access article distributed under the terms of the Creative Commons Attribution License, which permits unrestricted use, distribution, and reproduction in any medium, provided the original work is properly cited.
License URL: http://creativecommons.org/licenses/by/2.0/

JOURNAL INFORMATION
==============================
NLM Title Abbreviation: J Int AIDS Soc
Journal ID: J Int AIDS Soc
NLM Journal ID: 101478566
Journal ID: JIAS

Journal of the International AIDS Society

EISSN: 1758-2652
Publisher: Wiley

ARTICLE INFORMATION
==============================
Article ID: 18442-052
Subjects: D26 - Caregiving

MOAD0301: Mental health and substance use problems among a sample of African American and Latina caregivers of children infected, affected and unaffected by HIV

Santamaria E.K. 1 §
Elkington K.S. 1
Alicea S. 2
Dolezal C. 1
Leu C.-S. 1
Mellins C.A. 1
1 HIV Center for Clinical & Behavioral Studies, New York State Psychiatric Institute and Columbia University, New York, United States
2 New York University Steinhardt School of Education, Applied Psychology, New York, United States
§ Presenting author email: santamar@nyspi.columbia.edu
Electronic publication date: 2012 Oct 22
Publication date: 2012
Volume: 15
Issue: Suppl 3
Electronic Location ID: 18442-052
Copyright: © 2012 E.K. Santamaria et al.
Copyright year: 2012
License: This is an open-access article distributed under the terms of the Creative Commons Attribution License, which permits unrestricted use, distribution, and reproduction in any medium, provided the original work is properly cited.
License URL: http://creativecommons.org/licenses/by/2.0/

JOURNAL INFORMATION
==============================
NLM Title Abbreviation: J Int AIDS Soc
Journal ID: J Int AIDS Soc
NLM Journal ID: 101478566
Journal ID: JIAS

Journal of the International AIDS Society

EISSN: 1758-2652
Publisher: Wiley

ARTICLE INFORMATION
==============================
Article ID: 18442-053
Subjects: D26 - Caregiving

MOAD0303: Gendered violence, gender inequalities and home-based care: the forgotten relationship of power

Gibbs A. 1 §
Washington L. 2
Mbatha N. 2
1 University of KwaZulu-Natal, Health Economics and HIV/AIDS Research Division (HEARD), Durban, South Africa
2 Project Empower, Durban, South Africa
§ Presenting author email: gibbs@ukzn.ac.za
Electronic publication date: 2012 Oct 22
Publication date: 2012
Volume: 15
Issue: Suppl 3
Electronic Location ID: 18442-053
Copyright: © 2012 A. Gibbs et al.
Copyright year: 2012
License: This is an open-access article distributed under the terms of the Creative Commons Attribution License, which permits unrestricted use, distribution, and reproduction in any medium, provided the original work is properly cited.
License URL: http://creativecommons.org/licenses/by/2.0/

JOURNAL INFORMATION
==============================
NLM Title Abbreviation: J Int AIDS Soc
Journal ID: J Int AIDS Soc
NLM Journal ID: 101478566
Journal ID: JIAS

Journal of the International AIDS Society

EISSN: 1758-2652
Publisher: Wiley

ARTICLE INFORMATION
==============================
Article ID: 18442-054
Subjects: D27 - Disability

FRLBD03: The impact of depression on retention in care and viral suppression in a large cohort of insured HIV-infected patients

Hechter R.C. 1 §
Wang J.Q. 1
Sidell M.A. 1
Towner W.J. 2
1 Kaiser Permanente Southern California, Research and Evaluation, Pasadena, United States
2 Kaiser Permanente Southern California, Infectious Disease, Los Angeles, United States
§ Presenting author email: rulin.c.hechter@kp.org
Electronic publication date: 2012 Oct 22
Publication date: 2012
Volume: 15
Issue: Suppl 3
Electronic Location ID: 18442-054
Copyright: © 2012 R.C. Hechter et al.
Copyright year: 2012
License: This is an open-access article distributed under the terms of the Creative Commons Attribution License, which permits unrestricted use, distribution, and reproduction in any medium, provided the original work is properly cited.
License URL: http://creativecommons.org/licenses/by/2.0/

JOURNAL INFORMATION
==============================
NLM Title Abbreviation: J Int AIDS Soc
Journal ID: J Int AIDS Soc
NLM Journal ID: 101478566
Journal ID: JIAS

Journal of the International AIDS Society

EISSN: 1758-2652
Publisher: Wiley

ARTICLE INFORMATION
==============================
Article ID: 18442-055
Subjects: D29 - Sexualities: meanings, identities, norms and communities

WEAD0101: A longitudinal in-depth study of gender-specific experiences in antiretroviral treatment patients in South Africa

de Wet K. 1 §
Wouters E. 2
1 Univiversity of the Free State, Sociology, Bloemfontein, South Africa
2 University of Antwerp, Antwerp, Belgium
§ Presenting author email: dewetk@ufs.ac.za
Electronic publication date: 2012 Oct 22
Publication date: 2012
Volume: 15
Issue: Suppl 3
Electronic Location ID: 18442-055
Copyright: © 2012 K. de Wet et al.
Copyright year: 2012
License: This is an open-access article distributed under the terms of the Creative Commons Attribution License, which permits unrestricted use, distribution, and reproduction in any medium, provided the original work is properly cited.
License URL: http://creativecommons.org/licenses/by/2.0/

JOURNAL INFORMATION
==============================
NLM Title Abbreviation: J Int AIDS Soc
Journal ID: J Int AIDS Soc
NLM Journal ID: 101478566
Journal ID: JIAS

Journal of the International AIDS Society

EISSN: 1758-2652
Publisher: Wiley

ARTICLE INFORMATION
==============================
Article ID: 18442-056
Subjects: D29 - Sexualities: meanings, identities, norms and communities

FRLBD06: ‘Privileging the personal': mobilising marginalised identities and voices in the context of HIV health promotion with ”hard to reach'' migrant and second generation Asian gay men in Metropolitan Sydney, Australia; a resource development and community building initiative

Teh M.F. 1 §
1 ACON Health, Community Development, HIV Education (Asian Gay Men's), Sydney, Australia
§ Presenting author email: mteh@acon.org.au
Electronic publication date: 2012 Oct 22
Publication date: 2012
Volume: 15
Issue: Suppl 3
Electronic Location ID: 18442-056
Copyright: © 2012 M.F. Teh et al.
Copyright year: 2012
License: This is an open-access article distributed under the terms of the Creative Commons Attribution License, which permits unrestricted use, distribution, and reproduction in any medium, provided the original work is properly cited.
License URL: http://creativecommons.org/licenses/by/2.0/

JOURNAL INFORMATION
==============================
NLM Title Abbreviation: J Int AIDS Soc
Journal ID: J Int AIDS Soc
NLM Journal ID: 101478566
Journal ID: JIAS

Journal of the International AIDS Society

EISSN: 1758-2652
Publisher: Wiley

ARTICLE INFORMATION
==============================
Article ID: 18442-057
Subjects: D30 - Sexual minorities and HIV vulnerability and/or resilience (e.g., gay and other men who have sex with men (MSM), lesbians and other women who have sex with women (WSW), bisexuals, transgenders, transsexuals

THAD0504: Relationship between comfort with and disclosure about sexual orientation on HIV-related risk behaviours and HIV testing among men who have sex with men in Beirut, Lebanon

Wagner G.J. 1
Aunon F.M. 1 §
Rana Y. 1
Khouri D. 2
Mokhbat J. 2
1 RAND Corporation, Health, Santa Monica, United States
2 Lebanese AIDS Society, Beirut, Lebanon
§ Presenting author email: faunon@rand.org
Electronic publication date: 2012 Oct 22
Publication date: 2012
Volume: 15
Issue: Suppl 3
Electronic Location ID: 18442-057
Copyright: © 2012 F.M. Aunon et al.
Copyright year: 2012
License: This is an open-access article distributed under the terms of the Creative Commons Attribution License, which permits unrestricted use, distribution, and reproduction in any medium, provided the original work is properly cited.
License URL: http://creativecommons.org/licenses/by/2.0/

JOURNAL INFORMATION
==============================
NLM Title Abbreviation: J Int AIDS Soc
Journal ID: J Int AIDS Soc
NLM Journal ID: 101478566
Journal ID: JIAS

Journal of the International AIDS Society

EISSN: 1758-2652
Publisher: Wiley

ARTICLE INFORMATION
==============================
Article ID: 18442-058
Subjects: D30 - Sexual minorities and HIV vulnerability and/or resilience (e.g., gay and other men who have sex with men (MSM), lesbians and other women who have sex with women (WSW), bisexuals, transgenders, transsexuals

FRLBD05: An MSM-specific definition of intimate partner violence includes HIV-related violence and is associated with sexual risk-taking among MSM

Stephenson R. 1
Finneran C. 1 §
1 Emory University Rollins School of Public Health, Hubert Department of Global Health, Atlanta, United States
§ Presenting author email: cafinne@emory.edu
Electronic publication date: 2012 Oct 22
Publication date: 2012
Volume: 15
Issue: Suppl 3
Electronic Location ID: 18442-058
Copyright: © 2012 C. Finneran et al.
Copyright year: 2012
License: This is an open-access article distributed under the terms of the Creative Commons Attribution License, which permits unrestricted use, distribution, and reproduction in any medium, provided the original work is properly cited.
License URL: http://creativecommons.org/licenses/by/2.0/

JOURNAL INFORMATION
==============================
NLM Title Abbreviation: J Int AIDS Soc
Journal ID: J Int AIDS Soc
NLM Journal ID: 101478566
Journal ID: JIAS

Journal of the International AIDS Society

EISSN: 1758-2652
Publisher: Wiley

ARTICLE INFORMATION
==============================
Article ID: 18442-059
Subjects: D31 - Effects of homophobia and transphobia

THAD0505: Internalized homophobia and transphobia, low self-esteem and non-disclosure of sexual identity as factors contributing to HIV vulnerability of men who have sex with men (MSM), transgenders and hijras: experience from the Global Fund supported Pehchān program in India

Shaikh S. 1 §
Lonappan S. 2
Kumarikunta G. 3
Biswas K. 3
Rakesh S. 4
Aher A. 1
Mehta S.M. 5
Robertson J. 6
1 India HIV/AIDS Alliance, Pehchan, New Delhi, India
2 India HIV/AIDS Alliance, Communications, New Delhi, India
3 India HIV/AIDS Alliance, Monitoring & Evaluation, New Delhi, India
4 India HIV/AIDS Alliance, Technical Support, New Delhi, India
5 India HIV/AIDS Alliance, Policy & Programmes, New Delhi, India
6 India HIV/AIDS Alliance, New Delhi, India
§ Presenting author email: ssimran1888@gmail.com
Electronic publication date: 2012 Oct 22
Publication date: 2012
Volume: 15
Issue: Suppl 3
Electronic Location ID: 18442-059
Copyright: © 2012 S. Shaikh et al.
Copyright year: 2012
License: This is an open-access article distributed under the terms of the Creative Commons Attribution License, which permits unrestricted use, distribution, and reproduction in any medium, provided the original work is properly cited.
License URL: http://creativecommons.org/licenses/by/2.0/

JOURNAL INFORMATION
==============================
NLM Title Abbreviation: J Int AIDS Soc
Journal ID: J Int AIDS Soc
NLM Journal ID: 101478566
Journal ID: JIAS

Journal of the International AIDS Society

EISSN: 1758-2652
Publisher: Wiley

ARTICLE INFORMATION
==============================
Article ID: 18442-060
Subjects: D32 - Young people and sexuality, intergenerational sex

THAD0502: Homophobia and access to HIV services among young men who have sex with men (YMSM)

Santos G.-M. 1 2
Beck J. 3
Wilson P. 4
Hebert P. 3
Ayala G. 3
1 University of California, San Francisco, School of Medicine, Department of Epidemiology and Biostatistics, San Francisco, United States
2 San Francisco Department of Public Health, HIV Prevention and Research Section, San Francisco, United States
3 Global Forum on MSM & HIV (MSMGF), Oakland, United States
4 Columbia University, Mailman School of Public Health, Department of Sociomedical Sciences, New York, United States
G.-M. Santos,
Electronic publication date: 2012 Oct 22
Publication date: 2012
Volume: 15
Issue: Suppl 3
Electronic Location ID: 18442-060
Copyright: © 2012 G.-M. Santos et al.
Copyright year: 2012
License: This is an open-access article distributed under the terms of the Creative Commons Attribution License, which permits unrestricted use, distribution, and reproduction in any medium, provided the original work is properly cited.
License URL: http://creativecommons.org/licenses/by/2.0/

JOURNAL INFORMATION
==============================
NLM Title Abbreviation: J Int AIDS Soc
Journal ID: J Int AIDS Soc
NLM Journal ID: 101478566
Journal ID: JIAS

Journal of the International AIDS Society

EISSN: 1758-2652
Publisher: Wiley

ARTICLE INFORMATION
==============================
Article ID: 18442-061
Subjects: D33 - Relationships, concurrency, sexual networks and partnerships

THAD0503: Women partners of men who have sex with men (MSM) in India: preventing HIV transmission and promoting early HIV diagnosis and treatment

Chakrapani V. 1 §
Boyce P. 2
Dhanikachalam D. 3
Manilal N.R. 4
1 Centre for Sexuality and Health Research and Policy (C-SHaRP), Chennai, India
2 University of Sussex, Brighton, United Kingdom
3 Futures Group International India Pvt. Ltd., New Delhi, India
4 National AIDS Control Organisation, New Delhi, India
§ Presenting author email: cvenkatesan@hotmail.com
Electronic publication date: 2012 Oct 22
Publication date: 2012
Volume: 15
Issue: Suppl 3
Electronic Location ID: 18442-061
Copyright: © 2012 V. Chakrapani et al.
Copyright year: 2012
License: This is an open-access article distributed under the terms of the Creative Commons Attribution License, which permits unrestricted use, distribution, and reproduction in any medium, provided the original work is properly cited.
License URL: http://creativecommons.org/licenses/by/2.0/

JOURNAL INFORMATION
==============================
NLM Title Abbreviation: J Int AIDS Soc
Journal ID: J Int AIDS Soc
NLM Journal ID: 101478566
Journal ID: JIAS

Journal of the International AIDS Society

EISSN: 1758-2652
Publisher: Wiley

ARTICLE INFORMATION
==============================
Article ID: 18442-062
Subjects: D33 - Relationships, concurrency, sexual networks and partnerships

WEPDE0204: Social network of male sex workers in Houston, Texas

Ha D. 1 §
Fujimoto K. 1
Ross M. 1
1 The University of Texas, School of Public Health at Houston, Health Promotion and Behavioral Sciences, Houston, United States
§ Presenting author email: doan.t.ha@uth.tmc.edu
Electronic publication date: 2012 Oct 22
Publication date: 2012
Volume: 15
Issue: Suppl 3
Electronic Location ID: 18442-062
Copyright: © 2012 D. Ha et al.
Copyright year: 2012
License: This is an open-access article distributed under the terms of the Creative Commons Attribution License, which permits unrestricted use, distribution, and reproduction in any medium, provided the original work is properly cited.
License URL: http://creativecommons.org/licenses/by/2.0/

JOURNAL INFORMATION
==============================
NLM Title Abbreviation: J Int AIDS Soc
Journal ID: J Int AIDS Soc
NLM Journal ID: 101478566
Journal ID: JIAS

Journal of the International AIDS Society

EISSN: 1758-2652
Publisher: Wiley

ARTICLE INFORMATION
==============================
Article ID: 18442-063
Subjects: D35 - Reproductive health, fertility, family planning

WEAE0406: ‘Condoms are not a family planning method’: how efforts to prevent HIV have failed to comprehensively address adolescent sexual and reproductive health

Adams M. 1 §
Johnson H. 2
Lundgren R. 1
1 Georgetown University, Institute for Reproductive Health, Washington, United States
2 Georgeown Universtiy, Government, Conflict Resolution Program, Washington, United States
§ Presenting author email: mka46@georgetown.edu
Electronic publication date: 2012 Oct 22
Publication date: 2012
Volume: 15
Issue: Suppl 3
Electronic Location ID: 18442-063
Copyright: © 2012 M. Adams et al.
Copyright year: 2012
License: This is an open-access article distributed under the terms of the Creative Commons Attribution License, which permits unrestricted use, distribution, and reproduction in any medium, provided the original work is properly cited.
License URL: http://creativecommons.org/licenses/by/2.0/

JOURNAL INFORMATION
==============================
NLM Title Abbreviation: J Int AIDS Soc
Journal ID: J Int AIDS Soc
NLM Journal ID: 101478566
Journal ID: JIAS

Journal of the International AIDS Society

EISSN: 1758-2652
Publisher: Wiley

ARTICLE INFORMATION
==============================
Article ID: 18442-064
Subjects: D37 - Sex work and sex cultures

TUAD0303: The complexity of negotiating HIV risk with clients and in private relationships: experiences of female sex workers in India

Panchanadeswaran S. 1 2 §
Hwahng S. 3
Chacko S. 4
1 Adelphi University, School of Social Work, New York, United States
2 Johns Hopkins Bloomberg School of Public Health, Baltimore, United States
3 Columbia University, Center for the Study of Ethnicity and Race, New York, United States
4 Aneka, Bangalore, India
§ Presenting author email: panchanadeswaran@adelphi.edu
Electronic publication date: 2012 Oct 22
Publication date: 2012
Volume: 15
Issue: Suppl 3
Electronic Location ID: 18442-064
Copyright: © 2012 S. Panchanadeswaran et al.
Copyright year: 2012
License: This is an open-access article distributed under the terms of the Creative Commons Attribution License, which permits unrestricted use, distribution, and reproduction in any medium, provided the original work is properly cited.
License URL: http://creativecommons.org/licenses/by/2.0/

JOURNAL INFORMATION
==============================
NLM Title Abbreviation: J Int AIDS Soc
Journal ID: J Int AIDS Soc
NLM Journal ID: 101478566
Journal ID: JIAS

Journal of the International AIDS Society

EISSN: 1758-2652
Publisher: Wiley

ARTICLE INFORMATION
==============================
Article ID: 18442-065
Subjects: D37 - Sex work and sex cultures

TUAD0304: Emotional men and pragmatic women: relationship and gender dynamics between female sex workers and their regular partners in the Dominican Republic

Barrington C. 1
Fleming P. 1
Moya M. 2
Rosario S. 2
Perez M. 3
Donastorg Y. 3
Broxton C. 4
Kerrigan D. 5 §
1 University of North Carolina at Chapel Hill, Gillings School of Global Public Health, Department of Health Behavior and Health Education, Chapel Hill, United States
2 Centro de Orientacion e Investigación Integral (COIN), Santo Domingo, Dominican Republic
3 Unidad de Vacunas e Investigacion (IDCP-COIN-DIGECITSS), Santo Domingo, Dominican Republic
4 USAID, Washington, United States
5 Johns Hopkins Bloomberg School of Public Health, Baltimore, United States
§ Presenting author email: dkerriga@jhsph.edu
Electronic publication date: 2012 Oct 22
Publication date: 2012
Volume: 15
Issue: Suppl 3
Electronic Location ID: 18442-065
Copyright: © 2012 D. Kerrigan et al.
Copyright year: 2012
License: This is an open-access article distributed under the terms of the Creative Commons Attribution License, which permits unrestricted use, distribution, and reproduction in any medium, provided the original work is properly cited.
License URL: http://creativecommons.org/licenses/by/2.0/

JOURNAL INFORMATION
==============================
NLM Title Abbreviation: J Int AIDS Soc
Journal ID: J Int AIDS Soc
NLM Journal ID: 101478566
Journal ID: JIAS

Journal of the International AIDS Society

EISSN: 1758-2652
Publisher: Wiley

ARTICLE INFORMATION
==============================
Article ID: 18442-066
Subjects: D37 - Sex work and sex cultures

TUAD0305: Older women and sex work: breaking the silence on the gendered structural drivers of HIV in Uganda

Zalwango Kabuye F. 1 §
Nakamanya S. 1
Nalugya R. 1
Nnalusiba B. 1
Seeley J. 1 2
1 Medical Research Council/Uganda Virus Research Institute, Social Science, Entebbe, Uganda
2 University of East Anglia, School of International Development, Norwich, United Kingdom
§ Presenting author email: flavia.zalwango@mrcuganda.org
Electronic publication date: 2012 Oct 22
Publication date: 2012
Volume: 15
Issue: Suppl 3
Electronic Location ID: 18442-066
Copyright: © 2012 F. Zalwango Kabuye et al.
Copyright year: 2012
License: This is an open-access article distributed under the terms of the Creative Commons Attribution License, which permits unrestricted use, distribution, and reproduction in any medium, provided the original work is properly cited.
License URL: http://creativecommons.org/licenses/by/2.0/

JOURNAL INFORMATION
==============================
NLM Title Abbreviation: J Int AIDS Soc
Journal ID: J Int AIDS Soc
NLM Journal ID: 101478566
Journal ID: JIAS

Journal of the International AIDS Society

EISSN: 1758-2652
Publisher: Wiley

ARTICLE INFORMATION
==============================
Article ID: 18442-067
Subjects: D38 - Sex, gender and new prevention technologies

FRLBD04: Will and should women in the U.S. use PrEP? Findings from a focus group study of at-risk, HIV-negative women in Oakland, Memphis, San Diego and Washington, D.C.

Auerbach J. 1 §
Banyan A. 2
Riordan M. 3
1 Independent Consultant, San Francisco, United States
2 AIDS United, Consultant, Oakland, United States
3 AIDS United, Washington, United States
§ Presenting author . Email: judithd.auerbach@gmail.com
Electronic publication date: 2012 Oct 22
Publication date: 2012
Volume: 15
Issue: Suppl 3
Electronic Location ID: 18442-067
Copyright: © 2012 J. Auerbach et al.
Copyright year: 2012
License: This is an open-access article distributed under the terms of the Creative Commons Attribution License, which permits unrestricted use, distribution, and reproduction in any medium, provided the original work is properly cited.
License URL: http://creativecommons.org/licenses/by/2.0/

JOURNAL INFORMATION
==============================
NLM Title Abbreviation: J Int AIDS Soc
Journal ID: J Int AIDS Soc
NLM Journal ID: 101478566
Journal ID: JIAS

Journal of the International AIDS Society

EISSN: 1758-2652
Publisher: Wiley

ARTICLE INFORMATION
==============================
Article ID: 18442-068
Subjects: D41 - Social networks of drug users

MOAD0401: The real risks of fishing: social networks and HIV among drug-using fishermen in Malaysia

West B.S. 1 2
Choo M. 3
El-Bassel N. 4
Kamarulzaman A. 3
Wu E. 4
Gilbert L. 4
1 Columbia University, Sociomedical Sciences, New York, United States
2 National Development and Research Institutes, Inc., Institute of AIDS Research, New York, United States
3 Center of Excellence for Research in AIDS (CERiA), Department of Medicine, University of Malaya, Kuala Lumpur, Malaysia
4 Columbia University School of Social Work, Social Intervention Group, New York, United States
B.S. West,
Electronic publication date: 2012 Oct 22
Publication date: 2012
Volume: 15
Issue: Suppl 3
Electronic Location ID: 18442-068
Copyright: © 2012 B.S. West et al.
Copyright year: 2012
License: This is an open-access article distributed under the terms of the Creative Commons Attribution License, which permits unrestricted use, distribution, and reproduction in any medium, provided the original work is properly cited.
License URL: http://creativecommons.org/licenses/by/2.0/

JOURNAL INFORMATION
==============================
NLM Title Abbreviation: J Int AIDS Soc
Journal ID: J Int AIDS Soc
NLM Journal ID: 101478566
Journal ID: JIAS

Journal of the International AIDS Society

EISSN: 1758-2652
Publisher: Wiley

ARTICLE INFORMATION
==============================
Article ID: 18442-069
Subjects: D42 - Sexual transmission and drug use

TUPDD0201: Typology of polydrug use and unsafe sex practices among rural stimulant users

Wang J. 1
Kelly B. 2 §
1 Childrens National Medical Center, Center for Clinical and Community Research, Washington, United States
2 Purdue University, Sociology, West Lafayette, United States
§ Presenting author email: bckelly@purdue.edu
Electronic publication date: 2012 Oct 22
Publication date: 2012
Volume: 15
Issue: Suppl 3
Electronic Location ID: 18442-069
Copyright: © 2012 B. Kelly et al.
Copyright year: 2012
License: This is an open-access article distributed under the terms of the Creative Commons Attribution License, which permits unrestricted use, distribution, and reproduction in any medium, provided the original work is properly cited.
License URL: http://creativecommons.org/licenses/by/2.0/

JOURNAL INFORMATION
==============================
NLM Title Abbreviation: J Int AIDS Soc
Journal ID: J Int AIDS Soc
NLM Journal ID: 101478566
Journal ID: JIAS

Journal of the International AIDS Society

EISSN: 1758-2652
Publisher: Wiley

ARTICLE INFORMATION
==============================
Article ID: 18442-070
Subjects: D42 - Sexual transmission and drug use

TUPDD0202: Predictors of long-term trajectories (2003–2010) of sex-drug and heavy alcohol (SDA) use among MSM

Ostrow D.G. 1 2 §
Stall R.C. 3
Jantz I. 4
Berona J. 5
Herrick A. 3
Carrico A. 6
Swartz J. 4
Long Term Effects of Meth Use Study Group
1 David Ostrow & Associates, LLC, Chicago, United States
2 National Opinion Research Center (NORC) at the University of Chicago, Ogburn-Stouffer Center for Social Organizational Research, Chicago, United States
3 University of Pittsburgh, Graduate School of Public Health, Dept of Behavioral and Community Health Studies, Pittsburgh, United States
4 University of Illinois at Chicago, Jane Adams College of Social Work, Chicago, United States
5 University of Michigan, Clinical Psychology, Ann Arbor, United States
6 University of California at San Francisco, Center for AIDS Prevention Research, San Francisco, United States
§ Presenting author email: bigdavido@aol.com
Electronic publication date: 2012 Oct 22
Publication date: 2012
Volume: 15
Issue: Suppl 3
Electronic Location ID: 18442-070
Copyright: © 2012 D.G. Ostrow et al.
Copyright year: 2012
License: This is an open-access article distributed under the terms of the Creative Commons Attribution License, which permits unrestricted use, distribution, and reproduction in any medium, provided the original work is properly cited.
License URL: http://creativecommons.org/licenses/by/2.0/

JOURNAL INFORMATION
==============================
NLM Title Abbreviation: J Int AIDS Soc
Journal ID: J Int AIDS Soc
NLM Journal ID: 101478566
Journal ID: JIAS

Journal of the International AIDS Society

EISSN: 1758-2652
Publisher: Wiley

ARTICLE INFORMATION
==============================
Article ID: 18442-071
Subjects: D42 - Sexual transmission and drug use

TUPDD0203: Correlation between illicit substances, unsafe sexual behaviour and symptoms suggesting sexually transmitted infections among male clients of female sex workers in Bangladesh

Ahmed A. 1 §
Reichenbach L. 1
Alam N. 1
1 International Centre for Diarrhoeal Disease Research (ICDDR), Centre for Reproductive Health, Dhaka, Bangladesh
§ Presenting author email: anisuddin@icddrb.org
Electronic publication date: 2012 Oct 22
Publication date: 2012
Volume: 15
Issue: Suppl 3
Electronic Location ID: 18442-071
Copyright: © 2012 A. Ahmed et al.
Copyright year: 2012
License: This is an open-access article distributed under the terms of the Creative Commons Attribution License, which permits unrestricted use, distribution, and reproduction in any medium, provided the original work is properly cited.
License URL: http://creativecommons.org/licenses/by/2.0/

JOURNAL INFORMATION
==============================
NLM Title Abbreviation: J Int AIDS Soc
Journal ID: J Int AIDS Soc
NLM Journal ID: 101478566
Journal ID: JIAS

Journal of the International AIDS Society

EISSN: 1758-2652
Publisher: Wiley

ARTICLE INFORMATION
==============================
Article ID: 18442-072
Subjects: D42 - Sexual transmission and drug use

TUPDD0204: Getting high, getting laid: injecting practices and sexual behaviour of people who inject drugs (PWID) in three Indian states (findings from the Hridaya baseline study)

Biswas K. 1 §
Arumugam V. 1
Sharma C. 2
Rakesh S. 3
Robertson J. 4
1 India HIV/AIDS Alliance, Monitoring & Evaluation, New Delhi, India
2 India HIV/AIDS Alliance, Programmes & Policy, New Delhi, India
3 India HIV/AIDS Alliance, Technical Support, New Delhi, India
4 India HIV/AIDS Alliance, Country Directorate, New Delhi, India
§ Presenting author email: kbiswas@allianceindia.org
Electronic publication date: 2012 Oct 22
Publication date: 2012
Volume: 15
Issue: Suppl 3
Electronic Location ID: 18442-072
Copyright: © 2012 K. Biswas et al.
Copyright year: 2012
License: This is an open-access article distributed under the terms of the Creative Commons Attribution License, which permits unrestricted use, distribution, and reproduction in any medium, provided the original work is properly cited.
License URL: http://creativecommons.org/licenses/by/2.0/

JOURNAL INFORMATION
==============================
NLM Title Abbreviation: J Int AIDS Soc
Journal ID: J Int AIDS Soc
NLM Journal ID: 101478566
Journal ID: JIAS

Journal of the International AIDS Society

EISSN: 1758-2652
Publisher: Wiley

ARTICLE INFORMATION
==============================
Article ID: 18442-073
Subjects: D42 - Sexual transmission and drug use

TUPDD0205: Substance use and high-risk sexual behavior: dose-response associations in episodic and high-frequency substance-using men who have sex with men (SUMSM)

Santos G.-M. 1 2 §
Das M. 1 3
Matheson T. 1
Demicco E. 1
Vittinghoff E. 2
Dilley J. 4
Colfax G. 1 3
1 San Francisco Department of Public Health, HIV Prevention Section, San Francisco, United States
2 University of California San Francisco, School of Medicine, Epidemiology and Biostatistics, San Francisco, United States
3 University of California San Francisco, Divisions of HIV/AIDS and Infectious Diseases, SFGH, San Francisco, United States
4 University of California San Francisco, Psychiatry, San Francisco, United States
§ Presenting author email: glenn-milo.santos@sfdph.org
Electronic publication date: 2012 Oct 22
Publication date: 2012
Volume: 15
Issue: Suppl 3
Electronic Location ID: 18442-073
Copyright: © 2012 G.-M. Santos et al.
Copyright year: 2012
License: This is an open-access article distributed under the terms of the Creative Commons Attribution License, which permits unrestricted use, distribution, and reproduction in any medium, provided the original work is properly cited.
License URL: http://creativecommons.org/licenses/by/2.0/

JOURNAL INFORMATION
==============================
NLM Title Abbreviation: J Int AIDS Soc
Journal ID: J Int AIDS Soc
NLM Journal ID: 101478566
Journal ID: JIAS

Journal of the International AIDS Society

EISSN: 1758-2652
Publisher: Wiley

ARTICLE INFORMATION
==============================
Article ID: 18442-074
Subjects: D43 - Criminalization of drug use and people who use drugs and its impact on HIV

MOAD0204: The impact of police violence on HIV risks among people who inject drugs in Thailand

Hayashi K. 1 §
Ti L. 1
Kaplan K. 2
Suwannawong P. 2
Shannon K. 1
Wood E. 1
Kerr T. 1
1 British Columbia Centre for Excellence in HIV/AIDS, Vancouver, Canada
2 Thai AIDS Treatment Action Group, Bangkok, Thailand
§ Presenting author email: khayashi@cfenet.ubc.ca
Electronic publication date: 2012 Oct 22
Publication date: 2012
Volume: 15
Issue: Suppl 3
Electronic Location ID: 18442-074
Copyright: © 2012 K. Hayashi et al.
Copyright year: 2012
License: This is an open-access article distributed under the terms of the Creative Commons Attribution License, which permits unrestricted use, distribution, and reproduction in any medium, provided the original work is properly cited.
License URL: http://creativecommons.org/licenses/by/2.0/

JOURNAL INFORMATION
==============================
NLM Title Abbreviation: J Int AIDS Soc
Journal ID: J Int AIDS Soc
NLM Journal ID: 101478566
Journal ID: JIAS

Journal of the International AIDS Society

EISSN: 1758-2652
Publisher: Wiley

ARTICLE INFORMATION
==============================
Article ID: 18442-075
Subjects: D44 - Harm reduction programmes, including syringe exchange, substitution therapy and supervised injection facilities

MOAD0402: Global Fund investments in harm reduction from 2002 to 2009

Bridge J. 1
Hunter B.M. 1
Atun R. 1 2
Lazarus J.V. 1 3 §
1 Global Fund to Fight AIDS, Tuberculosis and Malaria, Strategy, Performance and Evaluation Cluster, Geneva, Switzerland
2 Imperial College London, London, United Kingdom
3 Copenhagen HIV Programme, University of Copenhagen, Copenhagen, Denmark
§ Presenting author email: jla@cphiv.dk
Electronic publication date: 2012 Oct 22
Publication date: 2012
Volume: 15
Issue: Suppl 3
Electronic Location ID: 18442-075
Copyright: © 2012 J.V. Lazarus et al.
Copyright year: 2012
License: This is an open-access article distributed under the terms of the Creative Commons Attribution License, which permits unrestricted use, distribution, and reproduction in any medium, provided the original work is properly cited.
License URL: http://creativecommons.org/licenses/by/2.0/

JOURNAL INFORMATION
==============================
NLM Title Abbreviation: J Int AIDS Soc
Journal ID: J Int AIDS Soc
NLM Journal ID: 101478566
Journal ID: JIAS

Journal of the International AIDS Society

EISSN: 1758-2652
Publisher: Wiley

ARTICLE INFORMATION
==============================
Article ID: 18442-076
Subjects: D44 - Harm reduction programmes, including syringe exchange, substitution therapy and supervised injection facilities

MOAD0403: Opioid substitution therapy in Eurasia: how to increase the access and improve the quality

Latypov A. 1 §
Bidordinova A. 2
Khachatrian A. 2
1 Eurasian Harm Reduction Network, Research and Information Program, Vilnius, Lithuania
2 Independent Consultant, Toronto, Canada
§ Presenting author email: alisher_latypov@fulbrightmail.org
Electronic publication date: 2012 Oct 22
Publication date: 2012
Volume: 15
Issue: Suppl 3
Electronic Location ID: 18442-076
Copyright: © 2012 A. Latypov et al.
Copyright year: 2012
License: This is an open-access article distributed under the terms of the Creative Commons Attribution License, which permits unrestricted use, distribution, and reproduction in any medium, provided the original work is properly cited.
License URL: http://creativecommons.org/licenses/by/2.0/

JOURNAL INFORMATION
==============================
NLM Title Abbreviation: J Int AIDS Soc
Journal ID: J Int AIDS Soc
NLM Journal ID: 101478566
Journal ID: JIAS

Journal of the International AIDS Society

EISSN: 1758-2652
Publisher: Wiley

ARTICLE INFORMATION
==============================
Article ID: 18442-077
Subjects: D44 - Harm reduction programmes, including syringe exchange, substitution therapy and supervised injection facilities

MOAD0404: A qualitative study on reasons for relatively low methadone dosing among persons who inject drugs in three provinces in China

Han L. 1
Li Z. 1
Luo W. 2
Bulterys M. 1
Yang F. 3
Li R. 4
Shen L. 5
Fuller S. 6
Wu Z. 2
1 U.S. CDC Division of Global HIV/AIDS, Beijing, China
2 National Center for AIDS/STD Control and Prevention, Chinese Center for Disease Control and Prevention, Beijing, China
3 Guangdong Provincial CDC, Division of AIDS/STD Control and Prevention, Guangzhou, China
4 Guangxi Zhuang Autonomous Region CDC, Division for HIV/AIDS Control and Prevention, Nanning, China
5 Guizhou Provinial CDC, Division of AIDS, Dermatosis and Leprosy Control and Prevention, Guiyang, China
6 Association of Schools of Public Health, Washington, United States
L. Han,
Electronic publication date: 2012 Oct 22
Publication date: 2012
Volume: 15
Issue: Suppl 3
Electronic Location ID: 18442-077
Copyright: © 2012 L. Han et al.
Copyright year: 2012
License: This is an open-access article distributed under the terms of the Creative Commons Attribution License, which permits unrestricted use, distribution, and reproduction in any medium, provided the original work is properly cited.
License URL: http://creativecommons.org/licenses/by/2.0/

JOURNAL INFORMATION
==============================
NLM Title Abbreviation: J Int AIDS Soc
Journal ID: J Int AIDS Soc
NLM Journal ID: 101478566
Journal ID: JIAS

Journal of the International AIDS Society

EISSN: 1758-2652
Publisher: Wiley

ARTICLE INFORMATION
==============================
Article ID: 18442-078
Subjects: D45 - Positive health, dignity, psychological well being, mental health and prevention

MOAD0105: Resiliency among urban youth newly diagnosed with HIV in Kenya: sources of social support and active coping strategies

Harper G. 1 2 §
Ngugi E. 3
Riplinger A. 1
Gikuni A. 3
Lemos D. 2
Hosek S. 4
Hooks K. 2
1 DePaul University, Master of Public Health (MPH) Program, Chicago, United States
2 DePaul University, Department of Pscyhology, Chicago, United States
3 University of Nairobi, Centre for HIV Prevention and Research, Nairobi, Kenya
4 Stroger Hospital of Cook County, Chicago, United States
§ Presenting author email: gharper@depaul.edu
Electronic publication date: 2012 Oct 22
Publication date: 2012
Volume: 15
Issue: Suppl 3
Electronic Location ID: 18442-078
Copyright: © 2012 G. Harper et al.
Copyright year: 2012
License: This is an open-access article distributed under the terms of the Creative Commons Attribution License, which permits unrestricted use, distribution, and reproduction in any medium, provided the original work is properly cited.
License URL: http://creativecommons.org/licenses/by/2.0/

JOURNAL INFORMATION
==============================
NLM Title Abbreviation: J Int AIDS Soc
Journal ID: J Int AIDS Soc
NLM Journal ID: 101478566
Journal ID: JIAS

Journal of the International AIDS Society

EISSN: 1758-2652
Publisher: Wiley

ARTICLE INFORMATION
==============================
Article ID: 18442-079
Subjects: D45 - Positive health, dignity, psychological well being, mental health and prevention

MOAD0302: Living with HIV increases risk for poor mental health among adults caring for orphaned children in South Africa

Kuo C. 1 2 §
Casale M. 3
Cluver L. 4
Lane T. 4
Sello L. 3
1 Brown University, Psychiatry and Human Behavior, Providence, United States
2 Rhode Island Hospital, Providence, United States
3 University of KwaZulu Natal, Health Economics and HIV/AIDS Research Division, Durban, South Africa
4 Oxford University, Department of Social Policy and Intervention, Oxford, United Kingdom
§ Presenting author email: carolineckuo@yahoo.com
Electronic publication date: 2012 Oct 22
Publication date: 2012
Volume: 15
Issue: Suppl 3
Electronic Location ID: 18442-079
Copyright: © 2012 C. Kuo et al.
Copyright year: 2012
License: This is an open-access article distributed under the terms of the Creative Commons Attribution License, which permits unrestricted use, distribution, and reproduction in any medium, provided the original work is properly cited.
License URL: http://creativecommons.org/licenses/by/2.0/

JOURNAL INFORMATION
==============================
NLM Title Abbreviation: J Int AIDS Soc
Journal ID: J Int AIDS Soc
NLM Journal ID: 101478566
Journal ID: JIAS

Journal of the International AIDS Society

EISSN: 1758-2652
Publisher: Wiley

ARTICLE INFORMATION
==============================
Article ID: 18442-080
Subjects: D45 - Positive health, dignity, psychological well being, mental health and prevention

WEPDE0103: Addressing the unmet sexual and reproductive health and rights (SRHR) of people living with HIV (PLHIV): the results from a baseline study in four states in India

Biswas K. 1 §
Manikandakumar T. 1
Grote S. 2
Pal K. 2
Mehta S. 2
Robertson J. 3
1 India HIV/AIDS Alliance, Monitoring & Evaluation, New Delhi, India
2 India HIV/AIDS Alliance, Policy & Programmes, New Delhi, India
3 India HIV/AIDS Alliance, Country Directorate, New Delhi, India
§ Presenting author email: kbiswas@allianceindia.org
Electronic publication date: 2012 Oct 22
Publication date: 2012
Volume: 15
Issue: Suppl 3
Electronic Location ID: 18442-080
Copyright: © 2012 K. Biswas et al.
Copyright year: 2012
License: This is an open-access article distributed under the terms of the Creative Commons Attribution License, which permits unrestricted use, distribution, and reproduction in any medium, provided the original work is properly cited.
License URL: http://creativecommons.org/licenses/by/2.0/

JOURNAL INFORMATION
==============================
NLM Title Abbreviation: J Int AIDS Soc
Journal ID: J Int AIDS Soc
NLM Journal ID: 101478566
Journal ID: JIAS

Journal of the International AIDS Society

EISSN: 1758-2652
Publisher: Wiley

ARTICLE INFORMATION
==============================
Article ID: 18442-081
Subjects: D46 - Adaptation to living with HIV for individuals, families and communities

MOAD0102: Challenges and coping mechanisms of HIV-positive school age children in Botswana

Letamo G. 1
Karugaba G. 2 3
Marukutira T. 2 3
Mabikwa V. 2 3
Makhanda J. 2 3
Marape M. 2 3 4
Seleke R. 2 3
Anabwani G.M. 2 3 4 §
1 University of Botswana, Gaborone, Botswana
2 Botswana-Baylor Children's Clinical Center of Excellence, Gaborone, Botswana
3 Baylor International Pediatric AIDS Initiative, Baylor College of Medicine, Pediatric Retrovirology, Houston, United States
4 Texas Children's Hospital, Pediatrics, Houston, United States
§ Presenting author email: ganabwani@baylorbotswana.org.bw
Electronic publication date: 2012 Oct 22
Publication date: 2012
Volume: 15
Issue: Suppl 3
Electronic Location ID: 18442-081
Copyright: © 2012 G.M. Anabwani et al.
Copyright year: 2012
License: This is an open-access article distributed under the terms of the Creative Commons Attribution License, which permits unrestricted use, distribution, and reproduction in any medium, provided the original work is properly cited.
License URL: http://creativecommons.org/licenses/by/2.0/

JOURNAL INFORMATION
==============================
NLM Title Abbreviation: J Int AIDS Soc
Journal ID: J Int AIDS Soc
NLM Journal ID: 101478566
Journal ID: JIAS

Journal of the International AIDS Society

EISSN: 1758-2652
Publisher: Wiley

ARTICLE INFORMATION
==============================
Article ID: 18442-082
Subjects: D47 - Seropositivity: social identity, disclosure and vulnerability

THAD0501: HIV-positive gay and bisexual men in Peru: sexuality, disclosure and the disconnect between sexual health needs and access

Caceres C.F. 1
Salazar X. 1
Ceccarelli M.A. 1 §
Anamaria P. 2
Prada P. 2
Villayzan J. 3
Bracamonte P. 4
1 Cayetano Heredia University School of Public Health, Institute of Health, Sexuality and Human Development, Lima, Peru
2 Peruanos Positivos, Lima, Peru
3 Red Peru Trans, Lima, Peru
4 ONUSIDA, Lima, Peru
§ Presenting author email: carlos.caceres@upch.pe
Electronic publication date: 2012 Oct 22
Publication date: 2012
Volume: 15
Issue: Suppl 3
Electronic Location ID: 18442-082
Copyright: © 2012 M.A. Ceccarelli et al.
Copyright year: 2012
License: This is an open-access article distributed under the terms of the Creative Commons Attribution License, which permits unrestricted use, distribution, and reproduction in any medium, provided the original work is properly cited.
License URL: http://creativecommons.org/licenses/by/2.0/

JOURNAL INFORMATION
==============================
NLM Title Abbreviation: J Int AIDS Soc
Journal ID: J Int AIDS Soc
NLM Journal ID: 101478566
Journal ID: JIAS

Journal of the International AIDS Society

EISSN: 1758-2652
Publisher: Wiley

ARTICLE INFORMATION
==============================
Article ID: 18442-083
Subjects: D48 - Growing up with HIV: early infection, children and youth

MOAD0104: Experiences and challenges in sexual and reproductive health for adolescents living with HIV in Malawi, Mozambique, Zambia and Zimbabwe

Cataldo F. 1
Malunga A. 1
Rusakaniko S. 2
Umar E. 3
Teles N. 4
Musandu H. 5 §
1 Dignitas International Malawi, Research Department, Zomba, Malawi
2 University of Zimbabwe, Department of Community Medicine, College of Health Sciences, Harare, Zimbabwe
3 University of Malawi, College of Medicine, Blantyre, Malawi
4 University Eduardo Mondlane, Sociology Department, Maputo, Mozambique
5 Southern African AIDS Trust, Johannesburg, South Africa
F. Cataldo,
Electronic publication date: 2012 Oct 22
Publication date: 2012
Volume: 15
Issue: Suppl 3
Electronic Location ID: 18442-083
Copyright: © 2012 F. Cataldo et al.
Copyright year: 2012
License: This is an open-access article distributed under the terms of the Creative Commons Attribution License, which permits unrestricted use, distribution, and reproduction in any medium, provided the original work is properly cited.
License URL: http://creativecommons.org/licenses/by/2.0/

JOURNAL INFORMATION
==============================
NLM Title Abbreviation: J Int AIDS Soc
Journal ID: J Int AIDS Soc
NLM Journal ID: 101478566
Journal ID: JIAS

Journal of the International AIDS Society

EISSN: 1758-2652
Publisher: Wiley

ARTICLE INFORMATION
==============================
Article ID: 18442-084
Subjects: D53 - Ageing with HIV - social, behavioral and mental health issues

THPDD0203: Quality and quantity: what do current experiences of older people with HIV tell us for future generations living into older age? Results from the UK 50 Plus study

Power L. 1 §
Brough G. 2
Bell M. 3
1 Terrence Higgins Trust, Policy & Public Affairs, London, United Kingdom
2 Terrence Higgins Trust, Membership & Involvement, London, United Kingdom
3 MBARC, London, United Kingdom
§ Presenting author email: lisa.power@tht.org.uk
Electronic publication date: 2012 Oct 22
Publication date: 2012
Volume: 15
Issue: Suppl 3
Electronic Location ID: 18442-084
Copyright: © 2012 L. Power et al.
Copyright year: 2012
License: This is an open-access article distributed under the terms of the Creative Commons Attribution License, which permits unrestricted use, distribution, and reproduction in any medium, provided the original work is properly cited.
License URL: http://creativecommons.org/licenses/by/2.0/

JOURNAL INFORMATION
==============================
NLM Title Abbreviation: J Int AIDS Soc
Journal ID: J Int AIDS Soc
NLM Journal ID: 101478566
Journal ID: JIAS

Journal of the International AIDS Society

EISSN: 1758-2652
Publisher: Wiley

ARTICLE INFORMATION
==============================
Article ID: 18442-085
Subjects: D53 - Ageing with HIV - social, behavioral and mental health issues

THPDD0206: Same old problem? Differing characteristics between heterosexual older adults with HIV who are white (HW) or from BME community (HBME)

Partridge N. 1 §
Stuart A. 2
Bell M. 2
Power L. 1
1 Terrence Higgins Trust, London, United Kingdom
2 MBARC, London, United Kingdom
§ Presenting author email: nick.partridge@tht.org.uk
Electronic publication date: 2012 Oct 22
Publication date: 2012
Volume: 15
Issue: Suppl 3
Electronic Location ID: 18442-085
Copyright: © 2012 N. Partridge et al.
Copyright year: 2012
License: This is an open-access article distributed under the terms of the Creative Commons Attribution License, which permits unrestricted use, distribution, and reproduction in any medium, provided the original work is properly cited.
License URL: http://creativecommons.org/licenses/by/2.0/

JOURNAL INFORMATION
==============================
NLM Title Abbreviation: J Int AIDS Soc
Journal ID: J Int AIDS Soc
NLM Journal ID: 101478566
Journal ID: JIAS

Journal of the International AIDS Society

EISSN: 1758-2652
Publisher: Wiley

ARTICLE INFORMATION
==============================
Article ID: 18442-086
Subjects: D56 - Improving social and behavioural data collection, triangulation of data sources and analysis in HIV research and programmes

MOAD0304: Who really provides care and support? The roles of young people in skipped-generation households

Reijer D. 1 2 §
1 AIDS Healthcare Foundation, Amsterdam, Netherlands
2 University of Amsterdam, Amsterdam, Netherlands
§ Presenting author email: daniel.reijer@aidshealth.org
Electronic publication date: 2012 Oct 22
Publication date: 2012
Volume: 15
Issue: Suppl 3
Electronic Location ID: 18442-086
Copyright: © 2012 D. Reijer et al.
Copyright year: 2012
License: This is an open-access article distributed under the terms of the Creative Commons Attribution License, which permits unrestricted use, distribution, and reproduction in any medium, provided the original work is properly cited.
License URL: http://creativecommons.org/licenses/by/2.0/

JOURNAL INFORMATION
==============================
NLM Title Abbreviation: J Int AIDS Soc
Journal ID: J Int AIDS Soc
NLM Journal ID: 101478566
Journal ID: JIAS

Journal of the International AIDS Society

EISSN: 1758-2652
Publisher: Wiley

ARTICLE INFORMATION
==============================
Article ID: 18442-087
Subjects: D68 - Available capacity, capacity building and organizational development

TUAD0201: Conducting an objective assessment of organizational capacity of NGOs providing services to HIV orphans and vulnerable children in India

Bryant M. 1 2
Bhatia A. 1
Mann C. 1 §
1 Boston University Center for Global Health and Development, Boston, United States
2 Boston University School of Public Health, International Health, Boston, United States
M. Bryant,
Electronic publication date: 2012 Oct 22
Publication date: 2012
Volume: 15
Issue: Suppl 3
Electronic Location ID: 18442-087
Copyright: © 2012 M. Bryant et al.
Copyright year: 2012
License: This is an open-access article distributed under the terms of the Creative Commons Attribution License, which permits unrestricted use, distribution, and reproduction in any medium, provided the original work is properly cited.
License URL: http://creativecommons.org/licenses/by/2.0/

JOURNAL INFORMATION
==============================
NLM Title Abbreviation: J Int AIDS Soc
Journal ID: J Int AIDS Soc
NLM Journal ID: 101478566
Journal ID: JIAS

Journal of the International AIDS Society

EISSN: 1758-2652
Publisher: Wiley

ARTICLE INFORMATION
==============================
Article ID: 18442-088
Subjects: D71 - Stigma and discrimination in health care settings

THAD0102: An intervention to reduce HIV stigma in health care settings in China

Li L. 1
Wu Z. 2
Liang L.-J. 1
Lin C. 1
Guan J. 3
Jia M. 4
Rou K. 2 §
1 UCLA Semel Institute, Los Angeles, United States
2 China CDC, Beijing, China
3 Fujian Provicial CDC, Fuzhou, China
4 Yunnan Provincial CDC, Kunming, China
L. Li,
Electronic publication date: 2012 Oct 22
Publication date: 2012
Volume: 15
Issue: Suppl 3
Electronic Location ID: 18442-088
Copyright: © 2012 L. Li et al.
Copyright year: 2012
License: This is an open-access article distributed under the terms of the Creative Commons Attribution License, which permits unrestricted use, distribution, and reproduction in any medium, provided the original work is properly cited.
License URL: http://creativecommons.org/licenses/by/2.0/

JOURNAL INFORMATION
==============================
NLM Title Abbreviation: J Int AIDS Soc
Journal ID: J Int AIDS Soc
NLM Journal ID: 101478566
Journal ID: JIAS

Journal of the International AIDS Society

EISSN: 1758-2652
Publisher: Wiley

ARTICLE INFORMATION
==============================
Article ID: 18442-089
Subjects: D71 - Stigma and discrimination in health care settings

THAD0104: Reducing stigma among Indian nursing students: a brief intervention involving local PLHIV networks

Ekstrand M.L. 1
Bharat S. 2
Ramakrishna J. 3
Shah S. 1
Perumpil S. 4
Srinivasan K. 5 §
1 University of California, San Francisco, Medicine, San Francisco, United States
2 Tata Institute of Social Sciences, Mumbai, India
3 National Institute of Mental Health and Neurosciences, Health Education, Bengaluru, India
4 St John's National Academy of Health Sciences, Nursing, Bengaluru, India
5 St John's National Academy of Health Sciences, St John's Research Institute, Bengaluru, India
M.L. Ekstrand,
Electronic publication date: 2012 Oct 22
Publication date: 2012
Volume: 15
Issue: Suppl 3
Electronic Location ID: 18442-089
Copyright: © 2012 M.L. Ekstrand et al.
Copyright year: 2012
License: This is an open-access article distributed under the terms of the Creative Commons Attribution License, which permits unrestricted use, distribution, and reproduction in any medium, provided the original work is properly cited.
License URL: http://creativecommons.org/licenses/by/2.0/

JOURNAL INFORMATION
==============================
NLM Title Abbreviation: J Int AIDS Soc
Journal ID: J Int AIDS Soc
NLM Journal ID: 101478566
Journal ID: JIAS

Journal of the International AIDS Society

EISSN: 1758-2652
Publisher: Wiley

ARTICLE INFORMATION
==============================
Article ID: 18442-090
Subjects: D71 - Stigma and discrimination in health care settings

THAD0105: Gender differences in HIV-related stigma among doctors in Egypt

Benkirane M. 1
Lohiniva A.-L. 1
Abdelrahman E. 2
Kamal W. 2
Talaat M. 1
1 U.S Naval Medical Research Unit No 3, Global Disease Detection and Response Program, Cairo, Egypt
2 Ministry of Health, National AIDS Program, Cairo, Egypt
M. Benkirane,
Electronic publication date: 2012 Oct 22
Publication date: 2012
Volume: 15
Issue: Suppl 3
Electronic Location ID: 18442-090
Copyright: © 2012 M. Benkirane et al.
Copyright year: 2012
License: This is an open-access article distributed under the terms of the Creative Commons Attribution License, which permits unrestricted use, distribution, and reproduction in any medium, provided the original work is properly cited.
License URL: http://creativecommons.org/licenses/by/2.0/

JOURNAL INFORMATION
==============================
NLM Title Abbreviation: J Int AIDS Soc
Journal ID: J Int AIDS Soc
NLM Journal ID: 101478566
Journal ID: JIAS

Journal of the International AIDS Society

EISSN: 1758-2652
Publisher: Wiley

ARTICLE INFORMATION
==============================
Article ID: 18442-091
Subjects: D71 - Stigma and discrimination in health care settings

THAD0106: Health worker perceptions on the impact of HIV-related stigma and discrimination on health service delivery in Kenya

Mugo L. 1
Chirchir B. 2
Njuguna P. 2
Kirui M. 2
Kinuthia P. 3
Ombam R. 3 §
1 Bon Sante Consulting Limited, Research, Nairobi, Kenya
2 Bon Sante Consulting Limited, Nairobi, Kenya
3 National AIDS Control Council Kenya, Nairobi, Kenya
L. Mugo,
Electronic publication date: 2012 Oct 22
Publication date: 2012
Volume: 15
Issue: Suppl 3
Electronic Location ID: 18442-091
Copyright: © 2012 L. Mugo et al.
Copyright year: 2012
License: This is an open-access article distributed under the terms of the Creative Commons Attribution License, which permits unrestricted use, distribution, and reproduction in any medium, provided the original work is properly cited.
License URL: http://creativecommons.org/licenses/by/2.0/

JOURNAL INFORMATION
==============================
NLM Title Abbreviation: J Int AIDS Soc
Journal ID: J Int AIDS Soc
NLM Journal ID: 101478566
Journal ID: JIAS

Journal of the International AIDS Society

EISSN: 1758-2652
Publisher: Wiley

ARTICLE INFORMATION
==============================
Article ID: 18442-092
Subjects: D72 - Family-centered approaches

MOPDD0103: Replication of the MAMA+model to prevent child abandonment by HIV-positive women in Yekaterinburg, Russia

Yorick R. 1 2
Suvorova S. 2
Sukovatova O. 1 2
Buchelnikova L. 3
Shutova M. 3
Melyakh A. 3
Hodgdon S. 4 §
1 HealthRight International, Representative Office in the Russian Federation, St. Petersburg, Russian Federation
2 Doctors to Children, St. Petersburg, Russian Federation
3 Family to Children, Yekaterinburg, Russian Federation
4 HealthRight International, New York, United States
O. Sukovatova,
Electronic publication date: 2012 Oct 22
Publication date: 2012
Volume: 15
Issue: Suppl 3
Electronic Location ID: 18442-092
Copyright: © 2012 O. Sukovatova et al.
Copyright year: 2012
License: This is an open-access article distributed under the terms of the Creative Commons Attribution License, which permits unrestricted use, distribution, and reproduction in any medium, provided the original work is properly cited.
License URL: http://creativecommons.org/licenses/by/2.0/

JOURNAL INFORMATION
==============================
NLM Title Abbreviation: J Int AIDS Soc
Journal ID: J Int AIDS Soc
NLM Journal ID: 101478566
Journal ID: JIAS

Journal of the International AIDS Society

EISSN: 1758-2652
Publisher: Wiley

ARTICLE INFORMATION
==============================
Article ID: 18442-093
Subjects: D72 - Family-centered approaches

MOPDD0104: Improving child-parent relationships among HIV+ mothers

Harutyunyan A. 1 §
Grigoryan M. 1
Zaqaryan N. 1
1 Positive People Armenian Network-Soc. NGO, Yerevan, Armenia
§ Presenting author . Email: ppan777@gmail.com
Electronic publication date: 2012 Oct 22
Publication date: 2012
Volume: 15
Issue: Suppl 3
Electronic Location ID: 18442-093
Copyright: © 2012 A. Harutyunyan et al.
Copyright year: 2012
License: This is an open-access article distributed under the terms of the Creative Commons Attribution License, which permits unrestricted use, distribution, and reproduction in any medium, provided the original work is properly cited.
License URL: http://creativecommons.org/licenses/by/2.0/

JOURNAL INFORMATION
==============================
NLM Title Abbreviation: J Int AIDS Soc
Journal ID: J Int AIDS Soc
NLM Journal ID: 101478566
Journal ID: JIAS

Journal of the International AIDS Society

EISSN: 1758-2652
Publisher: Wiley

ARTICLE INFORMATION
==============================
Article ID: 18442-094
Subjects: D74 - Community involvement and outreach in service delivery

TUAD0202: Strengthening treatment, care and support to people living with HIV through community-based treatment services

Dewo Z. 1
Tollera G. 1 §
Trivelli M. 2
Gebremariam M. 3
1 Ethiopian Catholic Secretariat, Addis Ababa, Ethiopia
2 St. Luke's Catholic hospital, Ethiopia, Wolisso, Ethiopia
3 Futures Group International, Addis Ababa, Ethiopia
§ Presenting author . Email: gtollera@gmial.com
Electronic publication date: 2012 Oct 22
Publication date: 2012
Volume: 15
Issue: Suppl 3
Electronic Location ID: 18442-094
Copyright: © 2012 G. Tollera et al.
Copyright year: 2012
License: This is an open-access article distributed under the terms of the Creative Commons Attribution License, which permits unrestricted use, distribution, and reproduction in any medium, provided the original work is properly cited.
License URL: http://creativecommons.org/licenses/by/2.0/

JOURNAL INFORMATION
==============================
NLM Title Abbreviation: J Int AIDS Soc
Journal ID: J Int AIDS Soc
NLM Journal ID: 101478566
Journal ID: JIAS

Journal of the International AIDS Society

EISSN: 1758-2652
Publisher: Wiley

ARTICLE INFORMATION
==============================
Article ID: 18442-095
Subjects: D74 - Community involvement and outreach in service delivery

TUAD0203: Community support systems: from horizontal initiative to vertical institution

Wagner A. 1 §
1 University of Hildesheim, Hildesheim, Germany
§ Presenting author . Email: andreas.wagner@uni-hildesheim.de
Electronic publication date: 2012 Oct 22
Publication date: 2012
Volume: 15
Issue: Suppl 3
Electronic Location ID: 18442-095
Copyright: © 2012 A. Wagner et al.
Copyright year: 2012
License: This is an open-access article distributed under the terms of the Creative Commons Attribution License, which permits unrestricted use, distribution, and reproduction in any medium, provided the original work is properly cited.
License URL: http://creativecommons.org/licenses/by/2.0/

JOURNAL INFORMATION
==============================
NLM Title Abbreviation: J Int AIDS Soc
Journal ID: J Int AIDS Soc
NLM Journal ID: 101478566
Journal ID: JIAS

Journal of the International AIDS Society

EISSN: 1758-2652
Publisher: Wiley

ARTICLE INFORMATION
==============================
Article ID: 18442-096
Subjects: D74 - Community involvement and outreach in service delivery

TUAD0204: Engagement of community volunteers for improving access to TB and HIV prevention, treatment and care services in Ibadan, Nigeria: the PLAN Foundation/TB CARE I experience

Oladapo O.O. 1 2 §
Akinyosoye S.A. 1 3
Ayoola J.K. 4
Omoniyi A.F. 5
van der Grinten E. 6
1 PLAN Health Advocacy and Development Foundation, Programs, Ibadan, Nigeria
2 Civil Society for the Eradication of Tuberculosis in Nigeria (TB Network-Nigeria), National Secretariat, Abuja, Nigeria
3 Civil Society for the Eradication of Tuberculosis in Nigeria (TB Network-Nigeria), Oyo State Secretariat, Ibadan, Nigeria
4 PLAN Health Advocacy and Development Foundation, Finance and Administration, Ibadan, Nigeria
5 World Health Organization (WHO), TB/HIV Department, Abuja, Nigeria
6 KNCV/TB CARE I, Country Office, Abuja, Nigeria
§ Presenting author . Email: obatunde65@gmail.com
Electronic publication date: 2012 Oct 22
Publication date: 2012
Volume: 15
Issue: Suppl 3
Electronic Location ID: 18442-096
Copyright: © 2012 O.O. Oladapo et al.
Copyright year: 2012
License: This is an open-access article distributed under the terms of the Creative Commons Attribution License, which permits unrestricted use, distribution, and reproduction in any medium, provided the original work is properly cited.
License URL: http://creativecommons.org/licenses/by/2.0/

JOURNAL INFORMATION
==============================
NLM Title Abbreviation: J Int AIDS Soc
Journal ID: J Int AIDS Soc
NLM Journal ID: 101478566
Journal ID: JIAS

Journal of the International AIDS Society

EISSN: 1758-2652
Publisher: Wiley

ARTICLE INFORMATION
==============================
Article ID: 18442-097
Subjects: D74 - Community involvement and outreach in service delivery

TUAD0205: Outreach clinic services for rural hard-to-reach people living with HIV (PLHIV): an approach to avoid follow-up default and sharing of ARVs

Mwakangalu D. 1 §
1 Pathfinder International, Kenya, Nairobi, Kenya
§ Presenting author email: dmwakangalu@pathfinder.org
Electronic publication date: 2012 Oct 22
Publication date: 2012
Volume: 15
Issue: Suppl 3
Electronic Location ID: 18442-097
Copyright: © 2012 D. Mwakangalu et al.
Copyright year: 2012
License: This is an open-access article distributed under the terms of the Creative Commons Attribution License, which permits unrestricted use, distribution, and reproduction in any medium, provided the original work is properly cited.
License URL: http://creativecommons.org/licenses/by/2.0/

JOURNAL INFORMATION
==============================
NLM Title Abbreviation: J Int AIDS Soc
Journal ID: J Int AIDS Soc
NLM Journal ID: 101478566
Journal ID: JIAS

Journal of the International AIDS Society

EISSN: 1758-2652
Publisher: Wiley

ARTICLE INFORMATION
==============================
Article ID: 18442-098
Subjects: D74 - Community involvement and outreach in service delivery

TUPDE0104: Community-led cash transfers in Zimbabwe: pathways for buy-in and improved child health and development

Skovdal M. 1 §
Robertson L. 2
Mushati P. 3
Nyamukapa C. 2 3
Sherr L. 4
Gregson S. 2 3
1 University of Bergen, Department of Health Promotion and Development, Bergen, Norway
2 School of Public Health, Imperial College London, London, United Kingdom
3 Biomedical Research and Training Institute, Harare, Zimbabwe
4 University College London, Department of Infection and Population Health, London, United Kingdom
§ Presenting author . Email: m.skovdal@gmail.com
Electronic publication date: 2012 Oct 22
Publication date: 2012
Volume: 15
Issue: Suppl 3
Electronic Location ID: 18442-098
Copyright: © 2012 M. Skovdal et al.
Copyright year: 2012
License: This is an open-access article distributed under the terms of the Creative Commons Attribution License, which permits unrestricted use, distribution, and reproduction in any medium, provided the original work is properly cited.
License URL: http://creativecommons.org/licenses/by/2.0/

JOURNAL INFORMATION
==============================
NLM Title Abbreviation: J Int AIDS Soc
Journal ID: J Int AIDS Soc
NLM Journal ID: 101478566
Journal ID: JIAS

Journal of the International AIDS Society

EISSN: 1758-2652
Publisher: Wiley

ARTICLE INFORMATION
==============================
Article ID: 18442-099
Subjects: D75 - Impact of service delivery on providers, coping strategies, burn-out and motivation

THAD0101: Treating the Nation: the positive impact of antiretroviral drugs on the clinical practice, coping strategies and workplace morale of health care workers in Kabarole District, Uganda

Belton S. 1 §
1 London School of Economics & Political Science, Institute of Social Psychology, London, United Kingdom
§ Presenting author . Email: s.k.belton@lse.ac.uk
Electronic publication date: 2012 Oct 22
Publication date: 2012
Volume: 15
Issue: Suppl 3
Electronic Location ID: 18442-099
Copyright: © 2012 S. Belton et al.
Copyright year: 2012
License: This is an open-access article distributed under the terms of the Creative Commons Attribution License, which permits unrestricted use, distribution, and reproduction in any medium, provided the original work is properly cited.
License URL: http://creativecommons.org/licenses/by/2.0/

JOURNAL INFORMATION
==============================
NLM Title Abbreviation: J Int AIDS Soc
Journal ID: J Int AIDS Soc
NLM Journal ID: 101478566
Journal ID: JIAS

Journal of the International AIDS Society

EISSN: 1758-2652
Publisher: Wiley

ARTICLE INFORMATION
==============================
Article ID: 18442-100
Subjects: D76 - Social factors determining use of HIV and AIDS, tuberculosis (TB), hepatitis and other related services

THAD0103: The debates over Mexico City's HIV/AIDS clinic: socio-political implications for HIV prevention, treatment and comprehensive health care for transgender sex workers

Gómez-Ramírez O. 1
1 University of British Columbia, Anthropology/Liu Institute for Global Issues, Vancouver, Canada
O. Gómez-Ramírez,
Electronic publication date: 2012 Oct 22
Publication date: 2012
Volume: 15
Issue: Suppl 3
Electronic Location ID: 18442-100
Copyright: © 2012 O. Gómez-Ramírez et al.
Copyright year: 2012
License: This is an open-access article distributed under the terms of the Creative Commons Attribution License, which permits unrestricted use, distribution, and reproduction in any medium, provided the original work is properly cited.
License URL: http://creativecommons.org/licenses/by/2.0/

JOURNAL INFORMATION
==============================
NLM Title Abbreviation: J Int AIDS Soc
Journal ID: J Int AIDS Soc
NLM Journal ID: 101478566
Journal ID: JIAS

Journal of the International AIDS Society

EISSN: 1758-2652
Publisher: Wiley

ARTICLE INFORMATION
==============================
Article ID: 18442-101
Subjects: D78 - Policy determinants and constraints

WEAD0603: Applying public health law research methods to address legal barriers and facilitators to effective HIV prevention programs

Carr M. 1
Lehman J.S. 1
Purcell D.W. 1 §
1 Centers for Disease Control and Prevention, Division of HIV/AIDS Prevention, Atlanta, United States
§ Presenting author email: dpurcell@cdc.gov
Electronic publication date: 2012 Oct 22
Publication date: 2012
Volume: 15
Issue: Suppl 3
Electronic Location ID: 18442-101
Copyright: © 2012 D.W. Purcell et al.
Copyright year: 2012
License: This is an open-access article distributed under the terms of the Creative Commons Attribution License, which permits unrestricted use, distribution, and reproduction in any medium, provided the original work is properly cited.
License URL: http://creativecommons.org/licenses/by/2.0/

JOURNAL INFORMATION
==============================
NLM Title Abbreviation: J Int AIDS Soc
Journal ID: J Int AIDS Soc
NLM Journal ID: 101478566
Journal ID: JIAS

Journal of the International AIDS Society

EISSN: 1758-2652
Publisher: Wiley

ARTICLE INFORMATION
==============================
Article ID: 18442-102
Subjects: D78 - Policy determinants and constraints

THAD0402: Effective representation of PLHIV in decision making processes still an uphill journey in Malawi; why?

Kampango G. 1 §
Caswell G. 2
1 Malawi Network of People Living with HIV/AIDS (MANET+), Programmes, Lilongwe, Malawi
2 Global Network of PLHIV (GNP+), Cape Town, South Africa
§ Presenting author . Email: gkampango@yahoo.co.uk
Electronic publication date: 2012 Oct 22
Publication date: 2012
Volume: 15
Issue: Suppl 3
Electronic Location ID: 18442-102
Copyright: © 2012 G. Kampango et al.
Copyright year: 2012
License: This is an open-access article distributed under the terms of the Creative Commons Attribution License, which permits unrestricted use, distribution, and reproduction in any medium, provided the original work is properly cited.
License URL: http://creativecommons.org/licenses/by/2.0/

JOURNAL INFORMATION
==============================
NLM Title Abbreviation: J Int AIDS Soc
Journal ID: J Int AIDS Soc
NLM Journal ID: 101478566
Journal ID: JIAS

Journal of the International AIDS Society

EISSN: 1758-2652
Publisher: Wiley

ARTICLE INFORMATION
==============================
Article ID: 18442-103
Subjects: D79 - Advocacy and lobbying by civil society, affected communities and PLWHA

THAD0401: Participation of women with HIV in the national response to the HIV and AIDS VI in the departments of Chinandega, Managua, Masaya, Rivas, Leon and South Atlantic Autonomous Region (SAAA)

Aburto Hernández K.E. 1 §
1 Fondo de Población de las Naciones Unidas (UNFPA), Managua, Nicaragua
§ Presenting author . Email: aburtokarla@gmail.com
Electronic publication date: 2012 Oct 22
Publication date: 2012
Volume: 15
Issue: Suppl 3
Electronic Location ID: 18442-103
Copyright: © 2012 K.E. Aburto Hernández et al.
Copyright year: 2012
License: This is an open-access article distributed under the terms of the Creative Commons Attribution License, which permits unrestricted use, distribution, and reproduction in any medium, provided the original work is properly cited.
License URL: http://creativecommons.org/licenses/by/2.0/

JOURNAL INFORMATION
==============================
NLM Title Abbreviation: J Int AIDS Soc
Journal ID: J Int AIDS Soc
NLM Journal ID: 101478566
Journal ID: JIAS

Journal of the International AIDS Society

EISSN: 1758-2652
Publisher: Wiley

ARTICLE INFORMATION
==============================
Article ID: 18442-104
Subjects: D79 - Advocacy and lobbying by civil society, affected communities and PLWHA

THAD0403: Mainstreaming gender into the national Global Fund strategy in China: a case study from a grassroots women's NGO network

Yuan W. 1 §
1 Henan Women's Action Group, Zhengzhou, China
§ Presenting author . Email: yuanwenli2010@gmail.com
Electronic publication date: 2012 Oct 22
Publication date: 2012
Volume: 15
Issue: Suppl 3
Electronic Location ID: 18442-104
Copyright: © 2012 W. Yuan et al.
Copyright year: 2012
License: This is an open-access article distributed under the terms of the Creative Commons Attribution License, which permits unrestricted use, distribution, and reproduction in any medium, provided the original work is properly cited.
License URL: http://creativecommons.org/licenses/by/2.0/

JOURNAL INFORMATION
==============================
NLM Title Abbreviation: J Int AIDS Soc
Journal ID: J Int AIDS Soc
NLM Journal ID: 101478566
Journal ID: JIAS

Journal of the International AIDS Society

EISSN: 1758-2652
Publisher: Wiley

ARTICLE INFORMATION
==============================
Article ID: 18442-105
Subjects: D79 - Advocacy and lobbying by civil society, affected communities and PLWHA

THAD0405: Influencing the political arena: case studies of women living with HIV's meaningful participation in shaping HIV decision-making, policies and programs in Rwanda

Furaha Kalumire V. 1 §
Uwimpuhwe S. 2
Doyle K. 3
1 Rwandan Women Living with HIV/AIDS and Fighting Against It (FRSL+/RW), Legal Representative, Kigali, Rwanda
2 Rwanda Biomedical Center, Institute of HIV/AIDS, Disease Prevention and Control, Senior Advisor Gender Equality & HIV/AIDS, Kigali, Rwanda
3 UNAIDS Rwanda, Gender Focal Point, Kigali, Rwanda
§ Presenting author . Email: fkvivi@yahoo.fr
Electronic publication date: 2012 Oct 22
Publication date: 2012
Volume: 15
Issue: Suppl 3
Electronic Location ID: 18442-105
Copyright: © 2012 V. Furaha Kalumire et al.
Copyright year: 2012
License: This is an open-access article distributed under the terms of the Creative Commons Attribution License, which permits unrestricted use, distribution, and reproduction in any medium, provided the original work is properly cited.
License URL: http://creativecommons.org/licenses/by/2.0/

JOURNAL INFORMATION
==============================
NLM Title Abbreviation: J Int AIDS Soc
Journal ID: J Int AIDS Soc
NLM Journal ID: 101478566
Journal ID: JIAS

Journal of the International AIDS Society

EISSN: 1758-2652
Publisher: Wiley

ARTICLE INFORMATION
==============================
Article ID: 18442-106
Subjects: D79 - Advocacy and lobbying by civil society, affected communities and PLWHA

TUPDD0104: Ending discrimination in recruitment processes: an effective campaign to prohibit pre-employment questionnaires

Dunkeyson L. 1 §
Briggs E. 1
1 NAT (National AIDS Trust), London, United Kingdom
§ Presenting author . Email: laura.dunkeyson@nat.org.uk
Electronic publication date: 2012 Oct 22
Publication date: 2012
Volume: 15
Issue: Suppl 3
Electronic Location ID: 18442-106
Copyright: © 2012 L. Dunkeyson et al.
Copyright year: 2012
License: This is an open-access article distributed under the terms of the Creative Commons Attribution License, which permits unrestricted use, distribution, and reproduction in any medium, provided the original work is properly cited.
License URL: http://creativecommons.org/licenses/by/2.0/

JOURNAL INFORMATION
==============================
NLM Title Abbreviation: J Int AIDS Soc
Journal ID: J Int AIDS Soc
NLM Journal ID: 101478566
Journal ID: JIAS

Journal of the International AIDS Society

EISSN: 1758-2652
Publisher: Wiley

ARTICLE INFORMATION
==============================
Article ID: 18442-107
Subjects: D79 - Advocacy and lobbying by civil society, affected communities and PLWHA

TUPDD0105: AIDS and human rights activists fighting for maternal health justice in Uganda: the value of real synergies

Mulumba M. 1 §
Russell A. 2
Kwagalah P. 1
Musisi N.N. 1
1 Centre for Health, Human Rights and Development (CEHURD), Kampala, Uganda
2 Health GAP Global Access Project, New York, United States
§ Presenting author . Email: mulumbam@gmail.com
Electronic publication date: 2012 Oct 22
Publication date: 2012
Volume: 15
Issue: Suppl 3
Electronic Location ID: 18442-107
Copyright: © 2012 M. Mulumba et al.
Copyright year: 2012
License: This is an open-access article distributed under the terms of the Creative Commons Attribution License, which permits unrestricted use, distribution, and reproduction in any medium, provided the original work is properly cited.
License URL: http://creativecommons.org/licenses/by/2.0/

JOURNAL INFORMATION
==============================
NLM Title Abbreviation: J Int AIDS Soc
Journal ID: J Int AIDS Soc
NLM Journal ID: 101478566
Journal ID: JIAS

Journal of the International AIDS Society

EISSN: 1758-2652
Publisher: Wiley

ARTICLE INFORMATION
==============================
Article ID: 18442-108
Subjects: D80 - Evidence-informed policy development

MOAD0203: Evidence-based drug policy in four African countries: barriers and opportunities

Csete J. 1
1 Open Society Foundations, Global Drug Policy Program, London, United Kingdom
J. Csete,
Electronic publication date: 2012 Oct 22
Publication date: 2012
Volume: 15
Issue: Suppl 3
Electronic Location ID: 18442-108
Copyright: © 2012 J. Csete et al.
Copyright year: 2012
License: This is an open-access article distributed under the terms of the Creative Commons Attribution License, which permits unrestricted use, distribution, and reproduction in any medium, provided the original work is properly cited.
License URL: http://creativecommons.org/licenses/by/2.0/

JOURNAL INFORMATION
==============================
NLM Title Abbreviation: J Int AIDS Soc
Journal ID: J Int AIDS Soc
NLM Journal ID: 101478566
Journal ID: JIAS

Journal of the International AIDS Society

EISSN: 1758-2652
Publisher: Wiley

ARTICLE INFORMATION
==============================
Article ID: 18442-109
Subjects: D80 - Evidence-informed policy development

WEAD0604: Bottlenecks analysis: a critical step to evidence-based planning for eMTCT, Cameroon experience

Kanon S. 1 §
Baye M. 2
Bissek A.C. 2
Noba V. 1
Akondeng L. 1
Kembou E. 3
Ebogo M. 4
Kamenga C. 5
Oulare M. 5
Seye A. 5
Popotte F. 5
Tchendjou P. 2
Sakho L. 6
1 UNICEF, Yaounde, Cameroon
2 MoH Cameroon, Yaounde, Cameroon
3 WHO, Yaounde, Cameroon
4 NACC, Yaounde, Cameroon
5 UNICEF, Dakar, Senegal
6 UNAIDS, Yaounde, Cameroon
§ Presenting author . Email: skanon@unicef.org
Electronic publication date: 2012 Oct 22
Publication date: 2012
Volume: 15
Issue: Suppl 3
Electronic Location ID: 18442-109
Copyright: © 2012 S. Kanon et al.
Copyright year: 2012
License: This is an open-access article distributed under the terms of the Creative Commons Attribution License, which permits unrestricted use, distribution, and reproduction in any medium, provided the original work is properly cited.
License URL: http://creativecommons.org/licenses/by/2.0/

JOURNAL INFORMATION
==============================
NLM Title Abbreviation: J Int AIDS Soc
Journal ID: J Int AIDS Soc
NLM Journal ID: 101478566
Journal ID: JIAS

Journal of the International AIDS Society

EISSN: 1758-2652
Publisher: Wiley

ARTICLE INFORMATION
==============================
Article ID: 18442-110
Subjects: D80 - Evidence-informed policy development

THAD0404: Community-based actions and services achieve results

Rodriguez-Garcia R. 1
Kayode O. 2
Manteuffel B. 3
Simms B. 4
de Zalduondo B. 5
Bonnel R. 1
Njie N. 1
1 The World Bank, Washington, United States
2 NACA, Abuja, Nigeria
3 IFC Macro, Atlanta, United States
4 UK Consortium on AIDS and International Development, London, United Kingdom
5 UNAIDS, Department of Evidence, Geneva, Switzerland
R. Rodriguez-Garcia,
Electronic publication date: 2012 Oct 22
Publication date: 2012
Volume: 15
Issue: Suppl 3
Electronic Location ID: 18442-110
Copyright: © 2012 R. Rodriguez-Garcia et al.
Copyright year: 2012
License: This is an open-access article distributed under the terms of the Creative Commons Attribution License, which permits unrestricted use, distribution, and reproduction in any medium, provided the original work is properly cited.
License URL: http://creativecommons.org/licenses/by/2.0/

JOURNAL INFORMATION
==============================
NLM Title Abbreviation: J Int AIDS Soc
Journal ID: J Int AIDS Soc
NLM Journal ID: 101478566
Journal ID: JIAS

Journal of the International AIDS Society

EISSN: 1758-2652
Publisher: Wiley

ARTICLE INFORMATION
==============================
Article ID: 18442-111
Subjects: D83 - Histories of policy responses to the epidemic

WEPDD0206: International blood donation guidelines for men who have sex with men (MSM)

Schaefer N. 1
Valadéz R. 1 §
1 Gay Men's Health Crisis, Public Policy, New York, United States
N. Schaefer,
Electronic publication date: 2012 Oct 22
Publication date: 2012
Volume: 15
Issue: Suppl 3
Electronic Location ID: 18442-111
Copyright: © 2012 N. Schaefer et al.
Copyright year: 2012
License: This is an open-access article distributed under the terms of the Creative Commons Attribution License, which permits unrestricted use, distribution, and reproduction in any medium, provided the original work is properly cited.
License URL: http://creativecommons.org/licenses/by/2.0/

JOURNAL INFORMATION
==============================
NLM Title Abbreviation: J Int AIDS Soc
Journal ID: J Int AIDS Soc
NLM Journal ID: 101478566
Journal ID: JIAS

Journal of the International AIDS Society

EISSN: 1758-2652
Publisher: Wiley

ARTICLE INFORMATION
==============================
Article ID: 18442-112
Subjects: D84 - Policies regarding HIV prevention, diagnosis, treatment and care

WEPDD0201: Analysis of political governance as a determinant of level of ART coverage using country-level data

Man W.Y.N. 1
Worth H. 1
Wilson D. 2
Kelly A. 1 3
Siba P. 3
Wong Y. 4 §
1 University of New South Wales, International HIV Research Group, Sydney, Australia
2 University of New South Wales, Kirby Institute, Sydney, Australia
3 Papua New Guinea Institute of Medical Research, Goroka, Papua New Guinea
4 University of New South Wales, School of Public Health and Community Medicine, Sydney, Australia
H. Worth,
Electronic publication date: 2012 Oct 22
Publication date: 2012
Volume: 15
Issue: Suppl 3
Electronic Location ID: 18442-112
Copyright: © 2012 H. Worth et al.
Copyright year: 2012
License: This is an open-access article distributed under the terms of the Creative Commons Attribution License, which permits unrestricted use, distribution, and reproduction in any medium, provided the original work is properly cited.
License URL: http://creativecommons.org/licenses/by/2.0/

JOURNAL INFORMATION
==============================
NLM Title Abbreviation: J Int AIDS Soc
Journal ID: J Int AIDS Soc
NLM Journal ID: 101478566
Journal ID: JIAS

Journal of the International AIDS Society

EISSN: 1758-2652
Publisher: Wiley

ARTICLE INFORMATION
==============================
Article ID: 18442-113
Subjects: D84 - Policies regarding HIV prevention, diagnosis, treatment and care

WEPDD0205: Three I's for HIV/TB and early ART to prevent HIV and TB: policy review of HIV and TB guidelines for high HIV/TB-burden African countries

Gupta S.S. 1
Granich R. 1
Suthar A.B. 1
Smyth C. 1
Baggaley R. 1
Sculier D. 1
Date A. 2
Desai M.A. 2
Lule F. 3
Raizes E. 2
Blanc L. 1
McClure C. 1
Hirnschall G. 1 §
1 World Health Organization, Geneva, Switzerland
2 Centers for Disease Control and Prevention (CDC), Atlanta, United States
3 World Health Organization (WR Country Office), Brazzaville, Congo, Republic of the Congo
S.S. Gupta,
Electronic publication date: 2012 Oct 22
Publication date: 2012
Volume: 15
Issue: Suppl 3
Electronic Location ID: 18442-113
Copyright: © 2012 S.S. Gupta et al.
Copyright year: 2012
License: This is an open-access article distributed under the terms of the Creative Commons Attribution License, which permits unrestricted use, distribution, and reproduction in any medium, provided the original work is properly cited.
License URL: http://creativecommons.org/licenses/by/2.0/

JOURNAL INFORMATION
==============================
NLM Title Abbreviation: J Int AIDS Soc
Journal ID: J Int AIDS Soc
NLM Journal ID: 101478566
Journal ID: JIAS

Journal of the International AIDS Society

EISSN: 1758-2652
Publisher: Wiley

ARTICLE INFORMATION
==============================
Article ID: 18442-114
Subjects: D85 - Policies addressing social and economic determinants of vulnerability

WEPDD0202: UNAIDS’ gender policy: strengths and weaknesses

Olinyk S. 1
Gibbs A. 2 §
Campbell C. 3 4
1 University of Michigan, Ann Arbor, United States
2 University of KwaZulu-Natal, Health Economics and HIV/AIDS Research Division (HEARD), Durban, South Africa
3 London School of Economics, London, United Kingdom
4 University of KwaZulu-Natal, Durban, South Africa
§ Presenting author email: gibbs@ukzn.ac.za
Electronic publication date: 2012 Oct 22
Publication date: 2012
Volume: 15
Issue: Suppl 3
Electronic Location ID: 18442-114
Copyright: © 2012 A. Gibbs et al.
Copyright year: 2012
License: This is an open-access article distributed under the terms of the Creative Commons Attribution License, which permits unrestricted use, distribution, and reproduction in any medium, provided the original work is properly cited.
License URL: http://creativecommons.org/licenses/by/2.0/

JOURNAL INFORMATION
==============================
NLM Title Abbreviation: J Int AIDS Soc
Journal ID: J Int AIDS Soc
NLM Journal ID: 101478566
Journal ID: JIAS

Journal of the International AIDS Society

EISSN: 1758-2652
Publisher: Wiley

ARTICLE INFORMATION
==============================
Article ID: 18442-115
Subjects: D86 - Policies addressing HIV and AIDS in the workplace and educational institutions

TUPDD0102: License denied: professional and vocational licensing restrictions affecting people living with HIV in the United States

Yager A. 1 §
1 HIV Law Project, New York, United States
§ Presenting author email: ayager@hivlawproject.org
Electronic publication date: 2012 Oct 22
Publication date: 2012
Volume: 15
Issue: Suppl 3
Electronic Location ID: 18442-115
Copyright: © 2012 A. Yager et al.
Copyright year: 2012
License: This is an open-access article distributed under the terms of the Creative Commons Attribution License, which permits unrestricted use, distribution, and reproduction in any medium, provided the original work is properly cited.
License URL: http://creativecommons.org/licenses/by/2.0/

JOURNAL INFORMATION
==============================
NLM Title Abbreviation: J Int AIDS Soc
Journal ID: J Int AIDS Soc
NLM Journal ID: 101478566
Journal ID: JIAS

Journal of the International AIDS Society

EISSN: 1758-2652
Publisher: Wiley

ARTICLE INFORMATION
==============================
Article ID: 18442-116
Subjects: D88 - Monitoring and evaluation of policies and their impact on PLWHA, affected communities and vulnerable populations

TUPDD0103: Southern exposure: HIV and human rights advocacy in the southern United States

McLemore M. 1 §
1 Human Rights Watch, Health and Human Rights, New York,, United States
§ Presenting author email: mclemom@hrw.org
Electronic publication date: 2012 Oct 22
Publication date: 2012
Volume: 15
Issue: Suppl 3
Electronic Location ID: 18442-116
Copyright: © 2012 M. McLemore et al.
Copyright year: 2012
License: This is an open-access article distributed under the terms of the Creative Commons Attribution License, which permits unrestricted use, distribution, and reproduction in any medium, provided the original work is properly cited.
License URL: http://creativecommons.org/licenses/by/2.0/

JOURNAL INFORMATION
==============================
NLM Title Abbreviation: J Int AIDS Soc
Journal ID: J Int AIDS Soc
NLM Journal ID: 101478566
Journal ID: JIAS

Journal of the International AIDS Society

EISSN: 1758-2652
Publisher: Wiley

ARTICLE INFORMATION
==============================
Article ID: 18442-117
Subjects: D89 - Impact of policies on health systems

MOAD0202: Overcoming double standard policies towards expansion of opioid substitution treatment (OST) in Ukraine

Lebega O. 1
Islam Z. 1
Filippovych S. 1
Talalayev K. 2
1 ICF International HIV/AIDS Alliance in Ukraine, TPSM, Kyiv, Ukraine
2 Freelance, Kyiv, Ukraine
O. Lebega,
Electronic publication date: 2012 Oct 22
Publication date: 2012
Volume: 15
Issue: Suppl 3
Electronic Location ID: 18442-117
Copyright: © 2012 O. Lebega et al.
Copyright year: 2012
License: This is an open-access article distributed under the terms of the Creative Commons Attribution License, which permits unrestricted use, distribution, and reproduction in any medium, provided the original work is properly cited.
License URL: http://creativecommons.org/licenses/by/2.0/

JOURNAL INFORMATION
==============================
NLM Title Abbreviation: J Int AIDS Soc
Journal ID: J Int AIDS Soc
NLM Journal ID: 101478566
Journal ID: JIAS

Journal of the International AIDS Society

EISSN: 1758-2652
Publisher: Wiley

ARTICLE INFORMATION
==============================
Article ID: 18442-118
Subjects: D89 - Impact of policies on health systems

WEPDD0204: Complementary production of global public goods for health: measuring contributions of HIV/AIDS initiatives

Nixon Thompson J.A. 1 2 §
1 University of Maryland, College Park, Sociology, College Park, United States
2 International Institute for Applied Systems Analysis, Health & Global Change, Laxenburg, Austria
§ Presenting author email: jalicenixon@gmail.com
Electronic publication date: 2012 Oct 22
Publication date: 2012
Volume: 15
Issue: Suppl 3
Electronic Location ID: 18442-118
Copyright: © 2012 J.A. Nixon Thompson et al.
Copyright year: 2012
License: This is an open-access article distributed under the terms of the Creative Commons Attribution License, which permits unrestricted use, distribution, and reproduction in any medium, provided the original work is properly cited.
License URL: http://creativecommons.org/licenses/by/2.0/

JOURNAL INFORMATION
==============================
NLM Title Abbreviation: J Int AIDS Soc
Journal ID: J Int AIDS Soc
NLM Journal ID: 101478566
Journal ID: JIAS

Journal of the International AIDS Society

EISSN: 1758-2652
Publisher: Wiley

ARTICLE INFORMATION
==============================
Article ID: 18442-119
Subjects: D91 - Analysis of resource allocation and monitoring of their use

WEAD0601: Mapping HIV spending in Central America: providing evidence for a renewed policy agenda

Valladares Cardona R.D.
Guatemala NASA Teams
Belize
El Salvador
Nicaragua
Costa Rica
Panama
USAID/PASCA, Monitoring and Evaluation Strengthening in CA, Guatemala, Guatemala
§ Presenting author email: rvalladares@pasca.org.gt
Electronic publication date: 2012 Oct 22
Publication date: 2012
Volume: 15
Issue: Suppl 3
Electronic Location ID: 18442-119
Copyright: © 2012 R.D. Valladares Cardona et al.
Copyright year: 2012
License: This is an open-access article distributed under the terms of the Creative Commons Attribution License, which permits unrestricted use, distribution, and reproduction in any medium, provided the original work is properly cited.
License URL: http://creativecommons.org/licenses/by/2.0/

JOURNAL INFORMATION
==============================
NLM Title Abbreviation: J Int AIDS Soc
Journal ID: J Int AIDS Soc
NLM Journal ID: 101478566
Journal ID: JIAS

Journal of the International AIDS Society

EISSN: 1758-2652
Publisher: Wiley

ARTICLE INFORMATION
==============================
Article ID: 18442-120
Subjects: D91 - Analysis of resource allocation and monitoring of their use

WEAD0602: Using geographic information systems mapping as a decision support tool for PEPFAR South Africa

Kunaka D. 1 §
Mashamba M. 2
1 John Snow Incorporated, Pretoria, South Africa
2 USAID South Africa, Strategic Information, Pretoria, South Africa
§ Presenting author email: derek@enhancesi.co.za
Electronic publication date: 2012 Oct 22
Publication date: 2012
Volume: 15
Issue: Suppl 3
Electronic Location ID: 18442-120
Copyright: © 2012 D. Kunaka et al.
Copyright year: 2012
License: This is an open-access article distributed under the terms of the Creative Commons Attribution License, which permits unrestricted use, distribution, and reproduction in any medium, provided the original work is properly cited.
License URL: http://creativecommons.org/licenses/by/2.0/

JOURNAL INFORMATION
==============================
NLM Title Abbreviation: J Int AIDS Soc
Journal ID: J Int AIDS Soc
NLM Journal ID: 101478566
Journal ID: JIAS

Journal of the International AIDS Society

EISSN: 1758-2652
Publisher: Wiley

ARTICLE INFORMATION
==============================
Article ID: 18442-121
Subjects: D91 - Analysis of resource allocation and monitoring of their use

WEPDD0203: Investing in HIV prevention in a global recession: HIV prevention research and development funding trends 2000–2011

Fisher K. 1 §
Donaldson E. 1
Gobet B. 2
Green L.M. 3
Harmon T. 4
Harrison P. 1
Lande R. 3
Warren M. 1
1 AVAC, New York, United States
2 UNAIDS, the Joint United Nations Programme on HIV/AIDS, Geneva, Switzerland
3 International Partnership for Microbicides, Silver Spring, United States
4 International AIDS Vaccine Initiative, New York, United States
E. Donaldson,
Electronic publication date: 2012 Oct 22
Publication date: 2012
Volume: 15
Issue: Suppl 3
Electronic Location ID: 18442-121
Copyright: © 2012 E. Donaldson et al.
Copyright year: 2012
License: This is an open-access article distributed under the terms of the Creative Commons Attribution License, which permits unrestricted use, distribution, and reproduction in any medium, provided the original work is properly cited.
License URL: http://creativecommons.org/licenses/by/2.0/

JOURNAL INFORMATION
==============================
NLM Title Abbreviation: J Int AIDS Soc
Journal ID: J Int AIDS Soc
NLM Journal ID: 101478566
Journal ID: JIAS

Journal of the International AIDS Society

EISSN: 1758-2652
Publisher: Wiley

ARTICLE INFORMATION
==============================
Article ID: 18442-122
Subjects: D92 - Impact of the law, law enforcement and/or access to justice on the HIV response

TUAD0101: Reducing conflicts between public security policy and HIV response: a research of the impact of prostitution elimination policy on female sex workers

Zhai W. 1 §
1 AIDS Care China, Kunming, China
§ Presenting author email: zhaiwen2@gmail.com
Electronic publication date: 2012 Oct 22
Publication date: 2012
Volume: 15
Issue: Suppl 3
Electronic Location ID: 18442-122
Copyright: © 2012 W. Zhai et al.
Copyright year: 2012
License: This is an open-access article distributed under the terms of the Creative Commons Attribution License, which permits unrestricted use, distribution, and reproduction in any medium, provided the original work is properly cited.
License URL: http://creativecommons.org/licenses/by/2.0/

JOURNAL INFORMATION
==============================
NLM Title Abbreviation: J Int AIDS Soc
Journal ID: J Int AIDS Soc
NLM Journal ID: 101478566
Journal ID: JIAS

Journal of the International AIDS Society

EISSN: 1758-2652
Publisher: Wiley

ARTICLE INFORMATION
==============================
Article ID: 18442-123
Subjects: D92 - Impact of the law, law enforcement and/or access to justice on the HIV response

THAD0202: Eight-country study of three-year program to strengthen and expand HIV-related legal services for people living with HIV and key affected populations: what did we learn about scale-up?

Burke-Shyne N. 1 §
Patterson D. 1
Guilland R. 1
Kalugampitiya A. 1
Meite N. 1
Perez O.L. 1
Nardicchia S. 1
1 International Development Law Organization, Social Development Programs Unit, Rome, Italy
§ Presenting author email: nburkeshyne@idlo.int
Electronic publication date: 2012 Oct 22
Publication date: 2012
Volume: 15
Issue: Suppl 3
Electronic Location ID: 18442-123
Copyright: © 2012 N. Burke-Shyne et al.
Copyright year: 2012
License: This is an open-access article distributed under the terms of the Creative Commons Attribution License, which permits unrestricted use, distribution, and reproduction in any medium, provided the original work is properly cited.
License URL: http://creativecommons.org/licenses/by/2.0/

JOURNAL INFORMATION
==============================
NLM Title Abbreviation: J Int AIDS Soc
Journal ID: J Int AIDS Soc
NLM Journal ID: 101478566
Journal ID: JIAS

Journal of the International AIDS Society

EISSN: 1758-2652
Publisher: Wiley

ARTICLE INFORMATION
==============================
Article ID: 18442-124
Subjects: D92 - Impact of the law, law enforcement and/or access to justice on the HIV response

MOPDD0201: Are we listening? What sex workers know about prostitution policy and risk of HIV

McCracken J. 1 §
Clay C. 2
Dorsey K. 3
Coplen L. 4
Libertine S. 5
1 University of South Florida St. Petersburg, Verbal and Visual Arts, St. Petersburg, United States
2 HIPS, Washington, United States
3 Different Avenues, Washington, United States
4 SWOP-USA, Tucson, United States
5 SWOP-Chicago, Chicago, United States
§ Presenting author email: mccracken.jill@gmail.com
Electronic publication date: 2012 Oct 22
Publication date: 2012
Volume: 15
Issue: Suppl 3
Electronic Location ID: 18442-124
Copyright: © 2012 J. McCracken et al.
Copyright year: 2012
License: This is an open-access article distributed under the terms of the Creative Commons Attribution License, which permits unrestricted use, distribution, and reproduction in any medium, provided the original work is properly cited.
License URL: http://creativecommons.org/licenses/by/2.0/

JOURNAL INFORMATION
==============================
NLM Title Abbreviation: J Int AIDS Soc
Journal ID: J Int AIDS Soc
NLM Journal ID: 101478566
Journal ID: JIAS

Journal of the International AIDS Society

EISSN: 1758-2652
Publisher: Wiley

ARTICLE INFORMATION
==============================
Article ID: 18442-125
Subjects: D92 - Impact of the law, law enforcement and/or access to justice on the HIV response

MOPDD0204: Criminalizing condoms: how policing practices put sex workers and HIV services at risk

Shields A. 1 §
Thomas R. 1
Hahn S. 1
Weidmann J. 1
1 Open Society Foundations, Sexual Health and Rights Project, New York, United States
§ Presenting author email: acacia.shields@gmail.com
Electronic publication date: 2012 Oct 22
Publication date: 2012
Volume: 15
Issue: Suppl 3
Electronic Location ID: 18442-125
Copyright: © 2012 A. Shields et al.
Copyright year: 2012
License: This is an open-access article distributed under the terms of the Creative Commons Attribution License, which permits unrestricted use, distribution, and reproduction in any medium, provided the original work is properly cited.
License URL: http://creativecommons.org/licenses/by/2.0/

JOURNAL INFORMATION
==============================
NLM Title Abbreviation: J Int AIDS Soc
Journal ID: J Int AIDS Soc
NLM Journal ID: 101478566
Journal ID: JIAS

Journal of the International AIDS Society

EISSN: 1758-2652
Publisher: Wiley

ARTICLE INFORMATION
==============================
Article ID: 18442-126
Subjects: D92 - Impact of the law, law enforcement and/or access to justice on the HIV response

MOPDD0206: The legal framework of sex work and its interface with HIV: barriers for a sustainable response to HIV programming in Bangladesh

Gomes T.M. 1 §
Akter M.H. 2
Begum S. 3
Rasin S. 1
1 Save the Children, HIV AIDS - Advocacy and Social Mobilization, Dhaka, Bangladesh
2 Sex Workers Network of Bangladesh, Dhaka, Bangladesh
3 Durjoy Nari Shongo, Sex Workers Network, Dhaka, Bangladesh
S. Rasin,
Electronic publication date: 2012 Oct 22
Publication date: 2012
Volume: 15
Issue: Suppl 3
Electronic Location ID: 18442-126
Copyright: © 2012 S. Rasin et al.
Copyright year: 2012
License: This is an open-access article distributed under the terms of the Creative Commons Attribution License, which permits unrestricted use, distribution, and reproduction in any medium, provided the original work is properly cited.
License URL: http://creativecommons.org/licenses/by/2.0/

JOURNAL INFORMATION
==============================
NLM Title Abbreviation: J Int AIDS Soc
Journal ID: J Int AIDS Soc
NLM Journal ID: 101478566
Journal ID: JIAS

Journal of the International AIDS Society

EISSN: 1758-2652
Publisher: Wiley

ARTICLE INFORMATION
==============================
Article ID: 18442-127
Subjects: D93 - Intellectual property and trade regimes and access to HIV treatment and diagnostic medical devices

WEAD0401: Killing the Doha Declaration and access to ARVs: one free trade agreement (FTA) at a time

Bhardwaj K. 1
1 Independent Lawyer (HIV, Health and Human Rights), New Delhi, India
K. Bhardwaj,
Electronic publication date: 2012 Oct 22
Publication date: 2012
Volume: 15
Issue: Suppl 3
Electronic Location ID: 18442-127
Copyright: © 2012 K. Bhardwaj et al.
Copyright year: 2012
License: This is an open-access article distributed under the terms of the Creative Commons Attribution License, which permits unrestricted use, distribution, and reproduction in any medium, provided the original work is properly cited.
License URL: http://creativecommons.org/licenses/by/2.0/

JOURNAL INFORMATION
==============================
NLM Title Abbreviation: J Int AIDS Soc
Journal ID: J Int AIDS Soc
NLM Journal ID: 101478566
Journal ID: JIAS

Journal of the International AIDS Society

EISSN: 1758-2652
Publisher: Wiley

ARTICLE INFORMATION
==============================
Article ID: 18442-128
Subjects: D93 - Intellectual property and trade regimes and access to HIV treatment and diagnostic medical devices

WEAD0402: ARV patents on the rise? An analysis of ARV patent status in 69 low- and middle-income countries

Burrone E. 1 §
Boulet P. 2
Moon S. 3
Park C. 1
't Hoen E. 1
Shen J. 4
Sunderji N. 5
1 Medicines Patent Pool, Geneva, Switzerland
2 Independent Consultant, Geneva, Switzerland
3 Harvard University, Boston, United States
4 Accenture, London, United Kingdom
5 Independent Consultant, Boston, United States
§ Presenting author email: eburrone@medicinespatentpool.org
Electronic publication date: 2012 Oct 22
Publication date: 2012
Volume: 15
Issue: Suppl 3
Electronic Location ID: 18442-128
Copyright: © 2012 E. Burrone et al.
Copyright year: 2012
License: This is an open-access article distributed under the terms of the Creative Commons Attribution License, which permits unrestricted use, distribution, and reproduction in any medium, provided the original work is properly cited.
License URL: http://creativecommons.org/licenses/by/2.0/

JOURNAL INFORMATION
==============================
NLM Title Abbreviation: J Int AIDS Soc
Journal ID: J Int AIDS Soc
NLM Journal ID: 101478566
Journal ID: JIAS

Journal of the International AIDS Society

EISSN: 1758-2652
Publisher: Wiley

ARTICLE INFORMATION
==============================
Article ID: 18442-129
Subjects: D93 - Intellectual property and trade regimes and access to HIV treatment and diagnostic medical devices

WEAD0403: Intellectual property rights and access to HIV/AIDS medicines in french speaking African countries: issues, problems and prospects

Kameni E. 1
1 University of Pretoria, Faculty of Law, Pretoria, South Africa
E. Kameni,
Electronic publication date: 2012 Oct 22
Publication date: 2012
Volume: 15
Issue: Suppl 3
Electronic Location ID: 18442-129
Copyright: © 2012 E. Kameni et al.
Copyright year: 2012
License: This is an open-access article distributed under the terms of the Creative Commons Attribution License, which permits unrestricted use, distribution, and reproduction in any medium, provided the original work is properly cited.
License URL: http://creativecommons.org/licenses/by/2.0/

JOURNAL INFORMATION
==============================
NLM Title Abbreviation: J Int AIDS Soc
Journal ID: J Int AIDS Soc
NLM Journal ID: 101478566
Journal ID: JIAS

Journal of the International AIDS Society

EISSN: 1758-2652
Publisher: Wiley

ARTICLE INFORMATION
==============================
Article ID: 18442-130
Subjects: D93 - Intellectual property and trade regimes and access to HIV treatment and diagnostic medical devices

WEAD0404: Impact of anti-counterfeiting measures on access to generic ARVs in developing countries

Metheny N. 1 §
1 Thai AIDS Treatment Action Group, Treatment Access Legal Advocate, Bangkok, Thailand
§ Presenting author email: nmetheny@hsph.harvard.edu
Electronic publication date: 2012 Oct 22
Publication date: 2012
Volume: 15
Issue: Suppl 3
Electronic Location ID: 18442-130
Copyright: © 2012 N. Metheny et al.
Copyright year: 2012
License: This is an open-access article distributed under the terms of the Creative Commons Attribution License, which permits unrestricted use, distribution, and reproduction in any medium, provided the original work is properly cited.
License URL: http://creativecommons.org/licenses/by/2.0/

JOURNAL INFORMATION
==============================
NLM Title Abbreviation: J Int AIDS Soc
Journal ID: J Int AIDS Soc
NLM Journal ID: 101478566
Journal ID: JIAS

Journal of the International AIDS Society

EISSN: 1758-2652
Publisher: Wiley

ARTICLE INFORMATION
==============================
Article ID: 18442-131
Subjects: D93 - Intellectual property and trade regimes and access to HIV treatment and diagnostic medical devices

WEAE0102: Compulsory licence and access to medicines: economic savings of efavirenz in Brazil

Viegas Neves da Silva F. 1 §
Hallal R. 1
Guimarães A. 1
1 Ministry of Health, Brazil, Department of STD, AIDS and Viral Hepatitis of the Secretariat for Health Surveillance, Brasília, Brazil
§ Presenting author email: fvnsilva@gmail.com
Electronic publication date: 2012 Oct 22
Publication date: 2012
Volume: 15
Issue: Suppl 3
Electronic Location ID: 18442-131
Copyright: © 2012 F. Viegas Neves da Silva et al.
Copyright year: 2012
License: This is an open-access article distributed under the terms of the Creative Commons Attribution License, which permits unrestricted use, distribution, and reproduction in any medium, provided the original work is properly cited.
License URL: http://creativecommons.org/licenses/by/2.0/

JOURNAL INFORMATION
==============================
NLM Title Abbreviation: J Int AIDS Soc
Journal ID: J Int AIDS Soc
NLM Journal ID: 101478566
Journal ID: JIAS

Journal of the International AIDS Society

EISSN: 1758-2652
Publisher: Wiley

ARTICLE INFORMATION
==============================
Article ID: 18442-132
Subjects: D94 - Human rights and legal protection and empowerment of PLWHA and key populations at higher risk

MOAD0205: Human right as a key component of harm reduction strategy targeting people using drugs in Morocco

Himmich H. 1 §
Maguet O. 2
Essalhi M. 3
Caldéron C. 2
Alaoui Hasnouni R. 4
Kandil A. 5
1 Association de Lutte Contre le Sida (ALCS), Casablanca, Morocco
2 CCMO Conseil, Paris, France
3 RDR Maroc, Tanger, Morocco
4 Association de Lutte Contre le Sida (ALCS), Tétouan, Morocco
5 Association de Lutte Contre le Sida (ALCS), Nador, Morocco
§ Presenting author email: h.himmich@gmail.com
Electronic publication date: 2012 Oct 22
Publication date: 2012
Volume: 15
Issue: Suppl 3
Electronic Location ID: 18442-132
Copyright: © 2012 H. Himmich et al.
Copyright year: 2012
License: This is an open-access article distributed under the terms of the Creative Commons Attribution License, which permits unrestricted use, distribution, and reproduction in any medium, provided the original work is properly cited.
License URL: http://creativecommons.org/licenses/by/2.0/

JOURNAL INFORMATION
==============================
NLM Title Abbreviation: J Int AIDS Soc
Journal ID: J Int AIDS Soc
NLM Journal ID: 101478566
Journal ID: JIAS

Journal of the International AIDS Society

EISSN: 1758-2652
Publisher: Wiley

ARTICLE INFORMATION
==============================
Article ID: 18442-133
Subjects: D94 - Human rights and legal protection and empowerment of PLWHA and key populations at higher risk

TUAD0104: Model of human rights protection of sex workers exposed to forced HIV/STI testing through combination of court litigation and psycho-social support

Shterjova Simonovikj H. 1 §
Tosheva M. 2
1 NGO HOPS ‘Healthy Option Project Skopje, Support for Sex Workers, Skopje, Macedonia, FYR
2 NGO HOPS ‘Healthy Option Project Skopje, Support for Sex Workers and their Families, Skopje, Macedonia, FYR
§ Presenting author email: sterjova@yahoo.com
Electronic publication date: 2012 Oct 22
Publication date: 2012
Volume: 15
Issue: Suppl 3
Electronic Location ID: 18442-133
Copyright: © 2012 H. Shterjova Simonovikj et al.
Copyright year: 2012
License: This is an open-access article distributed under the terms of the Creative Commons Attribution License, which permits unrestricted use, distribution, and reproduction in any medium, provided the original work is properly cited.
License URL: http://creativecommons.org/licenses/by/2.0/

JOURNAL INFORMATION
==============================
NLM Title Abbreviation: J Int AIDS Soc
Journal ID: J Int AIDS Soc
NLM Journal ID: 101478566
Journal ID: JIAS

Journal of the International AIDS Society

EISSN: 1758-2652
Publisher: Wiley

ARTICLE INFORMATION
==============================
Article ID: 18442-134
Subjects: D94 - Human rights and legal protection and empowerment of PLWHA and key populations at higher risk

WEAD0203: When sex is a crime: ending the criminalization of HIV-positive women's sexuality in the United States by using grass roots advocacy and leadership development to eliminate criminal HIV exposure and transmission laws

Kelly B. 1 §
Roose-Snyder B. 2
Johnson V. 3
Hanssens C. 2
Positive Justice Project
1 U.S. Positive Women's Network, a Project of WORLD, New York, United States
2 Center for HIV Law and Policy, New York, United States
3 National Women and AIDS Collective, Washington, United States
§ Presenting author email: bkelly@womenhiv.org
Electronic publication date: 2012 Oct 22
Publication date: 2012
Volume: 15
Issue: Suppl 3
Electronic Location ID: 18442-134
Copyright: © 2012 B. Kelly et al.
Copyright year: 2012
License: This is an open-access article distributed under the terms of the Creative Commons Attribution License, which permits unrestricted use, distribution, and reproduction in any medium, provided the original work is properly cited.
License URL: http://creativecommons.org/licenses/by/2.0/

JOURNAL INFORMATION
==============================
NLM Title Abbreviation: J Int AIDS Soc
Journal ID: J Int AIDS Soc
NLM Journal ID: 101478566
Journal ID: JIAS

Journal of the International AIDS Society

EISSN: 1758-2652
Publisher: Wiley

ARTICLE INFORMATION
==============================
Article ID: 18442-135
Subjects: D94 - Human rights and legal protection and empowerment of PLWHA and key populations at higher risk

WEAD0205: Advocating against the criminalization of HIV non-disclosure: an analysis of community-led, science-based criminal law reform in Ontario, Canada

Mykhalovskiy E. 1 §
Peck R. 2
Kazatchkine C. 3
McCaskell T. 4
Betteridge G. 5
1 York University, Toronto, Canada
2 HIV & AIDS Legal Clinic (Ontario), Toronto, Canada
3 Canadian HIV/AIDS Legal Network, Toronto, Canada
4 Ontario Working Group on Criminal Law and HIV Exposure, Toronto, Canada
5 Legal and Policy Consulting, Toronto, Canada
§ Presenting author email: ericm@yorku.ca
Electronic publication date: 2012 Oct 22
Publication date: 2012
Volume: 15
Issue: Suppl 3
Electronic Location ID: 18442-135
Copyright: © 2012 E. Mykhalovskiy et al.
Copyright year: 2012
License: This is an open-access article distributed under the terms of the Creative Commons Attribution License, which permits unrestricted use, distribution, and reproduction in any medium, provided the original work is properly cited.
License URL: http://creativecommons.org/licenses/by/2.0/

JOURNAL INFORMATION
==============================
NLM Title Abbreviation: J Int AIDS Soc
Journal ID: J Int AIDS Soc
NLM Journal ID: 101478566
Journal ID: JIAS

Journal of the International AIDS Society

EISSN: 1758-2652
Publisher: Wiley

ARTICLE INFORMATION
==============================
Article ID: 18442-136
Subjects: D94 - Human rights and legal protection and empowerment of PLWHA and key populations at higher risk

THAD0203: A handbook on HIV, law and human rights for the judiciary: a critical tool in a human rights-based HIV response

Symington A. 1 §
Elliott R. 1
Kazatchkine C. 1
Ka Hon Chu S. 1
Golichenko M. 1
1 Canadian HIV/AIDS Legal Network, Toronto, Canada
§ Presenting author email: asymington@aidslaw.ca
Electronic publication date: 2012 Oct 22
Publication date: 2012
Volume: 15
Issue: Suppl 3
Electronic Location ID: 18442-136
Copyright: © 2012 A. Symington et al.
Copyright year: 2012
License: This is an open-access article distributed under the terms of the Creative Commons Attribution License, which permits unrestricted use, distribution, and reproduction in any medium, provided the original work is properly cited.
License URL: http://creativecommons.org/licenses/by/2.0/

JOURNAL INFORMATION
==============================
NLM Title Abbreviation: J Int AIDS Soc
Journal ID: J Int AIDS Soc
NLM Journal ID: 101478566
Journal ID: JIAS

Journal of the International AIDS Society

EISSN: 1758-2652
Publisher: Wiley

ARTICLE INFORMATION
==============================
Article ID: 18442-137
Subjects: D94 - Human rights and legal protection and empowerment of PLWHA and key populations at higher risk

THAD0204: Using courts to eliminate discrimination in women's property and inheritance laws in southern Africa

Patel P. 1 §
1 Southern Africa Litigation Centre, HIV Programme, Johannesburg, South Africa
§ Presenting author email: pritip@salc.org.za
Electronic publication date: 2012 Oct 22
Publication date: 2012
Volume: 15
Issue: Suppl 3
Electronic Location ID: 18442-137
Copyright: © 2012 P. Patel et al.
Copyright year: 2012
License: This is an open-access article distributed under the terms of the Creative Commons Attribution License, which permits unrestricted use, distribution, and reproduction in any medium, provided the original work is properly cited.
License URL: http://creativecommons.org/licenses/by/2.0/

JOURNAL INFORMATION
==============================
NLM Title Abbreviation: J Int AIDS Soc
Journal ID: J Int AIDS Soc
NLM Journal ID: 101478566
Journal ID: JIAS

Journal of the International AIDS Society

EISSN: 1758-2652
Publisher: Wiley

ARTICLE INFORMATION
==============================
Article ID: 18442-138
Subjects: D95 - Ethics (including research, clinical, public health and professional ethics)

MOPDD0401: The HPTN 052 HIV treatment as prevention trial: a case study of ethical considerations in human research conducted in low- and middle-income countries (LMIC)

Berger P. 1 2 §
1 University of Toronto, Faculty of Medicine, Toronto, Canada
2 St. Michael's Hospital, Family and Community Medicine, Toronto, Canada
§ Presenting author . Email: bergerp@smh.ca
Electronic publication date: 2012 Oct 22
Publication date: 2012
Volume: 15
Issue: Suppl 3
Electronic Location ID: 18442-138
Copyright: © 2012 P. Berger et al.
Copyright year: 2012
License: This is an open-access article distributed under the terms of the Creative Commons Attribution License, which permits unrestricted use, distribution, and reproduction in any medium, provided the original work is properly cited.
License URL: http://creativecommons.org/licenses/by/2.0/

JOURNAL INFORMATION
==============================
NLM Title Abbreviation: J Int AIDS Soc
Journal ID: J Int AIDS Soc
NLM Journal ID: 101478566
Journal ID: JIAS

Journal of the International AIDS Society

EISSN: 1758-2652
Publisher: Wiley

ARTICLE INFORMATION
==============================
Article ID: 18442-139
Subjects: D95 - Ethics (including research, clinical, public health and professional ethics)

MOPDD0402: Thought experiment examining the ethics of HIV testing policies

Schlecht H.P. 1 §
1 Drexel University College of Medicine, Infectious Diseases and HIV Medicine, Philadelphia, United States
§ Presenting author email: hans.schlecht@drexelmed.edu
Electronic publication date: 2012 Oct 22
Publication date: 2012
Volume: 15
Issue: Suppl 3
Electronic Location ID: 18442-139
Copyright: © 2012 H.P. Schlecht et al.
Copyright year: 2012
License: This is an open-access article distributed under the terms of the Creative Commons Attribution License, which permits unrestricted use, distribution, and reproduction in any medium, provided the original work is properly cited.
License URL: http://creativecommons.org/licenses/by/2.0/

JOURNAL INFORMATION
==============================
NLM Title Abbreviation: J Int AIDS Soc
Journal ID: J Int AIDS Soc
NLM Journal ID: 101478566
Journal ID: JIAS

Journal of the International AIDS Society

EISSN: 1758-2652
Publisher: Wiley

ARTICLE INFORMATION
==============================
Article ID: 18442-140
Subjects: D95 - Ethics (including research, clinical, public health and professional ethics)

MOPDD0403: Cost effectiveness of two transport strategies for retention of young mothers and infants enrolled in a phase IIb, TB vaccine clinical trial in Siaya, Kenya

Awuoche H. 1 2 §
Kiringa G. 1 2
Nduba V. 1 2
Mitchell E. 3
1 Kenya Medical Research Institute, Kisumu, Kenya
2 United States Centers for Disease Control and Prevention, Kisumu, Kenya
3 KNCV Dutch Tuberculosis Foundation, The Hague, Netherlands
§ Presenting author email: carolawuoche@gmail.com
Electronic publication date: 2012 Oct 22
Publication date: 2012
Volume: 15
Issue: Suppl 3
Electronic Location ID: 18442-140
Copyright: © 2012 H. Awuoche et al.
Copyright year: 2012
License: This is an open-access article distributed under the terms of the Creative Commons Attribution License, which permits unrestricted use, distribution, and reproduction in any medium, provided the original work is properly cited.
License URL: http://creativecommons.org/licenses/by/2.0/

JOURNAL INFORMATION
==============================
NLM Title Abbreviation: J Int AIDS Soc
Journal ID: J Int AIDS Soc
NLM Journal ID: 101478566
Journal ID: JIAS

Journal of the International AIDS Society

EISSN: 1758-2652
Publisher: Wiley

ARTICLE INFORMATION
==============================
Article ID: 18442-141
Subjects: D95 - Ethics (including research, clinical, public health and professional ethics)

MOPDD0404: Exploring research participants' perceptions and comprehension of the informed consent process in a pre-exposure HIV prevention study in Zimbabwe: a case study

Ruzario S. 1 2 §
Mavhu W. 3 4
Rossouw T. 5
1 Medical Reserach Council of Zimbabwe, Research Oversight, Harare, Zimbabwe
2 University of Pretoria, Pretoria, South Africa
3 ZAPP, Community Medicine, Harare, Zimbabwe
4 University of Zimbabwe, Community Medicine, Harare, Zimbabwe
5 Pretoria, Pretoria, South Africa
§ Presenting author email: sithembileruzario@yahoo.co.uk
Electronic publication date: 2012 Oct 22
Publication date: 2012
Volume: 15
Issue: Suppl 3
Electronic Location ID: 18442-141
Copyright: © 2012 S. Ruzario et al.
Copyright year: 2012
License: This is an open-access article distributed under the terms of the Creative Commons Attribution License, which permits unrestricted use, distribution, and reproduction in any medium, provided the original work is properly cited.
License URL: http://creativecommons.org/licenses/by/2.0/

JOURNAL INFORMATION
==============================
NLM Title Abbreviation: J Int AIDS Soc
Journal ID: J Int AIDS Soc
NLM Journal ID: 101478566
Journal ID: JIAS

Journal of the International AIDS Society

EISSN: 1758-2652
Publisher: Wiley

ARTICLE INFORMATION
==============================
Article ID: 18442-142
Subjects: D95 - Ethics (including research, clinical, public health and professional ethics)

MOPDD0405: ‘It doesn't work that way around here': lessons learned from an HIV community-based research study conducted in a hospital setting

Guta A. 1
Switzer S. 2 §
de Prinse K. 2
Chan Carusone S. 2
Strike C. 1
1 University of Toronto, Dalla Lana School of Public Health, Toronto, Canada
2 Casey House Hospital, Toronto, Canada
§ Presenting author email: sswitzer@caseyhouse.on.ca
Electronic publication date: 2012 Oct 22
Publication date: 2012
Volume: 15
Issue: Suppl 3
Electronic Location ID: 18442-142
Copyright: © 2012 S. Switzer et al.
Copyright year: 2012
License: This is an open-access article distributed under the terms of the Creative Commons Attribution License, which permits unrestricted use, distribution, and reproduction in any medium, provided the original work is properly cited.
License URL: http://creativecommons.org/licenses/by/2.0/

JOURNAL INFORMATION
==============================
NLM Title Abbreviation: J Int AIDS Soc
Journal ID: J Int AIDS Soc
NLM Journal ID: 101478566
Journal ID: JIAS

Journal of the International AIDS Society

EISSN: 1758-2652
Publisher: Wiley

ARTICLE INFORMATION
==============================
Article ID: 18442-143
Subjects: D96 - Children's rights and HIV

MOAD0101: Implementation of a model of psychosocial approach to strengthen HIV/AIDS treatment adherence of children and adolescents in south Lima

Salazar Ramirez D. 1 §
Salazar P. 1
Garcia Cordova A. 1
Villani J. 1
1 Coloreando Vidas, Lima, Lima, Peru
§ Presenting author email: desara20002000@yahoo.es
Electronic publication date: 2012 Oct 22
Publication date: 2012
Volume: 15
Issue: Suppl 3
Electronic Location ID: 18442-143
Copyright: © 2012 D. Salazar Ramirez et al.
Copyright year: 2012
License: This is an open-access article distributed under the terms of the Creative Commons Attribution License, which permits unrestricted use, distribution, and reproduction in any medium, provided the original work is properly cited.
License URL: http://creativecommons.org/licenses/by/2.0/

JOURNAL INFORMATION
==============================
NLM Title Abbreviation: J Int AIDS Soc
Journal ID: J Int AIDS Soc
NLM Journal ID: 101478566
Journal ID: JIAS

Journal of the International AIDS Society

EISSN: 1758-2652
Publisher: Wiley

ARTICLE INFORMATION
==============================
Article ID: 18442-144
Subjects: D98 - Punitive laws and practices around HIV transmission, sex work, drug use and homosexuality

MOAD0201: Tougher policy on French illegal drug users: what impact on risk reduction?

Geffroy L. 1 §
Lowenstein W. 1
Bourdillon F. 1
Celse M. 1
Rozenbaum W. 1
1 French National AIDS Council, Paris, France
§ Presenting author email: laurent.geffroy@sante.gouv.fr
Electronic publication date: 2012 Oct 22
Publication date: 2012
Volume: 15
Issue: Suppl 3
Electronic Location ID: 18442-144
Copyright: © 2012 L. Geffroy et al.
Copyright year: 2012
License: This is an open-access article distributed under the terms of the Creative Commons Attribution License, which permits unrestricted use, distribution, and reproduction in any medium, provided the original work is properly cited.
License URL: http://creativecommons.org/licenses/by/2.0/

JOURNAL INFORMATION
==============================
NLM Title Abbreviation: J Int AIDS Soc
Journal ID: J Int AIDS Soc
NLM Journal ID: 101478566
Journal ID: JIAS

Journal of the International AIDS Society

EISSN: 1758-2652
Publisher: Wiley

ARTICLE INFORMATION
==============================
Article ID: 18442-145
Subjects: D98 - Punitive laws and practices around HIV transmission, sex work, drug use and homosexuality

TUAD0102: Tightening the laws on prostitution in France: what impact on prevention?

Geffroy L. 1 §
Bourdillon F. 1
Musso S. 1
Hesnault-Pruniaux N. 1
Dozon J.-P. 1
Cadoré B. 1
Briand Y. 1
Celse M. 1
Rozenbaum W. 1
1 French National AIDS Council, Paris, France
§ Presenting author email: laurent.geffroy@sante.gouv.fr
Electronic publication date: 2012 Oct 22
Publication date: 2012
Volume: 15
Issue: Suppl 3
Electronic Location ID: 18442-145
Copyright: © 2012 L. Geffroy et al.
Copyright year: 2012
License: This is an open-access article distributed under the terms of the Creative Commons Attribution License, which permits unrestricted use, distribution, and reproduction in any medium, provided the original work is properly cited.
License URL: http://creativecommons.org/licenses/by/2.0/

JOURNAL INFORMATION
==============================
NLM Title Abbreviation: J Int AIDS Soc
Journal ID: J Int AIDS Soc
NLM Journal ID: 101478566
Journal ID: JIAS

Journal of the International AIDS Society

EISSN: 1758-2652
Publisher: Wiley

ARTICLE INFORMATION
==============================
Article ID: 18442-146
Subjects: D98 - Punitive laws and practices around HIV transmission, sex work, drug use and homosexuality

TUAD0103: Criminalization of condoms in the United States: why sex workers and HIV services are at risk

McLemore M. 1 §
1 Human Rights Watch, Health and Human Rights, New York, United States
§ Presenting author email: mclemom@hrw.org
Electronic publication date: 2012 Oct 22
Publication date: 2012
Volume: 15
Issue: Suppl 3
Electronic Location ID: 18442-146
Copyright: © 2012 M. McLemore et al.
Copyright year: 2012
License: This is an open-access article distributed under the terms of the Creative Commons Attribution License, which permits unrestricted use, distribution, and reproduction in any medium, provided the original work is properly cited.
License URL: http://creativecommons.org/licenses/by/2.0/

JOURNAL INFORMATION
==============================
NLM Title Abbreviation: J Int AIDS Soc
Journal ID: J Int AIDS Soc
NLM Journal ID: 101478566
Journal ID: JIAS

Journal of the International AIDS Society

EISSN: 1758-2652
Publisher: Wiley

ARTICLE INFORMATION
==============================
Article ID: 18442-147
Subjects: D98 - Punitive laws and practices around HIV transmission, sex work, drug use and homosexuality

WEAD0201: Criminal prosecutions for HIV non-disclosure, exposure and transmission: overview and updated global ranking

Bernard E.J. 1 2 §
Nyambe M. 3
1 HIV Justice Network, Berlin, Germany
2 Criminal HIV Transmission (blog), Brighton, United Kingdom
3 Global Network of People Living with HIV (GNP+), Amsterdam, Netherlands
§ Presenting author email: edwin@edwinjbernard.com
Electronic publication date: 2012 Oct 22
Publication date: 2012
Volume: 15
Issue: Suppl 3
Electronic Location ID: 18442-147
Copyright: © 2012 E.J. Bernard et al.
Copyright year: 2012
License: This is an open-access article distributed under the terms of the Creative Commons Attribution License, which permits unrestricted use, distribution, and reproduction in any medium, provided the original work is properly cited.
License URL: http://creativecommons.org/licenses/by/2.0/

JOURNAL INFORMATION
==============================
NLM Title Abbreviation: J Int AIDS Soc
Journal ID: J Int AIDS Soc
NLM Journal ID: 101478566
Journal ID: JIAS

Journal of the International AIDS Society

EISSN: 1758-2652
Publisher: Wiley

ARTICLE INFORMATION
==============================
Article ID: 18442-148
Subjects: D98 - Punitive laws and practices around HIV transmission, sex work, drug use and homosexuality

WEAD0202: The impact of HIV/AIDS criminalization on awareness, prevention and stigma in the: a qualitative analysis of stakeholders' perspectives in Ontario, Canada

Lax-Vanek J. 1 §
Rans S. 1
Greene B. 1
Chung K. 1
Shorkey A. 1
Wilson M. 2
1 McMaster University, Hamilton, Canada
2 McMaster University, Program in Policy and Decision Making, Hamilton, Canada
§ Presenting author email: laxvanjt@mcmaster.ca
Electronic publication date: 2012 Oct 22
Publication date: 2012
Volume: 15
Issue: Suppl 3
Electronic Location ID: 18442-148
Copyright: © 2012 J. Lax-Vanek et al.
Copyright year: 2012
License: This is an open-access article distributed under the terms of the Creative Commons Attribution License, which permits unrestricted use, distribution, and reproduction in any medium, provided the original work is properly cited.
License URL: http://creativecommons.org/licenses/by/2.0/

JOURNAL INFORMATION
==============================
NLM Title Abbreviation: J Int AIDS Soc
Journal ID: J Int AIDS Soc
NLM Journal ID: 101478566
Journal ID: JIAS

Journal of the International AIDS Society

EISSN: 1758-2652
Publisher: Wiley

ARTICLE INFORMATION
==============================
Article ID: 18442-149
Subjects: D98 - Punitive laws and practices around HIV transmission, sex work, drug use and homosexuality

WEAD0204: Punishing HIV: how Michigan trial courts frame criminal HIV disclosure cases

Hoppe T. 1 §
1 University of Michigan, Sociology, Ann Arbor, United States
§ Presenting author email: thoppe@umich.edu
Electronic publication date: 2012 Oct 22
Publication date: 2012
Volume: 15
Issue: Suppl 3
Electronic Location ID: 18442-149
Copyright: © 2012 T. Hoppe et al.
Copyright year: 2012
License: This is an open-access article distributed under the terms of the Creative Commons Attribution License, which permits unrestricted use, distribution, and reproduction in any medium, provided the original work is properly cited.
License URL: http://creativecommons.org/licenses/by/2.0/

JOURNAL INFORMATION
==============================
NLM Title Abbreviation: J Int AIDS Soc
Journal ID: J Int AIDS Soc
NLM Journal ID: 101478566
Journal ID: JIAS

Journal of the International AIDS Society

EISSN: 1758-2652
Publisher: Wiley

ARTICLE INFORMATION
==============================
Article ID: 18442-150
Subjects: D98 - Punitive laws and practices around HIV transmission, sex work, drug use and homosexuality

THAD0201: Knowledge and experience of HIV and the law amongst global key populations: global focus group feedback and recommendations for policy change

Simon S. 1 §
1 ICASO, UNAIDS PCB NGO Delegation CF, Brussels, Belgium
§ Presenting author email: saraesimon@gmail.com
Electronic publication date: 2012 Oct 22
Publication date: 2012
Volume: 15
Issue: Suppl 3
Electronic Location ID: 18442-150
Copyright: © 2012 S. Simon et al.
Copyright year: 2012
License: This is an open-access article distributed under the terms of the Creative Commons Attribution License, which permits unrestricted use, distribution, and reproduction in any medium, provided the original work is properly cited.
License URL: http://creativecommons.org/licenses/by/2.0/

JOURNAL INFORMATION
==============================
NLM Title Abbreviation: J Int AIDS Soc
Journal ID: J Int AIDS Soc
NLM Journal ID: 101478566
Journal ID: JIAS

Journal of the International AIDS Society

EISSN: 1758-2652
Publisher: Wiley

ARTICLE INFORMATION
==============================
Article ID: 18442-151
Subjects: D98 - Punitive laws and practices around HIV transmission, sex work, drug use and homosexuality

THAD0205: Making multi-collaborative partnerships work and worth it: experience in working with Philippine National Police and Quezon City Police Department to address the use of condoms as evidence of prostitution among sex workers

Acaba J. 1 §
1 Action for Health Initiatives (ACHIEVE), Inc., Quezon City, Philippines
§ Presenting author email: jpacaba@gmail.com
Electronic publication date: 2012 Oct 22
Publication date: 2012
Volume: 15
Issue: Suppl 3
Electronic Location ID: 18442-151
Copyright: © 2012 J. Acaba et al.
Copyright year: 2012
License: This is an open-access article distributed under the terms of the Creative Commons Attribution License, which permits unrestricted use, distribution, and reproduction in any medium, provided the original work is properly cited.
License URL: http://creativecommons.org/licenses/by/2.0/

JOURNAL INFORMATION
==============================
NLM Title Abbreviation: J Int AIDS Soc
Journal ID: J Int AIDS Soc
NLM Journal ID: 101478566
Journal ID: JIAS

Journal of the International AIDS Society

EISSN: 1758-2652
Publisher: Wiley

ARTICLE INFORMATION
==============================
Article ID: 18442-152
Subjects: D98 - Punitive laws and practices around HIV transmission, sex work, drug use and homosexuality

MOPDD0202: Condoms as evidence: police, sex workers and condom confiscation in Zimbabwe

Maseko S. 1 §
Ndlovu S. 1
1 Sexual Rights Centre, Bulawayo, Zimbabwe
§ Presenting author email: director_cad@yahoo.com
Electronic publication date: 2012 Oct 22
Publication date: 2012
Volume: 15
Issue: Suppl 3
Electronic Location ID: 18442-152
Copyright: © 2012 S. Maseko et al.
Copyright year: 2012
License: This is an open-access article distributed under the terms of the Creative Commons Attribution License, which permits unrestricted use, distribution, and reproduction in any medium, provided the original work is properly cited.
License URL: http://creativecommons.org/licenses/by/2.0/

JOURNAL INFORMATION
==============================
NLM Title Abbreviation: J Int AIDS Soc
Journal ID: J Int AIDS Soc
NLM Journal ID: 101478566
Journal ID: JIAS

Journal of the International AIDS Society

EISSN: 1758-2652
Publisher: Wiley

ARTICLE INFORMATION
==============================
Article ID: 18442-153
Subjects: D98 - Punitive laws and practices around HIV transmission, sex work, drug use and homosexuality

MOPDD0203: The impact of laws criminalizing commercial sex clients and third parties on the health and safety of sex workers based on the street and in drug venues: a Canadian case study

Caouette A.-A. 1
Schoepp A. 1
1 Stella, l'amie de Maimie, Montreal, Canada
Electronic publication date: 2012 Oct 22
Publication date: 2012
Volume: 15
Issue: Suppl 3
Electronic Location ID: 18442-153
Copyright: © 2012 et al.
Copyright year: 2012
License: This is an open-access article distributed under the terms of the Creative Commons Attribution License, which permits unrestricted use, distribution, and reproduction in any medium, provided the original work is properly cited.
License URL: http://creativecommons.org/licenses/by/2.0/

JOURNAL INFORMATION
==============================
NLM Title Abbreviation: J Int AIDS Soc
Journal ID: J Int AIDS Soc
NLM Journal ID: 101478566
Journal ID: JIAS

Journal of the International AIDS Society

EISSN: 1758-2652
Publisher: Wiley

ARTICLE INFORMATION
==============================
Article ID: 18442-154
Subjects: D105 - Human and food security

MOPDD0301: Food insecurity and HIV: concepts and measurement tools in the HAART era

Fielden S. 1 2 §
Fergusson P. 3
Muldoon K. 4
Anema A. 4
1 University of British Columbia, School of Population and Public Health, Vancouver, Canada
2 Université du Québec à Montréal, Montréal, Canada
3 University College London, Centre for International Health and Development, Institute of Child Health, London, United Kingdom
4 BC Center for Excellence in HIV/AIDS, University of British Columbia, Vancouver, Canada
§ Presenting author email: sjfielden@yahoo.ca
Electronic publication date: 2012 Oct 22
Publication date: 2012
Volume: 15
Issue: Suppl 3
Electronic Location ID: 18442-154
Copyright: © 2012 S. Fielden et al.
Copyright year: 2012
License: This is an open-access article distributed under the terms of the Creative Commons Attribution License, which permits unrestricted use, distribution, and reproduction in any medium, provided the original work is properly cited.
License URL: http://creativecommons.org/licenses/by/2.0/

JOURNAL INFORMATION
==============================
NLM Title Abbreviation: J Int AIDS Soc
Journal ID: J Int AIDS Soc
NLM Journal ID: 101478566
Journal ID: JIAS

Journal of the International AIDS Society

EISSN: 1758-2652
Publisher: Wiley

ARTICLE INFORMATION
==============================
Article ID: 18442-155
Subjects: D105 - Human and food security

MOPDD0302: Severe household food insecurity is highly prevalent and associated with suboptimal breastfeeding practices among HIV-positive women in rural Uganda

Young S.L. 1 §
Natamba B.K. 1
Luwedde F.A. 2
Plenty A.H.J. 3
Mwesigwa J. 2
Natureeba P. 2
Osterbauer B. 4
Achan J. 5
Clark T.D. 4
Ades V. 6
Robine M. 1
Nzarubara B.K. 2
Ruel T.D. 7
Kamya M. 8
Charlebois E.D. 3
Havlir D.V. 4
Cohan D.L. 6
1 Cornell University, Division of Nutritional Sciences, Ithaca, United States
2 Makerere University-University of California, San Francisco (MU-UCSF) Research Collaboration, Tororo, Uganda
3 University of California at San Francisco, Center for AIDS Prevention Studies, San Francisco, United States
4 University of California at San Francisco, Department of Medicine, San Francisco, United States
5 Makerere University College of Health Sciences, Department of Pediatrics and Child Health, Kampala, Uganda
6 University of California at San FranciscoGynecology, and Reproductive Sciences, Department of Obstetrics, San Francisco, United States
7 University of California at San Francisco, Department of Pediatrics, San Francisco, United States
8 Makerere University Medical School, Department of Medicine, Kampala, Uganda
§ Presenting author email: sera.young@cornell.edu
Electronic publication date: 2012 Oct 22
Publication date: 2012
Volume: 15
Issue: Suppl 3
Electronic Location ID: 18442-155
Copyright: © 2012 S.L. Young et al.
Copyright year: 2012
License: This is an open-access article distributed under the terms of the Creative Commons Attribution License, which permits unrestricted use, distribution, and reproduction in any medium, provided the original work is properly cited.
License URL: http://creativecommons.org/licenses/by/2.0/

JOURNAL INFORMATION
==============================
NLM Title Abbreviation: J Int AIDS Soc
Journal ID: J Int AIDS Soc
NLM Journal ID: 101478566
Journal ID: JIAS

Journal of the International AIDS Society

EISSN: 1758-2652
Publisher: Wiley

ARTICLE INFORMATION
==============================
Article ID: 18442-156
Subjects: D105 - Human and food security

MOPDD0305: Impact of food assistance on clinic attendance among patients on antiretroviral therapy in Haiti

Bernard D. 1 §
Koenig S.P. 2
Riviere C. 1
Bertil C. 1
Severe P. 1
Pape J.W. 1 3
1 The GHESKIO Centers, Port-au-Prince, Haiti
2 Division of Global Health Equity Brigham and Women's Hospital/Harvard Medical School Boston, Boston, United States
3 Division of Global Health, Weil Medical School of Cornel University, New York, United States
§ Presenting author email: daphnebernard17@gmail.com
Electronic publication date: 2012 Oct 22
Publication date: 2012
Volume: 15
Issue: Suppl 3
Electronic Location ID: 18442-156
Copyright: © 2012 D. Bernard et al.
Copyright year: 2012
License: This is an open-access article distributed under the terms of the Creative Commons Attribution License, which permits unrestricted use, distribution, and reproduction in any medium, provided the original work is properly cited.
License URL: http://creativecommons.org/licenses/by/2.0/

JOURNAL INFORMATION
==============================
NLM Title Abbreviation: J Int AIDS Soc
Journal ID: J Int AIDS Soc
NLM Journal ID: 101478566
Journal ID: JIAS

Journal of the International AIDS Society

EISSN: 1758-2652
Publisher: Wiley

ARTICLE INFORMATION
==============================
Article ID: 18442-157
Subjects: D106 - Disasters and HIV

THPDD0101: Access to HIV prevention, care and treatment in refugee camps setting in 21 countries: review of key indicators

Cornier N. 1 §
Schilperoord M. 1
Spiegel P. 1
Haskew C. 1
Njogu P. 2
Yiweza D. 3
Doraiswamy S. 4
1 UNHCR, Geneva, Switzerland
2 UNHCR, Pretoria, South Africa
3 UNHCR, Dakar, Senegal
4 UNHCR, Nairobi, Kenya
§ Presenting author email: cornier@unhcr.org
Electronic publication date: 2012 Oct 22
Publication date: 2012
Volume: 15
Issue: Suppl 3
Electronic Location ID: 18442-157
Copyright: © 2012 N. Cornier et al.
Copyright year: 2012
License: This is an open-access article distributed under the terms of the Creative Commons Attribution License, which permits unrestricted use, distribution, and reproduction in any medium, provided the original work is properly cited.
License URL: http://creativecommons.org/licenses/by/2.0/

JOURNAL INFORMATION
==============================
NLM Title Abbreviation: J Int AIDS Soc
Journal ID: J Int AIDS Soc
NLM Journal ID: 101478566
Journal ID: JIAS

Journal of the International AIDS Society

EISSN: 1758-2652
Publisher: Wiley

ARTICLE INFORMATION
==============================
Article ID: 18442-158
Subjects: D106 - Disasters and HIV

THPDD0102: Poor treatment outcomes among both refugees and host community accessing highly active antiretroviral therapy (HAART) from Kakuma refugee camp in northwestern Kenya

Mendelsohn J.B. 1 §
Schilperoord M. 2
Spiegel P. 2
Burton J.W. 3
Okonji J.A. 4
Muhindo B. 5
Njogu P. 6
Larke N. 7
Grant A. 8
Mohamed I.M. 9
Mukui I.N. 9
Ross D.A. 10
1 London School of Hygiene and Tropical Medicine, Infectious Disease Epidemiology, London, United Kingdom
2 United Nations High Commissioner for Refugees, Public Health and HIV Section, Geneva, Switzerland
3 United Nations High Commissioner for Refugees, Nairobi, Kenya
4 Kenya Medical Research Institute (KEMRI), Kisumu, Kenya
5 United Nations High Commissioner for Refugees, Kakuma Sub-Office, Kakuma, Kenya
6 United Nations High Commissioner for Refugees, Regional Office, Nairobi, Kenya
7 London School of Hygiene & Tropical Medicine, Department of Infectious Disease Epidemiology, London, United Kingdom
8 London School of Hygiene & Tropical Medicine, Department of Clinical Research, London, United Kingdom
9 National AIDS and STD Control Programme (NASCOP), Nairobi, Kenya
0 London School of Hygiene & Tropical Medicine, MRC Tropical Epidemiology Group, Department of Infectious Disease Epidemiology, London, United Kingdom
§ Presenting author email: joshua.mendelsohn@lshtm.ac.uk
Electronic publication date: 2012 Oct 22
Publication date: 2012
Volume: 15
Issue: Suppl 3
Electronic Location ID: 18442-158
Copyright: © 2012 J.B. Mendelsohn et al.
Copyright year: 2012
License: This is an open-access article distributed under the terms of the Creative Commons Attribution License, which permits unrestricted use, distribution, and reproduction in any medium, provided the original work is properly cited.
License URL: http://creativecommons.org/licenses/by/2.0/

JOURNAL INFORMATION
==============================
NLM Title Abbreviation: J Int AIDS Soc
Journal ID: J Int AIDS Soc
NLM Journal ID: 101478566
Journal ID: JIAS

Journal of the International AIDS Society

EISSN: 1758-2652
Publisher: Wiley

ARTICLE INFORMATION
==============================
Article ID: 18442-159
Subjects: D106 - Disasters and HIV

THPDD0103: Acceptable adherence and treatment outcomes among refugees and host community on highly active antiretroviral therapy (HAART) in an urban refugee setting in Kuala Lumpur, Malaysia

Mendelsohn J.B. 1 §
Spiegel P. 2
Schilperoord M. 2
Balasundaram S. 3
Radhakrishnan A. 4
Lee C. 4
Larke N. 5
Grant A. 6
Ross D.A. 1
1 London School of Hygiene & Tropical Medicine, MRC Tropical Epidemiology Group, Department of Infectious Disease Epidemiology, London, United Kingdom
2 United Nations High Commissioner for Refugees, Public Health and HIV Section, Geneva, Switzerland
3 United Nations High Commissioner for Refugees, Kuala Lumpur, Malaysia
4 Hospital Sungai Buloh, Infectious Disease Unit, Department of Medicine, Sungai Buloh, Malaysia
5 London School of Hygiene & Tropical Medicine, Department of Infectious Disease Epidemiology, London, United Kingdom
6 London School of Hygiene & Tropical Medicine, Department of Clinical Research, London, United Kingdom
§ Presenting author email: joshua.mendelsohn@lshtm.ac.uk
Electronic publication date: 2012 Oct 22
Publication date: 2012
Volume: 15
Issue: Suppl 3
Electronic Location ID: 18442-159
Copyright: © 2012 J.B. Mendelsohn et al.
Copyright year: 2012
License: This is an open-access article distributed under the terms of the Creative Commons Attribution License, which permits unrestricted use, distribution, and reproduction in any medium, provided the original work is properly cited.
License URL: http://creativecommons.org/licenses/by/2.0/

JOURNAL INFORMATION
==============================
NLM Title Abbreviation: J Int AIDS Soc
Journal ID: J Int AIDS Soc
NLM Journal ID: 101478566
Journal ID: JIAS

Journal of the International AIDS Society

EISSN: 1758-2652
Publisher: Wiley

ARTICLE INFORMATION
==============================
Article ID: 18442-160
Subjects: D106 - Disasters and HIV

THPDE0205: Adherence to highly active antiretroviral therapy and treatment outcomes in conflict-affected and forcibly displaced populations: a systematic review

Mendelsohn J.B. 1 §
Spiegel P. 2
Schilperoord M. 2
Njogu P.M. 3
Ross D.A. 1
1 London School of Hygiene & Tropical Medicine, MRC Tropical Epidemiology Group, Department of Infectious Disease Epidemiology, London, United Kingdom
2 United Nations High Commissioner for Refugees, Public Health and HIV Section, Geneva, Switzerland
3 United Nations High Commisoner for Refugees, Pretoria, South Africa
P.M. Njogu,
Electronic publication date: 2012 Oct 22
Publication date: 2012
Volume: 15
Issue: Suppl 3
Electronic Location ID: 18442-160
Copyright: © 2012 P.M. Njogu et al.
Copyright year: 2012
License: This is an open-access article distributed under the terms of the Creative Commons Attribution License, which permits unrestricted use, distribution, and reproduction in any medium, provided the original work is properly cited.
License URL: http://creativecommons.org/licenses/by/2.0/

JOURNAL INFORMATION
==============================
NLM Title Abbreviation: J Int AIDS Soc
Journal ID: J Int AIDS Soc
NLM Journal ID: 101478566
Journal ID: JIAS

Journal of the International AIDS Society

EISSN: 1758-2652
Publisher: Wiley

ARTICLE INFORMATION
==============================
Article ID: 18442-161
Subjects: D109 - Education and child welfare

MOPDD0304: HIV-related orphans and vulnerable children: schooling, nutrition and community support among three provinces in Cambodia

Tuot S. 1 §
Sopheab H. 1
Oum S. 2
Connelly C. 3
1 KHANA, Research Department for HIV, Health and Development, Phnom Penh, Cambodia
2 KHANA, Executive Director Office, Phnom Penh, Cambodia
3 KHANA, Technical Support and Best Practices, Phnom Penh, Cambodia
§ Presenting author email: tsovannary@khana.org.kh
Electronic publication date: 2012 Oct 22
Publication date: 2012
Volume: 15
Issue: Suppl 3
Electronic Location ID: 18442-161
Copyright: © 2012 S. Tuot et al.
Copyright year: 2012
License: This is an open-access article distributed under the terms of the Creative Commons Attribution License, which permits unrestricted use, distribution, and reproduction in any medium, provided the original work is properly cited.
License URL: http://creativecommons.org/licenses/by/2.0/

JOURNAL INFORMATION
==============================
NLM Title Abbreviation: J Int AIDS Soc
Journal ID: J Int AIDS Soc
NLM Journal ID: 101478566
Journal ID: JIAS

Journal of the International AIDS Society

EISSN: 1758-2652
Publisher: Wiley

ARTICLE INFORMATION
==============================
Article ID: 18442-162
Subjects: D110 - Social welfare

WEPDD0302: Disability grant terminations and virologic and immunologic response to ARV treatment

Booysen F. 1 §
De Walque D. 2
Over M. 3
1 University of the Free State (UFS), Economics, Bloemfontein, South Africa
2 The World Bank, Development Economics Research Group (DERG), Washington, United States
3 Center for Global Development, Washington, United States
§ Presenting author email: booysenfrikkie@gmail.com
Electronic publication date: 2012 Oct 22
Publication date: 2012
Volume: 15
Issue: Suppl 3
Electronic Location ID: 18442-162
Copyright: © 2012 F. Booysen et al.
Copyright year: 2012
License: This is an open-access article distributed under the terms of the Creative Commons Attribution License, which permits unrestricted use, distribution, and reproduction in any medium, provided the original work is properly cited.
License URL: http://creativecommons.org/licenses/by/2.0/

